# IRAK4 autophosphorylation functions as a molecular switch to regulate death domain assembly and Myddosome maturation

**DOI:** 10.1038/s41467-026-78773-6

**Published:** 2026-10-21

**Authors:** Niranjan A. Srikanth, Rafael Deliz-Aguirre, Deepika Kumari Gola, Margaux Bilay, Elke Ziska, Ian R. Kelsall, Yogesh Kulathu, Marcus J. Taylor

**Affiliations:** 1 Max Planck Institute for Infection Biology, Chariteplatz 1, Berlin, Germany; 2 Department of Biology, Chemistry and Pharmacy, Freie Universität Berlin, Berlin, Germany; 3 Humboldt University of Berlin, Unter den Linden 6, Berlin, Germany; 4 MRC Protein Phosphorylation and Ubiquitylation Unit, School of Life Sciences, University of Dundee, Dundee, UK

**Keywords:** Cell signalling, Kinases, Molecular conformation

## Abstract

The organization of signaling proteins into multi-component complexes is essential for innate immune responses and is often achieved through the oligomerization of death domains (DDs). The Myddosome, composed of MyD88 and IRAK family kinases, exemplifies this mechanism. However, how DDs assemble in an ordered and regulated manner to form Myddosomes to control signaling output remains unclear. Here, we show that IRAK4 kinase activity and autophosphorylation are required for IRAK4:IRAK1 heterotypic DD interactions. We show that IRAK1 is autoinhibited by its kinase domain, and IRAK4 phosphorylation relieves this inhibition, enabling IRAK1 DD incorporation into Myddosomes. This phosphorylation-dependent switch similarly controls IRAK2/3 incorporation. Thus, IRAK4 autophosphorylation acts as an energy-dependent molecular switch that integrates phosphorylation with protein oligomerization to drive Myddosome maturation and signal transduction. This mechanism demonstrates how enzymatic activity can impose temporal and spatial control over macromolecular assembly to ensure directional and context-dependent inflammatory signaling.

## Abstract (web summary)

Inflammatory signalling depends on higher-order protein assemblies such as the Myddosome. Here, authors show that IRAK4 autophosphorylation is a molecular switch that relieves IRAK1/2/3 autoinhibition, driving Myddosome maturation.

## Introduction

Inflammatory signaling depends on the use of higher-order protein assemblies termed signalosomes^1,2^. Many of these signalosomes are composed of complex mixtures of death domain (DD) oligomers^3^. However, these mixtures have a nanoscale order in which DD oligomers are sequentially fused to other oligomers^4^. Therefore, dynamic assembly and consequently signaling must require a controlled sequence of homo and heterotypic DD interactions. While structural studies highlight the fine scale atomic level features that bind DDs together, how these complexes dynamically assemble in response to signals is less well understood. Given that DD signalosomes activate powerful effector functions like cell death or the release of inflammatory cytokines, assembly must be highly controlled with multiple regulatory steps involved. These regulatory mechanisms are required to ensure a controlled and proportionate response and suppress assembly when cells are in a state of quiescence. Therefore, an essential question is how DD signalosome assembly is kinetically and spatially regulated to achieve downstream control.

Signalosomes composed of mixed oligomers of death domains (DDs) are exemplified by the Myddosome, which transduces signals from the IL-1 receptor^5,6^. Myddosome formation begins with the recruitment of MyD88 to the activated receptor, followed by controlled coassembly with IRAK family kinases. The core of this complex is built by the binding and oligomerization of IRAK4 on a platform of oligomerized MyD88. IRAK4 oligomers then serve as a platform for the binding and oligomerization of either IRAK1, IRAK2, or IRAK3^6,7^. This higher-order complex then engages TRAF6 to propagate downstream signaling^8^. Thus, an essential step in IL-1R signal transduction is controlled DD oligomerization and the co-assembly of IRAK kinases with MyD88 to ensure directed signal transduction. However, it remains unclear how the sequential incorporation of these components is regulated to achieve controlled activation of downstream signaling.

In many cases, DDs are fused to other effector domains. For example, in addition to an N-terminus DD, IRAKs also have a C-terminus kinase domain^9^. This raises the possibility that IRAK kinase domains regulate the ordered assembly of DDs into the Myddosome. Consistent with such a regulatory role, mutations in the IRAK4 kinase domain that inhibit its activity prevent co-immunoprecipitation with IRAK1 in stimulated cells^10^. Therefore, a critical question is how IRAK kinase domains regulate ordered Myddosome assembly. Here, we show that the assembly of heterotypic IRAK4 and IRAK1 DDs is regulated by their kinase domains. IRAK1 most likely exists in an autoinhibitory state where the kinase domain prevents DD co-assembly with the IRAK4 DD. IRAK4 autophosphorylation relieves IRAK1 autoinhibition by facilitating heterodimerization with the IRAK1 kinase domain, thereby allowing DD oligomerization. This mechanism of relieving autoinhibition most likely functions with IRAK2 and IRAK3, as inhibition of IRAK4 kinase activity also inhibits their incorporation into Myddosomes. Thus, IRAK4 autophosphorylation functions as an energy-dependent regulatory switch that controls the final stages of DD assembly and Myddosome maturation. Our work highlights how protein oligomerization can be regulated by enzymatic activity to ensure rapid, directed assembly of a signaling complex in response to upstream signals.

## Results

### IRAK4 and IRAK1 kinase domains differentially regulate heterotypic DD interactions

**Fig. 1:**
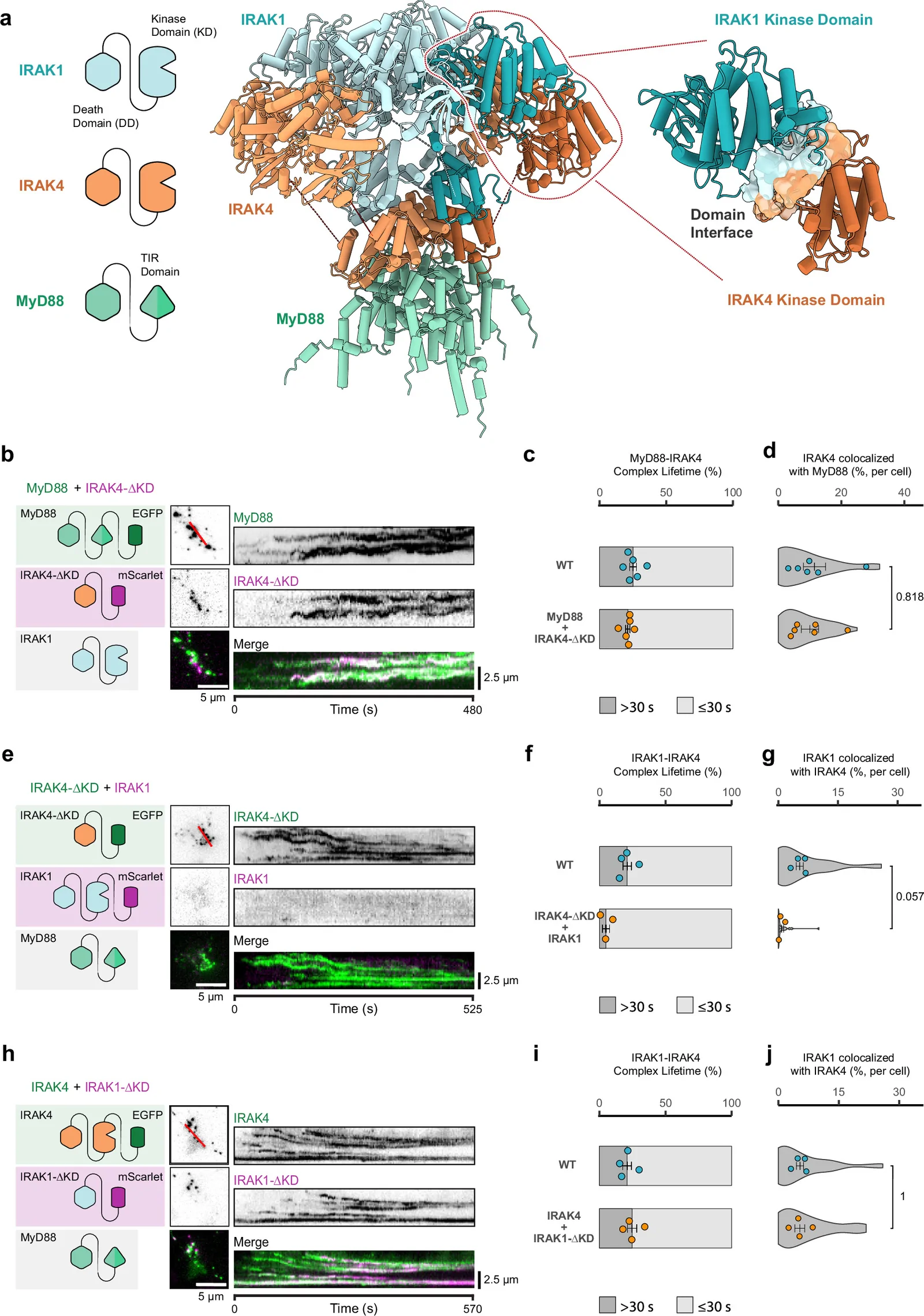
Differential regulation of heterotypic DD interactions in Myddosome assembly by IRAK4 and IRAK1 kinase domains. **a** AF3-predicted model of the Myddosome complex containing the DDs and kinase domains of IRAK4/1. Unstructured linker regions between the DD and kinase domain were removed and represented as a dashed line. Left, schematic domain organization of MyD88, IRAK4, and IRAK1. Right, AF3-predicted model of the Myddosome (6 × MyD88: 4 × IRAK4: 4 × IRAK1), with detail of the putative IRAK4–IRAK1 kinase domain interface (outlined in dashed red). The IRAK4/1 kinase domain heterodimer interface is highlighted. **b**–**d** Deletion of the IRAK4 kinase domain does not disrupt MyD88–IRAK4 assembly. **b** Representative TIRF image of EL4-MyD88-GFP/IRAK4-KO cells expressing IRAK4-ΔKD-mScarlet, with red line indicating the position used for kymograph generation (left panel). The corresponding kymograph shown on the right. Scale bar, 5 µm and 2.5 µm. **c** Proportion of MyD88–IRAK4 colocalized puncta with lifetimes ≤ 30 s or > 30 s for IRAK4-WT versus IRAK4-ΔKD paired with MyD88-WT. Data points represent replicate means. **d** Percentage of total MyD88 puncta stably colocalized with IRAK4-WT versus IRAK4-ΔKD. Violin plots show single-cell distributions with replicate means superimposed. n = 6 independent replicates for both IRAK4-WT and IRAK4-ΔKD in (C) and (D). **e**–**g** Deletion of the IRAK4 kinase domain disrupts IRAK4–IRAK1 assembly. **e** Representative TIRF image of EL4-3xKO cells expressing IRAK4-ΔKD-GFP with IRAK1-mScarlet, with red line indicating the position used for kymograph generation (left panel). The corresponding kymograph shown on the right. Scale bar, 5 µm and 2.5 µm. **f** Proportion of IRAK4–IRAK1 colocalized puncta with lifetimes ≤ 30 s or > 30 s for IRAK4-WT versus IRAK4-ΔKD paired with IRAK1-WT. **g** Percentage of total IRAK4 puncta stably colocalized with IRAK1 for IRAK4-WT versus IRAK4-ΔKD. n = 4 and 3 independent replicates for IRAK4-WT and IRAK4-ΔKD respectively in (F) and (G). **h**–**j** Deletion of the IRAK1 kinase domain does not disrupt IRAK4–IRAK1 assembly. **h** Representative TIRF image of EL4-3xKO cells expressing IRAK4-GFP with IRAK1-ΔKD-mScarlet, with red line indicating the position used for kymograph generation (left panel). The corresponding kymograph shown on the right. Scale bar, 5 µm and 2.5 µm. **i** Proportion of IRAK4–IRAK1 colocalized puncta with lifetimes ≤ 30 s or > 30 s for IRAK4-WT paired with IRAK1-WT versus IRAK1-ΔKD. n = 4 independent replicates for both IRAK1-WT and IRAK4-ΔKD in (I) and (J). **j** Percentage of total IRAK4 puncta stably colocalized with IRAK1-WT versus IRAK1-ΔKD. Violin plots show single-cell distributions with replicate means superimposed with each independent replicates consists of 2–23 cells per replicate. Statistical significance was assessed using the two-sided Wilcoxon rank-sum test in panels D, G, and J. Bars in (**c**, **d**, **f**, **g**, **i** and **j**) represent mean ± SEM.

Myddosome assembly follows an ordered process initiated by MyD88 oligomerization, which serves as a nucleation point for the sequential recruitment of IRAK4 and IRAK1^11^. The kinase activity of IRAK4 is important for propagating downstream signaling responses^12–14^, and co-immunoprecipitation studies have revealed that Myddosome formation is not exclusively governed by DD interactions^10^. These findings implicate the kinase domain and catalytic activity of IRAK4 as critical regulators of complex assembly and maturation. Structural studies of Myddosomes have solely examined DD assemblies^5–7^ in the absence of the IRAK4/1 kinase domains or activity. Here, to delineate the structural mechanisms that modulate DD oligomerization and signaling, we used AlphaFold3 (AF3)^15^ to generate a structural model of the mouse Myddosome using full length proteins that includes the IRAK4/1 kinase domains (Fig. 1a). The predicted complex revealed not only a mixed oligomer of DDs, but also an interaction interface between the IRAK4 and IRAK1 kinase domains (Fig. 1a). This predicted structural model suggests that IRAK kinase domain interactions and possibly activity could regulate the controlled assembly and maturation of the Myddosome.

Based on our structural model and previous analysis^10^, we expected that the IRAK4/1 kinase domains would be essential for Myddosome assembly and maturation. To delineate the exact role and timing of kinase domain activity in Myddosome assembly, we analyzed the dynamics of MyD88:IRAK4 and IRAK4:IRAK1 heterotypic interactions in live cells. By reconstituting knockout cell lines with IRAK4/1 truncations lacking the kinase domain and tagged with fluorescent proteins (referred to as IRAK4-ΔKD and IRAK1-ΔKD), we used an established two-color high-resolution microscopy assay^11^ to analyze the discrete stages of Myddosome assembly. This experimental approach allowed us to directly assay the co-assembly and stability of these two heterotypic DD interactions, in the presence or absence of the IRAK4 kinase domain, with single-complex resolution.

Examining the first DD interaction in Myddosome assembly, we found that co-assembly of MyD88:IRAK4 was not impacted by the loss of the IRAK4 kinase domain since both full-length and IRAK4--ΔKD co-assembled with MyD88. Furthermore, IRAK-ΔKD and WT co-assemblies with MyD88 had equivalent size and lifetime distributions (Fig. 1b–d, and Supplementary Fig.1a through e). However, signaling was only restored with full-length IRAK4 (Supplementary Fig. 1f). This is consistent with the IRAK4 kinase domain being critical for signal transduction. Therefore, the initial steps of signal transduction and Myddosome formation are solely regulated by DD assembly. We conclude that the heterotypic interactions between the MyD88 and IRAK4 DDs are sufficient to form a stable complex and do not require the IRAK4 kinase domain. However, DD heterotypic assembly alone is insufficient to transduce signaling.

One possible explanation for the lack of signal transduction with the IRAK4-ΔKD could be a failure to recruit IRAK1^10^. Therefore, we next examined the heterotypic DD interactions between IRAK4 and IRAK1. We created EL4 cells with a triple knock out of MyD88, IRAK4 and IRAK1 Myddsome components (referred to as 3xKO cells, Supplementary Fig. 1g) and reconstituted them with IRAK4-WT-GFP or IRAK4-ΔKD and IRAK1-mScarlet. We found that the IRAK4 consisting solely of the DD (IRAK4-ΔKD) was unable to recruit and incorporate IRAK1 into nascent Myddosome complexes (Fig. 1e–g and Supplementary Movie S1). The expression of IRAK4-ΔKD resulted in a greater than 6-fold reduction of stable IRAK1 co-assembly (5.36% and 0.80% for IRAK4 WT and IRAK4-ΔKD, respectively; stable colocalization was defined as an association greater than 30 s; Fig. 1g). We conclude that the IRAK4 kinase domain is required to recruit IRAK1, thereby leading to Myddosome maturation and downstream signal transduction.

Based on these results and our structural model (Fig. 1a, e–g), we hypothesized that the interaction between the kinase domains of IRAK4 and IRAK1 stabilizes the heterotypic DD interaction between these proteins. Therefore, IRAK1 lacking the kinase domain (IRAK1-ΔKD) would be expected to phenocopy the expression of IRAK4-ΔKD. Surprisingly, we observed stable incorporation of IRAK1-ΔKD into assembling Myddosomes (Fig. 1h–j, Supplementary Fig. 1h and Supplementary Movie S2). Thus, while the interaction between the IRAK4:IRAK1 DDs is intrinsically stable, the IRAK1 kinase domain possibly auto-inhibits its DD and prevents its incorporation. This would suggest that the interaction with the IRAK4 kinase domain is required to release IRAK1 autoinhibition and allow stable assembly into Myddosomes and downstream signaling.

### IRAK4 kinase domain regulates the incorporation of IRAK2/3 into Myddosomes

**Fig. 2:**
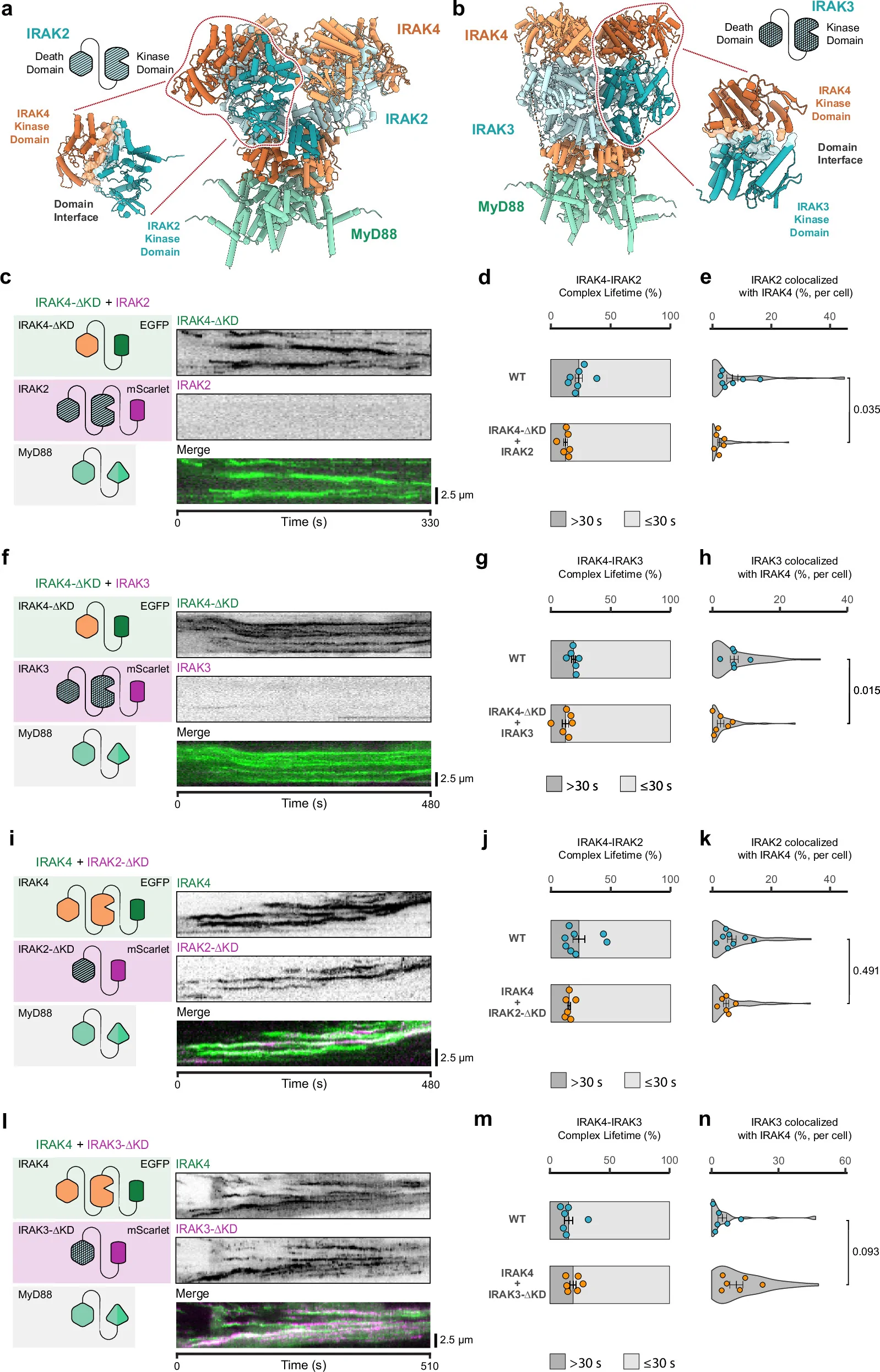
The IRAK4 kinase domain regulates IRAK2/3 incorporation into Myddosome complexes. **a**, **b** AF3-predicted models of Myddosome complexes containing the DDs and kinase domains of IRAK4 with IRAK2 (**a**) or IRAK3 (**b**). Schematic domain organization of MyD88, IRAK4, and IRAK2/3 and AF3-predicted model with details of the putative interaction interface between IRAK4 and IRAK2/3 kinase domains (outlined in dashed red). The IRAK4/2 and IRAK4/3 kinase domain heterodimer interface is highlighted. **c**–**h** Deletion of the IRAK4 kinase domain disrupts IRAK4–IRAK2 (**c**–**e**) and IRAK4–IRAK3 (**f**–**h**) co-assembly. **c**, **f** Representative kymographs from TIRF microscopy of EL4-3xKO cells expressing IRAK4-ΔKD-GFP with IRAK2-mScarlet (**c**) or IRAK3-mScarlet (**f**) activated on IL-1–functionalized supported lipid bilayers. Scale bars, 2.5 µm. **d**, **g** Proportion of colocalized IRAK4–IRAK2 (**d**) or IRAK4–IRAK3 (**g**) puncta with lifetimes ≤ 30 s or > 30 s for IRAK4-WT versus IRAK4-ΔKD paired with IRAK2/3-WT. Data points represent replicate means. **e**, **h** Percentage of total IRAK4 puncta stably colocalized with IRAK2 (E) or IRAK3 (H). Violin plots show single-cell distributions with replicate means superimposed. n = 6 and 7 independent replicates for IRAK4-WT versus IRAK4-ΔKD with IRAK2 respectively in (D) and (E). n = 6 independent replicates for both IRAK4-WT versus IRAK4-ΔKD with IRAK3 in (G) and (H). **i**–**n** Deletion of the IRAK2 or IRAK3 kinase domain does not disrupt IRAK4–IRAK2 or IRAK4–IRAK3 co-assembly. **i**, **l** Representative kymographs from TIRF microscopy of EL4-3xKO cells expressing IRAK4-GFP with IRAK2-ΔKD-mScarlet (**i**) or IRAK3-ΔKD-mScarlet (**l**). Scale bars, 2.5 µm. **J**, **m** Proportion of colocalized IRAK4–IRAK2 (**J**) or IRAK4–IRAK3 (**m**) puncta with lifetimes ≤ 30 s or > 30 s for IRAK4-WT paired with IRAK2/3-ΔKD. **k**, **n** Percentage of IRAK4 puncta stably colocalized with IRAK2-WT versus IRAK2-ΔKD (**k**) or IRAK3-WT versus IRAK3-ΔKD (**n**). n = 8 and 6 independent replicates for IRAK2-WT versus IRAK2-ΔKD with IRAK4 respectively in (J) and (K). n = 6 independent replicates for both IRAK3-WT versus IRAK3-ΔKD with IRAK4 in (G) and (H). Violin plots show single-cell distributions with replicate means superimposed with 3-25 cells per replicate. Statistical significance was assessed using the two-tailed Wilcoxon rank-sum test in panels (**e**, **h**, **k**, **n**). Bars in (**d**, **e**, **g**, **h**, **j**, **k**, **m**, **n**) represent mean ± SEM.

To determine whether the role of the IRAK4 kinase domain is to relieve autoinhibition in other IRAK family members, we further examined IRAK2 and IRAK3. Similar to IRAK1, IRAK2 and IRAK3 can also co-assemble with MyD88. IRAK2 activates equivalent downstream inflammatory signaling reactions to IRAK1^16^, suggesting functional redundancy. In contrast, IRAK3 is proposed to be a negative regulator of signal transduction^17^. Unlike IRAK1, IRAK2 and IRAK3 are putative pseudokinases^18,19^. Given these biochemical differences, Myddosomes composed of IRAK1, IRAK2, or IRAK3 could have different assembly mechanisms. If the IRAK4 kinase domain acts as a universal regulatory switch activating IRAK DD assembly, it would be expected to control the incorporation of IRAK2 or IRAK3 into Myddosomes. We generated a structural model of the mouse Myddosome composed of either IRAK2 or IRAK3 using AF3 (Fig. 2a, b). Similar to the model with IRAK1, we found a predicted binding interface between the IRAK4 and IRAK2 or IRAK3 kinase domains. This supports the hypothesis that the IRAK4 kinase domain relieves autoinhibition across IRAK paralogs.

From this structural model, we hypothesized that the IRAK4 kinase domain regulates the incorporation of IRAK2 and IRAK3 into forming Myddosome complexes. EL4 cells do not express detectable levels of IRAK2 or IRAK3 (Supplementary Fig. 2a), allowing us to reconstitute EL4-3xKO cells with combinations of full-length and truncated versions of IRAK4, IRAK2, or IRAK3. We found that IRAK2 and IRAK3 are incorporated into Myddosomes with similar kinetics to those of IRAK1 when co-expressed with wild-type IRAK4 (Supplementary Fig. 2b, c). However, when co-expressed with IRAK4-ΔKD, we observed more transient recruitment and reduced stable incorporation of IRAK2 and IRAK3 into Myddosomes (Fig. 2c–h, Supplementa Movie S3). Although the defect was less severe than with IRAK1, IRAK4-ΔKD showed reduced stable association with IRAK2/3 puncta compared to WT IRAK4 (Fig. 2e, h). Additionally, deletion of the IRAK4 kinase domain produced smaller IRAK2/3 oligomers (Supplementary Fig. 2d, e), indicating failed Myddosome maturation. This suggests that the IRAK4 kinase domain regulates the heterotypic assembly of DDs across all IRAK paralogs by possibly relieving intra-molecular inhibition.

If IRAK2/3 are autoinhibited to prevent DD assembly, the removal of the kinase domain would be expected to restore stable heterotypic assembly with the IRAK4 DD. Reconstitution of 3xKO cells with full-length IRAK4 and the IRAK2/3-ΔKD led to stable heterotypic assembly (Fig. 2i–n, Supplementary Fig. 2f and g Supplementary Movie S4). This suggests that the kinase domain of IRAK4 regulates the controlled assembly of heterotypic DDs by relieving internal inhibition of IRAK2/3 exerted by their kinase domains. We conclude that the IRAK4 kinase domain is a regulator of heterotypic DD assembly and controls the final stages of Myddosome maturation by relieving an intramolecular inhibition present within the IRAK1/2/3 paralogs.

### IRAK4 kinase activity is required to stably incorporate IRAK1/2/3 into Myddosomes

Our results show that the IRAK4 kinase domain is required for the stable incorporation of IRAK1/2/3 into Myddosomes. Based on the structural model (Figs. 1a, 2a, b) and biochemical studies^14,19^, we predicted that the interaction between the IRAK4 and IRAK1/2/3 kinase domains is regulated by IRAK4 kinase activity. In vitro, only the phosphorylated IRAK4 domain can heterodimerize with IRAK paralog kinase domains^14,19^. IRAK4 kinase domain autophosphorylates, and this occurs downstream of DD co-assembly with MyD88, which triggers homodimerization and autophosphorylation of serine/threonine residues in its kinase domain^20,21^. Consistent with this molecular sequence of Myddosome assembly, IRAK4 that lacks a DD or has a patient-associated mutation within the DD that inhibits oligomerization^22,23^ could not co-assemble with MyD88 or undergo autophosphorylation in response to IL-1 (Supplementary Fig. 3), highlighting the sequential nature of this process. If IRAK4 kinase activity and autophosphorylation are involved in relieving autoinhibition of IRAK paralogs, IRAK4 kinase-inactive mutants should phenocopy the expression of IRAK4-ΔKD and disrupt IRAK1 incorporation (Fig. 1e–g). To test this hypothesis, we assayed a kinase-inactive version of IRAK4 (IRAK4-K213A/K214A) that has previously been shown to be unable to trigger the incorporation of IRAK1 into Myddosomes^10^. This mutation could not restore IL-1 signaling responses in IRAK4 KO cells (Supplementary Fig. 1b), consistent with previous studies on IRAK4 kinase requirements^10^. Importantly, IRAK4-K213A/K214A did not disrupt MyD88 oligomerization or MyD88:IRAK4 co-assembly, confirming that the initial steps of Myddosome formation are solely regulated by DD assembly (Supplementary Fig. 4a–c). Therefore, IRAK4 kinase activity functions as a regulatory checkpoint that specifically regulates signaling events downstream of MyD88:IRAK4 co-assembly and multimerization.

**Fig. 3:**
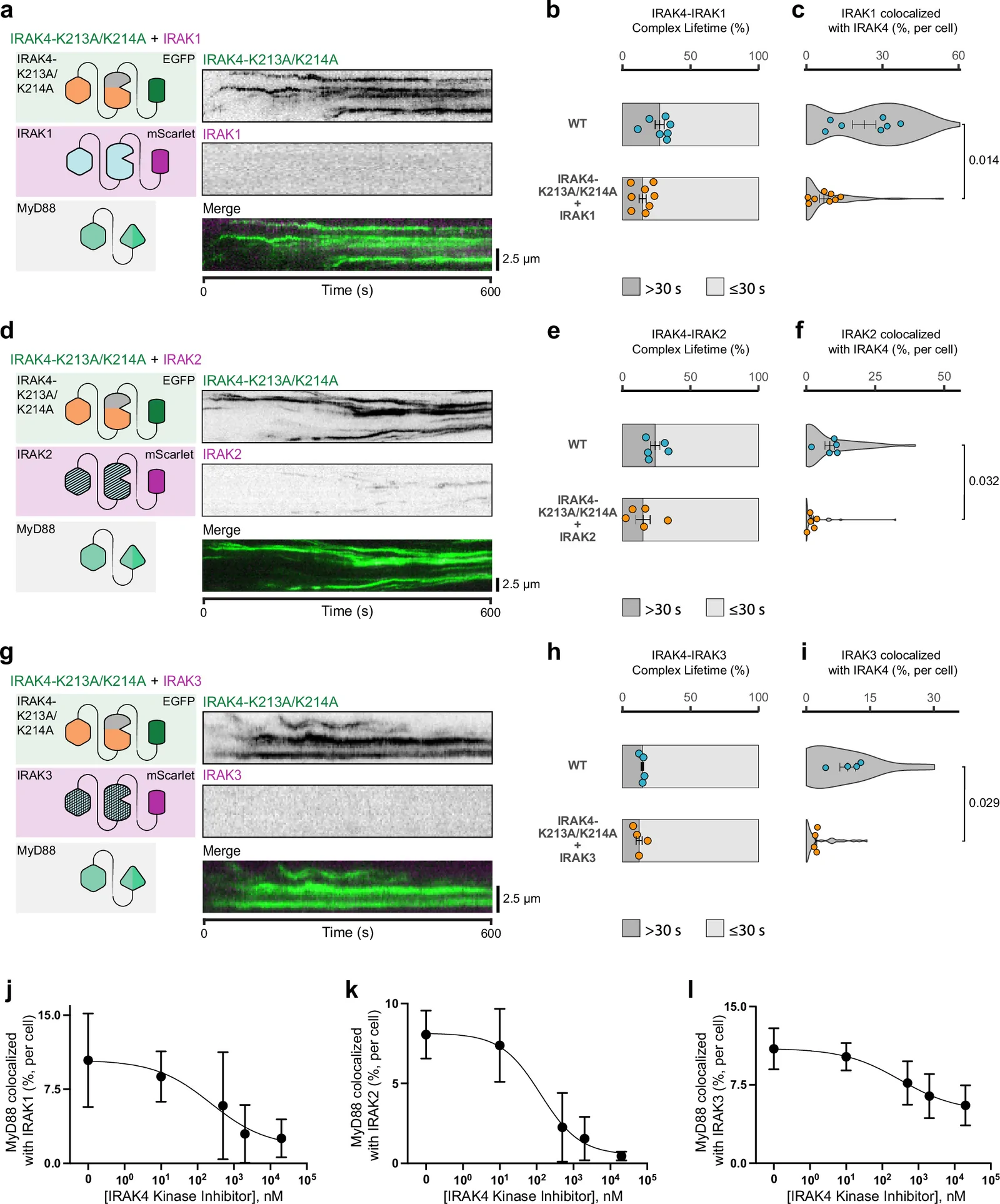
IRAK4 kinase activity is required for stable incorporation of IRAK1/2/3 into Myddosomes. A kinase-inactive IRAK4 mutant (K213A/K214A) fails to stably incorporate IRAK1 (**a**–**c**), IRAK2 (**d**–**f**), or IRAK3 (**g**–**i**) into Myddosomes. **a**, **d**, **g** Representative kymographs from TIRF microscopy of EL4-3xKO cells expressing IRAK4-(K213A/K214A)-GFP with IRAK1-mScarlet (**a**), IRAK2-mScarlet (**d**), or IRAK3-mScarlet (**g**). Scale bars, 2.5 µm. **b**, **e**, **h** Proportion of colocalized IRAK4–IRAK1 (**b**), IRAK4–IRAK2 (**e**), or IRAK4–IRAK3 (**h**) puncta with lifetimes ≤ 30 s or > 30 s for IRAK4-WT versus IRAK4-(K213A/K214A) paired with IRAK1/2/3-WT. Data points represent replicate means. n = 5 independent replicates for both IRAK4-WT versus IRAK4-(K213A/K214A) with IRAK2 in (E). n = 4 independent replicates for both IRAK4-WT versus IRAK4-(K213A/K214A) with IRAK3 in (H). **c**, **f**, **i** Percentage of total IRAK4 puncta stably colocalized with IRAK1 (**c**), IRAK2 (**f**), or IRAK3 (**i**) for IRAK4-WT versus IRAK4-(K213A/K214A) paired with IRAK1/2/3-WT. Violin plots show single-cell distributions with replicate means superimposed. n = 5 independent replicates for both IRAK4-WT versus IRAK4-(K213A/K214A) with IRAK2 in (F). n = 4 independent replicates for both IRAK4-WT versus IRAK4-(K213A/K214A) with IRAK3 in (I). **j**–**l** Stable colocalization of MyD88 puncta with IRAK1 (**j**), IRAK2 (**k**), or IRAK3 (**l**) in cells treated with varying doses of IRAK4 kinase inhibitor. Each independent replicates for each allelic pairing consists of 3-43 cells per replicate. Statistical significance was assessed using the two-tailed Wilcoxon rank-sum test in panels (**c**, **f**, **i**). Bars represent mean ± SEM in panels (**b**, **c**, **e**, **f**, **h**, and **i**), and mean ± SD in panels (**j**–**m**).

To determine whether IRAK4 kinase activity and autophosphorylation relieves IRAK1 autoinhibition and permits IRAK4:IRAK1 DD coassembly, we directly imaged co-assembly of IRAK1 with WT and kinase-inactive IRAK4. We found that IRAK4-K213A/K214A was unable to recruit and incorporate IRAK1 into nascent Myddosome complexes (Fig. 3a–c and Supplementary Movie S5). Similar to IRAK4-ΔKD, this kinase-inactive mutant had a 3-fold reduction in stable colocalization with IRAK1 (Fig. 3c). Thus, IRAK4 kinase activity and autophosphorylation control an essential regulatory switch that ensures IRAK1 incorporation and DD oligomerization into nascent Myddosomes. This is consistent with IRAK4 autophosphorylation functioning as a switch which overrides IRAK1 autoinhibition.

To determine whether IRAK4 autophosphorylation functions as a universal regulatory switch in Myddosome maturation, regardless of the final composition, we tested IRAK2/3 incorporation. We found that IRAK4-K213A/K214A only recruited IRAK2 transiently compared to WT, and very few IRAK4-K213A/K214A assemblies formed stable complexes with IRAK2 (Fig. 3d–f). In comparison, IRAK3 had a similar lifetime distribution of co-assembly between the full-length and kinase-dead versions of IRAK4 (Fig. 3g, h). However, there was a 4-fold reduction in the frequency of IRAK3 assemblies that were stably associated with IRAK4 (Fig. 3i). We conclude that IRAK4 kinase activity and autophosphorylation regulate IRAK1/2/3 incorporation and DD oligomerization in Myddosomes. Therefore, IRAK4 autophosphorylation is a regulatory switch that controls the final assembly stage of Myddosome assembly, regardless of the final composition.

If IRAK4 autophosphorylation functions as a regulatory switch acting on IRAK1/2/3, then IRAK4 kinase inhibitors would be expected to phenocopy kinase-inactive IRAK4 expression and block IRAK1/2/3 recruitment. This effect would also be titratable, such that higher concentrations of the inhibitor would produce more pronounced inhibition of IRAK1/2/3 recruitment and colocalization with Myddosomes. We confirmed that a specific IRAK4 inhibitor^24^ blocked autophosphorylation, had no effect on MyD88:IRAK4 coassembly, inhibited downstream signaling, and, like IRAK4-K213/214A^10^, inhibited IRAK1 co-immunoprecipitation with MyD88 (Supplementary Fig. 4d to g). Consistent with our observations in kinase-inactive mutants, the inhibitor also had a pronounced and titratable effect on IRAK1 stability and incorporation into Myddosomes (Fig. 3j). We found similar effects on IRAK2 and IRAK3 (Fig. 3k, l). Therefore, we conclude that IRAK4 autophosphorylation functions as a regulatory switch to control Myddosome maturation, possibly by relieving IRAK1/2/3 autoinhibition and thereby allowing the DDs to co-assemble and oligomerize.

### IRAK4 autophosphorylation and heterodimerization of IRAK1:IRAK4 kinase domains are discrete steps in Myddosome assembly

**Fig. 4:**
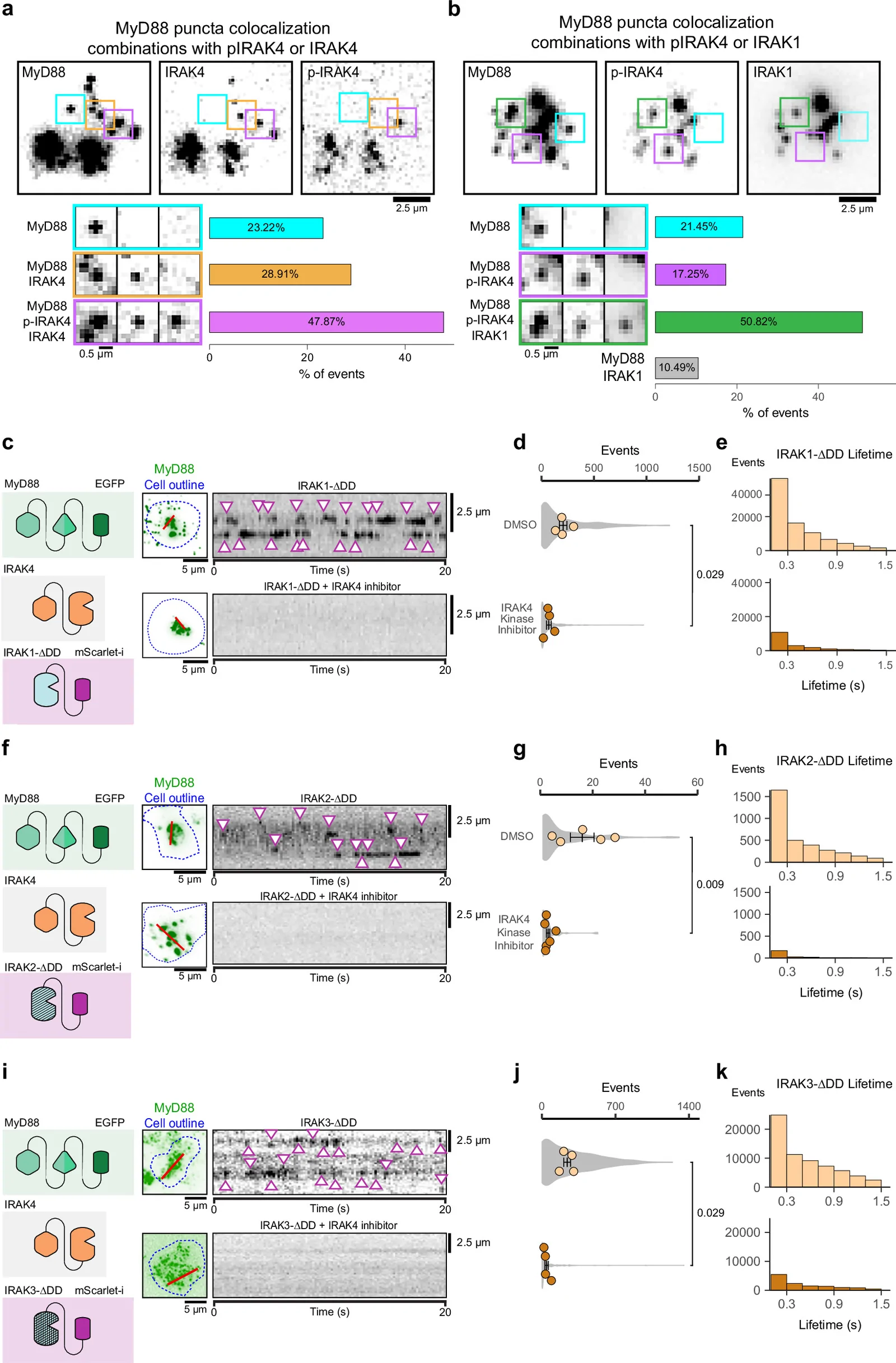
IRAK4 autophosphorylation functions as a regulatory switch for kinase domain–mediated recruitment of IRAK1 and IRAK2. **a**, **b** TIRF images of EL4-MyD88-GFP/IRAK4-mScarlet (**a**) or EL4-MyD88-GFP/IRAK1-mScarlet (**b**) cells stained for pIRAK4 after activation on IL-1β–functionalized SLBs. Boxes highlight assemblies containing MyD88 alone (cyan), MyD88 + IRAK4 (yellow–orange), or MyD88 + IRAK4 + pIRAK4 (magenta). Bar plots show the percentage of MyD88 puncta colocalizing with IRAK4 or pIRAK4 (**a**) and the fraction of pIRAK4 puncta colocalizing with IRAK1 (**b**). Insets show boxed regions across channels. Data from 422 (**a**) and 429 (**b**) puncta. Scale bars, 2.5 µm (overview) and 0.5 µm (insets). **c**–**e** Single-molecule TIRF imaging of EL4-MyD88-GFP/IRAK1-KO cells expressing IRAK1-ΔDD-mScarlet. **c** MyD88-GFP reference image and representative kymographs from the IRAK1-ΔDD-mScarlet channel. The red line in the MyD88-GFP image indicates the position used to generate the kymograph in the mScarlet channel. Scale bars, 5 µm and 2.5 µm. **d** Violin plots quantifying recruitment events per cell for DMSO versus IRAK4 inhibitor–treated cells (mean ± SEM; *n* = 4 independent replicates, 15–297 cells per replicate). **e** Histogram of IRAK1-ΔDD dwell times with or without inhibitor. **f**–**h** Equivalent single-molecule TIRF imaging experiments with IRAK2-ΔDD-mScarlet. **f** MyD88-GFP reference image and representative kymographs. Scale bars, 5 µm and 2.5 µm. **g** Recruitment quantified as in (**d**) (mean ± SEM; *n* = 5 and 6 independent replicates for for IRAK2-ΔDD with DMSO or IRAK4 kinase inhibitor respectively, 3-93 cells per replicate). **h** Histogram of IRAK2-ΔDD dwell times. **i**–**k** Equivalent single-molecule TIRF imaging experiments with IRAK3-ΔDD-mScarlet. **i** MyD88-GFP reference image and representative kymographs. Scale bars, 5 µm and 2.5 µm. **j** Recruitment quantified as in (**d**) (mean ± SEM; *n* = 4  independent replicates, 54–120 cells per replicate). **k** Histogram of IRAK3-ΔDD dwell times. Arrowheads in kymographs (**c**, **f**, **i**) highlight transient recruitment events of IRAK1/2-ΔDD-mScarlet. Statistical significance was assessed using two-sided Wilcoxon rank-sum test in panels D, G, J.

To understand how IRAK4 autophosphorylation serves as a molecular switch in Myddosome assembly, we examined the precise temporal relationship between IRAK4 phosphorylation and Myddosome maturation. Consistent with IRAK4 phosphorylation regulating heterotypic DD assembly, phosphorylated IRAK4 (pIRAK) levels increase during the first 60 minutes post IL-1 stimulation (Supplementary Fig. 5a, b), indicating that Myddosome assembly and IRAK4 phosphorylation occur contemporaneously. To understand this dynamic process at the level of single complexes, we exploited the asynchronous assembly of Myddosomes in individual cells (Supplementary Fig. 5c, d) and chemical fixation as a way to observe various assembly intermediates and reconstruct the timeline of assembly and IRAK1 incorporation. Chemical fixation of IL-1-stimulated cells expressing fluorescently tagged Myddosome components and stained with pIRAK4-specific antibodies revealed MyD88 puncta with IRAK4-mScarlet both with and without pIRAK4 (Fig. 4a), whereas MyD88-GFP/IRAK1-mScarlet cells showed puncta containing MyD88 alone, MyD88 with pIRAK4, or MyD88 with both pIRAK4 and IRAK1 (Fig. 4b). Critically, over 80% of MyD88 puncta containing IRAK1 also contained pIRAK4. The relative proportions of these assembly states (Fig. 4a, b) enabled us to reconstruct the Myddosome assembly sequence: MyD88 oligomerization initiates the process, followed by DD-dependent recruitment and oligomerization of unphosphorylated IRAK4, which leads to kinase-domain homodimerization and trans-autophosphorylation, homodimer disassembly, and finally heterodimerization of the phosphorylated IRAK4 kinase domain with the IRAK1 kinase domain, overcoming IRAK1’s autoinhibition. This reconstructed temporal order of assembly is consistent with the integral role of IRAK4 kinase activity in Myddosome signaling. Furthermore, the stepwise assembly sequence suggests that the discrete step of IRAK4 autophosphorylation is a molecular switch that must be activated to trigger stable IRAK1 incorporation.

A key prediction of our sequential model of Myddosome assembly is that heterodimerization of the IRAK4 and IRAK kinase domains precedes DD oligomerization. Therefore, we would expect the IRAK kinase domain alone to be recruited to nascent Myddosomes independently of the DD, in a manner dependent on IRAK4 autophosphorylation. To test this, we expressed IRAK1, IRAK2 and IRAK3 truncation mutants containing only the kinase domain (IRAK1/2/3-ΔDD) fused to mScarlet in MyD88-GFP/IRAK1 KO cells and assayed recruitment to Myddosomes using single-molecule microscopy. MyD88 puncta recruited IRAK1/2/3-ΔDD (Fig. 4c, f, i and Supplementary Movie S6). These recruitment events were typically single molecule, transient, and dependent on IRAK4 kinase activity, as their frequency was reduced in the presence of an IRAK4 kinase inhibitor (Fig. 4d, e, g, h, j, k). In conclusion, these data show that the kinase domains of IRAK1/2/3 are sufficient for recruitment to assembling Myddosomes and that this interaction requires IRAK4 kinase activity. Taken together, these findings demonstrate that IRAK4 kinase activity regulates DD assembly by enforcing a sequential order of interactions. In this way, IRAK4 autophosphorylation acts as a switch that directs the timely incorporation of IRAKs and ensures that Myddosome maturation proceeds in a controlled and directional manner.

### A IRAK4 phosphomimetic mutant restores IRAK1 heterodimerization and Myddosome incorporation independent of kinase activity

**Fig. 5:**
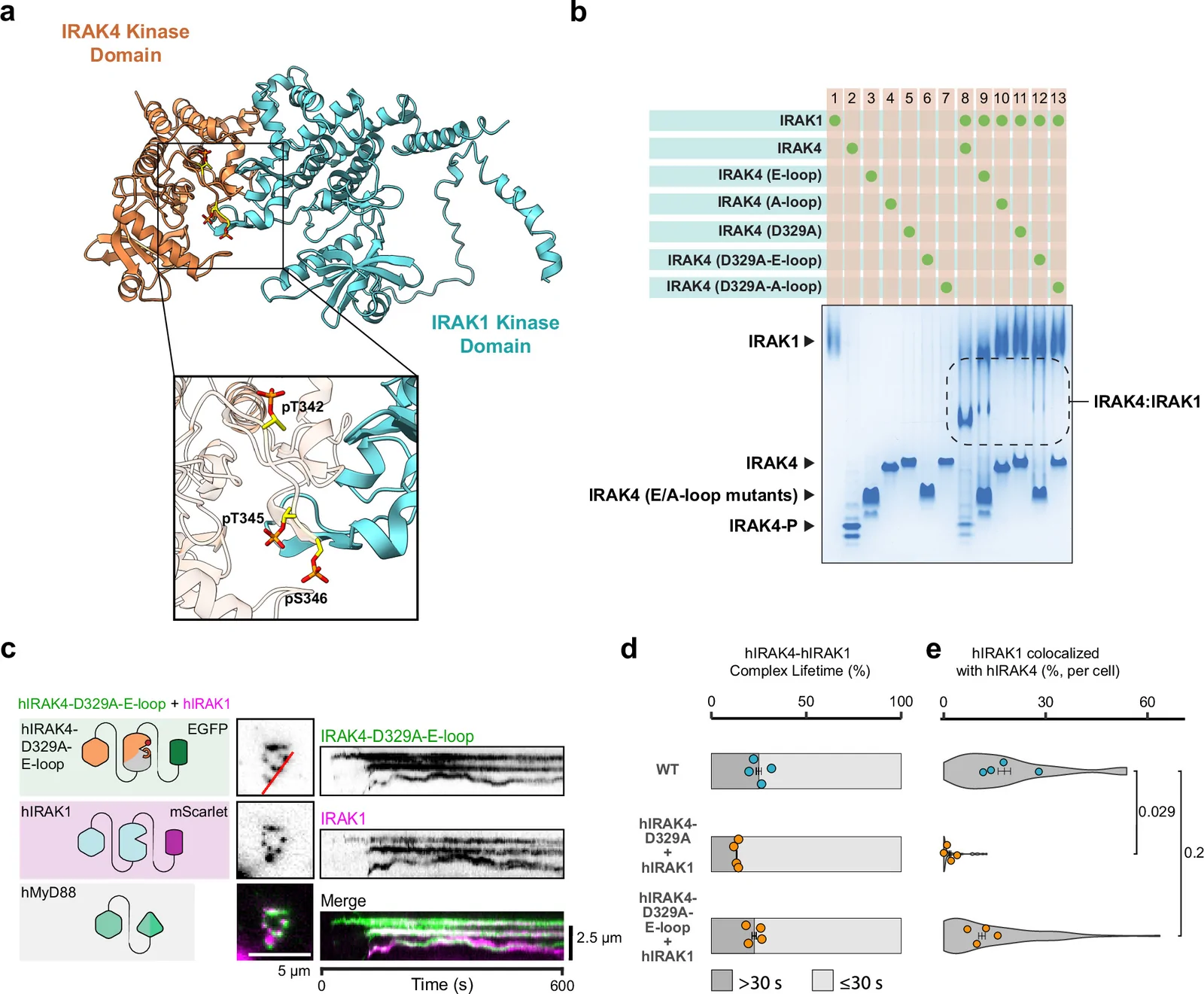
A IRAK4 phosphomimetic mutant restores heterodimerization with the IRAK1 kinase domain and Myddosome maturation. **a** A structural model of the human IRAK4:IRAK1 kinase domain heterodimer was generated with AF3. This model predicts that the phosphorylated serine and threonine residues of IRAK4 within the activation loop are at the heterodimer interface. **b** Native PAGE co-migration analysis of the phosphorylated, unphosphorylated, kinase inactive (D329A) and activation loop mutants (A-loop or phosphomimetic E-loop) of the human IRAK4 kinase domains with the kinase domains of human IRAK1 was performed. The gel was stained with Coomassie Blue. Inputs are indicated above the numbered lanes, and the migration position of co-complexes of IRAK4 kinase domain allelic variants with the IRAK1 kinase domain is highlighted with a dotted line. **c** Representative kymograph from TIRF microscopy of EL4-3xKO cells expressing IRAK4-(D329A/E-loop)-GFP with IRAK1-mScarlet. Scale bars, 2.5 µm. **d** Proportion of colocalized IRAK4–IRAK1 puncta with lifetimes ≤ 30 s or > 30 s for human IRAK4-WT versus IRAK4-(D329A) or IRAK4-(D329A/E-loop) paired with IRAK1-WT. Data points represent replicate means. **e** Percentage of total IRAK4 puncta stably colocalized with human IRAK4-WT versus IRAK4-(D329A) or IRAK4-(D329A/E-loop) paired with IRAK1-WT. Violin plots show single-cell distributions with replicate means superimposed. Data represent *n* = 4 independent replicates for each allelic pairing, with 4–28 cells per replicate. Bars in (**d**, **e**) represent mean ± SEM. Statistical significance was assessed using the two-tailed Wilcoxon rank-sum test in panel (**e**).

To understand the biochemical nature of this IRAK4 autophosphorylation-mediated switch further, we examined how IRAK4 autophosphorylation contributes to heterodimerization with the IRAK1 kinase domain. The AF3 structural model of the human IRAK4 and IRAK1 kinase domain heterodimer predicts that the phosphorylated residues of IRAK4, in a region referred to as the activation loop^14,20,25,26^, are situated at the dimer interface, suggesting a role in regulating this interaction (Fig. 5a). In vitro, we confirmed that only the phosphorylated human IRAK4 kinase domain heterodimerizes with the IRAK1 kinase domain and consequently requires kinase activity (Fig. 5b), which is consistent with previous biochemical analysis^14,19,27^. We reasoned that if a kinase-inactive IRAK4 were combined with rational mutation of the IRAK4 activation loop to mimic the negative charge of phosphorylation, this would induce equivalent conformational changes and an electrostatic environment that could restore heterodimerization with IRAK1, independent of IRAK4 kinase activity. We mutated the threonine/serine residues within this activation loop to glutamic acid (referred to as E-loop mutant), and tested whether it could restore heterodimerization with IRAK1 in vitro by native PAGE (Fig. 5b). We found that this mutation could even partially restore heterodimerization when combined with a kinase-inactive mutant. We then tested whether this triple mutation restored IRAK1 recruitment and assembly in cells and found that it restored IRAK1 recruitment to an IRAK4 kinase-inactive mutant (Fig. 5c–e and Supplementary Movie S7). Mutating the activation loop to alanine (A-loop) in a kinase-active IRAK4 also reduced IRAK1 oligomerization and incorporation into Myddosomes (Supplementary Fig. 6c through f). As these mutations were analyzed in human alleles of IRAK4 and IRAK1, we tested the equivalent activation loop mutations in mouse alleles and found that E-loop phosphomimetic mutations could restore IRAK1 recruitment to a kinase-inactive IRAK4 allele (Supplementary Fig. 6g through l). These data show that autophosphorylation facilitates the confirmation change in IRAK4 that leads to heterodimerization with the IRAK1 kinase domain. Our results thus provide a molecular mechanism that explains how IRAK4 autophosphorylation functions as a molecular switch to control directional DD assembly and Myddosome maturation.

## Discussion

**Fig. 6:**
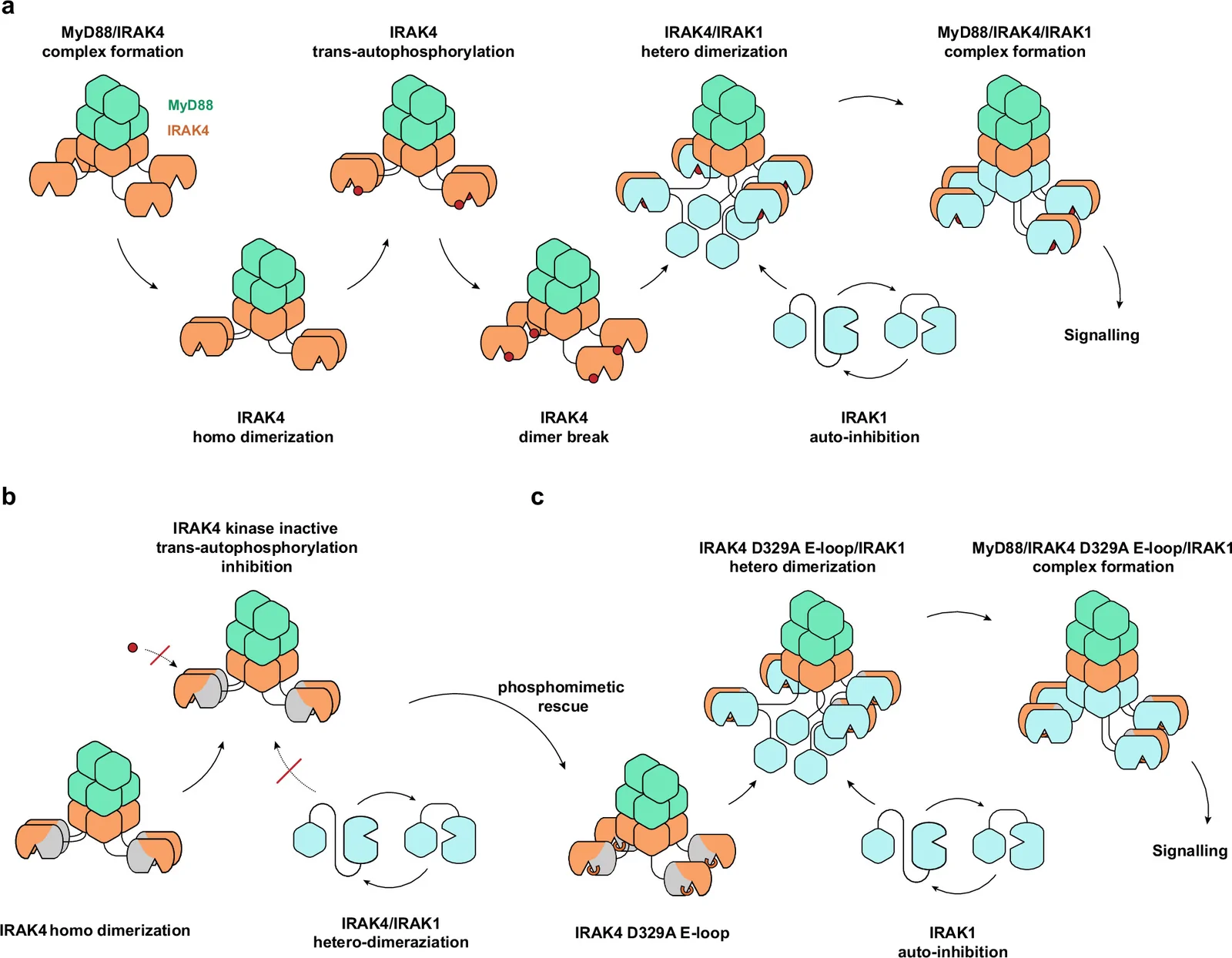
Molecular model for the timeline of Myddosome assembly and maturation. **a** Conceptual model of IRAK4 kinase activity in Myddosome maturation. After the heterotypic assembly of MyD88 and IRAK4 death domains (DDs), the IRAK4 kinase domains homodimerize and undergo autophosphorylation. This autophosphorylation functions as a molecular switch, enabling the phosphorylated IRAK4 kinase domain to heterodimerize with the IRAK1 kinase domain. Heterodimerization stabilizes IRAK1 in a conformation that permits subsequent incorporation of the IRAK1 DD into the MyD88–IRAK4 DD oligomer. **b**, **c** Inhibition of IRAK4 autophosphorylation, either by pharmacological inhibitors or expression of kinase-dead alleles, arrests Myddosome assembly at the MyD88–IRAK4 stage (**b**). This block can be bypassed by expression of a phosphomimetic IRAK4 mutant (**c**), which, even in a kinase-inactive context, supports IRAK1 recruitment and incorporation into the Myddosome.

Here, we demonstrate that IRAK4 kinase-domain autophosphorylation functions as a molecular switch that controls the final stages of Myddosome maturation in IL-1 signal transduction. Using a combination of high spatial-temporal resolution microscopy and molecular analysis, we reconstructed the temporal order of Myddosome assembly (Fig. 6). This assembly sequence demonstrates that IRAK4 autophosphorylation is a distinct step that occurs after MyD88:IRAK4 assembly but before the incorporation of IRAK1, 2 and 3 (Figs. 1a, e–g, 2a–h, Supplementary Fig. 2d, e and Figs. 3a–i, 4). This step is critical because it serves to relieve the auto-inhibitory conformation of IRAK1, 2 and 3, and allows for the DD of these paralogs to co-assemble on the platform of the IRAK4 DD oligomers co-assembled with MyD88. Thus, IRAK4 autophosphorylation is an energy-dependent biochemical switch that controls the DD assembly multimerization and heteromeric assembly. This mechanism explains why IRAK4 kinase-inactive mutants or kinase inhibitors can suppress Myddosome inflammatory signaling^10,12–14,21,28,29^. In summary, our data demonstrate how the assembly of a DD signaling complex is not solely controlled by mass-action oligomerization. Instead, DD assembly can integrate a powerful regulatory switch using an energy-dependent phosphorylation mechanism to control signal transduction.

Intramolecular inhibition is a widespread protein control mechanism that enables precise temporal and spatial activation of biochemical pathways. Typically, this regulation relies on weak internal interactions that maintain a dynamic equilibrium between inhibited and activatable states. For example, actin polymerization by N-WASP is spatially controlled by autoinhibition of the VCA domain, mediated by weak (10–100 μM) interactions with domains that also sense upstream inputs such as phosphoinositides and activated Cdc42^30^. This arrangement suppresses spurious activation while remaining highly responsive to bona fide activating signals. Our data suggest that IRAK1/2/3 employ an analogous principle: weak affinity interactions between their DD and kinase domain create a dynamic autoinhibited state. Phospho-IRAK4 functions as an energy-dependent switch that competes for the IRAK1/2/3 kinase domain, releasing the DD to join the assembling Myddosome platform. Because IRAK4 autophosphorylation only occurs when co-assembled with MyD88^14^, this switch is not a simple on–off toggle but a regulatory checkpoint that ensures IRAK activation occurs only in the context of receptor engagement and is spatially restricted to assembling Myddosomes. In doing so, IRAK4 autophosphorylation links upstream receptor inputs to Myddosome maturation, enforcing directionality and proportionate responsiveness in downstream signaling, features that are especially critical for ensuring that IL-1–induced inflammatory outputs remain tightly coupled to receptor activation. In summary, weak and reversible IRAK1/2/3 intramolecular interactions are strong enough to prevent inappropriate activation, yet labile enough to permit rapid and coordinated transitions into active complexes once the correct stimulus is present. In doing so, they set a dynamic threshold that is responsive to the phospho-IRAK4 switch and enforces directionality over DD assembly and downstream signaling.

Beyond IL-1R signaling, multiple inflammatory signaling pathways transduce signals through oligomeric complexes composed of DDs^1,2,4^. Our data shows that multimerization of DDs can be controlled by other biochemical processes, such as post-translational modification, beyond the specific interactions between these domains. The integration of autophosphorylation and DD oligomerization stages is possibly conserved outside of mammals, as Toll receptor activation triggers autophosphorylation of the Drosophila IRAK homolog Pelle kinase^31^. Similar to IRAKs, we find enzymatically active signaling proteins that contain DDs and assemble into multimeric complexes, for example, receptor-interacting protein kinases (RIPK)^32–34^. This suggests a broader prevalence of enzymatic activity controlling protein oligomerization and directionality in signaling cascades. Our insight into the hierarchical interplay between macromolecular assembly and enzymatic activity explains why IRAK4 kinase inhibitors can disrupt controlled Myddosome signal transduction^28,35^, which may also point to a possible broader therapeutic strategy of targeting enzymatic activity to disrupt macromolecular assembly and consequently signal transduction.

## Methods

Details of all materials and resources are given in Supplementary Table 1.

### Cell culture

EL4.NOB1 WT (ECACC, and referred to as EL4 in the paper) and gene-edited cells were grown in RPMI (Thermo Fisher Scientific) with 10% FBS (Sigma) supplemented with 2 mM L-glutamine. EL4 cultures were maintained at a cell density of 0.1-0.5 × 10^6^ cells/ml in 5% CO2, 37 °C. HEK-293T cells (Clontech) were grown in DMEM (Thermo Fisher Scientific) with 10% FBS supplemented and 2 mM L-glutamine. All cells were determined to be negative for mycoplasma using the MycoAlert detection kit (Lonza).

### Generation of CRISPR/Cas9 triple MyD88/IRAK1/IRAK4 knockout Cell Lines

We purchased WT EL4.NOB1 cells that had been electroporated with Cas9 RNPs loaded with sgRNA targeting the MyD88 (gRNA sequence 5’-AAGCAGUGUCCCACAAACAA-3’, ENSMUST00000035092), IRAK4 (gRNA sequence 5’-UCUGGACCAGUAGAUCCACA-3’, ENSMUST00000074936) and IRAK1 (gRNA sequence 5’-AAACAGGGAGUGGAACCUGG, ENSMUST00000114354) gene locus from the Synthego Corporation (Redwood CA, USA). From this pooled population, we isolated single-cell clones by limited dilution on 96-well plates. We screened and identified MyD88/IRAK1/IRAK4 triple knockout single cell clones by western blot analysis (Supplementary Fig. 1c). Western blot analysis was performed with the following antibodies: rabbit polyclonal anti-IRAK4 (Cell Signaling Technology, #4363), rabbit monoclonal anti-IRAK1 (D51G7, Cell Signaling Technology, #4504), goat polyclonal anti-MyD88 (R&D systems, #AF3109), and Mouse monoclonal anti-β-actin (Cell Signaling Technology, #4970). EL4 MyD88/IRAK4/IRAK1-KO knockout cell lines referred to as EL4-3xKO.

### Western blot analysis of IRAK4 phosphorylation

EL4 wt cells were seeded into 6-well plates at a density of 7.5 × 10^5^ cells/well 24 h before stimulation. On the next day cells were stimulated with 1 ng/ml of recombinant mouse IL-1β (Peprotech) in RPMI for several time points between 1–120 min. To prepare cell lysates, the stimulated and unstimulated cells were briefly washed in PBS to remove IL-1, and then resuspended in 2x SDS-PAGE lysis buffer (Thermo 15493939). Lysates were incubated for 5 mins at 95 C before run on NuPAGE Novex 4–12% Bis-Tris Protein Gels (Thermo). Samples were then analyzed by western blotting. To visualize the pIRAK signal intensity relative to the IRAK4 signal, the blots were developed with Rabbit monoclonal anti-pIRAK4 (Thr(P)−345/Ser(P)−346) (a gift from Vikram Rao, Pfizer^12^), Rabbit polyclonal anti-IRAK4 (Cell Signaling Technology, Cat# 4363) and Mouse monoclonal anti-ß-actin (Cell Signaling Technology, #4970) for normalization. Chemiluminescence detection (Super signal substrate, Pierce #34095) was imaged with the BioRad ChemiDoc MP Imaging System. For each replicate, we ran two gels and WB blots loaded with the identical amount of sample, for each replicate, one gel was blotted for IRAK4 and the other for pIRAK4 (see Supplementary Fig. 4c, d). After IRAK4 or pIRAK4 detection, both blots were stripped and then reprobed with anti-actin to normalize the signal intensity of the respective IRAK4/pIRAK4 signal between the two gels. We performed analysis of the Western blot band intensity and quantified the production of pIRAK4 in ImageJ/Fiji. The intensity of the IRAK4 and pIRAK4 bands was normalized to the actin loading control on each blot for each replicate. The normalized pIRAK4 value was then divided by the normal IRAK4 value to give a relative value of pIRAK4 production at each time point assayed. Data was visualized and plotted in R.

For Western Blot analysis of pIRAK4 levels in IRAK4 kinase inhibitor treated/untreated EL4 cells (Supplementary Fig. 3 d), we incubated EL4 cells with media containing 20 µM of IRAK4 kinase inhibitor (PF-06650833, stock concentration of 10 mM in DMSO) for 4 h. DMSO at a final concentration of 0.2% v/v was added to the control samples. EL4 cells were then stimulated with 10–100 ng/ml of recombinant mouse IL-1β for 30 mins. We prepared cell lysates as described above, and ran them on NuPAGE Novex 4–12% Bis-Tris Protein Gels (Thermo). After membrane transfer, cell lysates were probed with anti-pIRAK4 and developed by chemiluminescence as described above.

### Co-immunoprecipitation of Myddosomes from EL4 Cell Lysates

24 hours before stimulation, 5 × 10^6^ EL4 MyD88-GFP cells were seeded in T-75. The next day, the medium was removed and fresh medium, containing 20 µM IRAK4 kinase inhibitor or 0.2% DMSO only, was added. The cells were incubated with IRAK4 kinase inhibitor or DMSO at 37 °C for 4 h. The cells were stimulated with 100 ng/ml of recombinant mouse IL-1β for 15 mins. Stimulated and unstimulated cells were then washed in ice-cold PBS, spun down and resuspended in lysis buffer (50 mM Tris, 150 mM NaCl, 1% Igepal/ NP40, pH 8.0). Lysis buffer contained phosphatase and protease inhibitiors (PhosSTOP, Roche, Cat# 04906837001 and Protease Inhibitor Mini Tablets, EDTA-free, Pierce, Cat# A32955) at the manufacturer’s recommended concentration. Aliquots of the lysates were stored for downstream analysis (input fraction). Lysates were added to GFP beads (Chromotek: GFP-Trap Magnetic Particles, Cat# M-270) pre-equilibrated in Lysis buffer, and incubated overnight with rotation at 4 °C. The next day, beads were applied to magnetic racks and the supernatant was removed. Next, the beads were washed in the Lysis buffer and eluted in 50 µl of the SDS sample loading buffer. The beads were vortexed and incubated for 5 mins at 95 °C. The eluted and input fractions were then ran on NuPAGE Novex 4–12% Bis-Tris Protein Gels (Thermo) and analyzed by Western Blot using the antibodies and protocols described above (Supplementary Fig. 4f). Full-length Western blots and quantification are given in the Source Data.

### Design of a phosphomimetic IRAK4 allele

The homodimerization of the IRAK4 kinase domain in the unphosphorylated state positions an activation loop region containing threonine and serine residues into the opposing kinase domain’s active site^14^. The resulting auto-phosphorylation of these residues induces a conformational change in the kinase domain and drives the dissolution of the homodimer^14^. As only the phosphorylated IRAK4 kinase domain can interact with the IRAK1 kinase domain, it is most likely these residues that contribute to the heterodimerization interface, thereby allowing IRAK4 autophosphorylation to function as a switch that drives heterodimerization, relieves internal inhibition of IRAK1, and allows its DD to incorporate into the Myddosome complex. To mimic the possibly effect of phosphorylation, and lock IRAK4 in a confirmation that mimics the effect of phosphorylation and can interact with IRAK1 kinase domain, we designed a triple mutant where threonine 343 and 345, serine 346 was exchanged with the negatively charged glutamic acid (T343E/T345E/S346E, referred to as E-loop mutant). This mutation was combined with the D329A mutation that disrupted IRAK4 kinase activity. This mutation was cloned into a lentiviral vector for expression in EL4 cells and the kinase domain into insect cell expression vectors for in vitro analysis (see below). In addition, a triple mutation was also created that mutated these loop residues to alanine (A-loop). These residues were combined with both kinase active and inactive IRAK4 alleles. (Fig. 5 and Supplementary Fig. 6)

### Generation of lentiviral constructs

Full details of the Lentiviral plasmid construction are given below. All full-length constructs were confirmed by Sanger sequencing. The full list of DNA constructs used are given in Supplementary Table 1. The protein sequences of IRAK1/2/3/4 mScarlet/mEGFP fusion constructs are given in Supplementary Table 2.

#### pHR-dSV-IRAK4-K213/214A-mScarlet-i/mEGFP, pHR-dSV-IRAK4-ΔKD-mScarlet-i/mEGFP

To prepare the pHR-dSV-IRAK4-(K213/214 A)-mScarlet-i/mEGFP and pHR-dSV-IRAK4-ΔKD-mScarlet-i/mEGFP constructs, the mouse IRAK4 sequence with two mutations (K213/214 A) and the mouse IRAK4 sequence excluding the kinase domain were ordered as gBlocks (IDT). These gBlocks were fused with a PCR fragment of mScarlet-i (amplified by PCR using the forward 5’-tgctacaagagatgtctgctggaggaagtggaggttctggtggtagtgtg-3’ and reverse 5’-aggtcgactctagagtcgcggcc-3’ primers) or mEGFP (PCR using the forward 5’-tgctacaagagatgtctgctggaggaagtggaggttctggtggtagtgtg-3’ and reverse 5’-ggccgcgactctagagtcgacct-3’ primers), and Mlu1/Not1 digested pHR-dSV lentiviral plasmid using Gibson assembly^36^. IRAK4 constructs were fused in frame to mScarlet-i or mEGFP via a 3xGGS linker.

#### pHR-dSV-IRAK4-mEGFP

Full-length IRAK4 (BC051676) was amplified by PCR from a previously described^11^ pHR-dSV-IRAK4-mScarlet-i construct. mEGFP was amplified using the above-mentioned primers. DNA fragments were assembled with the pHR-dSV lentiviral plasmid digested with Mlu1/Not1 using Gibson Assembly.

#### pHR-dSV-IRAK1-mScarlet-i

Full-length Mouse IRAK1 (NCBI Reference Sequence: NP_001171445.1) was ordered as a codon-optimized gBlock (IDT). The gBlock encoded a complementary 5’ end to pHR-dSV. These DNA fragments was fused to a PCR fragment of mScarlet-i (amplified by PCR using the forward 5’-gggggtagcggaggttctggtggtagtgtgagcaaggg-3’ and reverse 5’-cctgcaggtcgactctagagtcgcggccgctttacttgtacagctcgtccatgcc-3’ primers) and a pHR-dSV lentiviral plasmid digested with Mlu1/Not1 using Gibson Assembly. IRAK1 was fused in frame to mScarlet-i via a 3xGGS linker

#### pHR-dSV-IRAK1-ΔKD-mScarlet-i

A fragment of Mouse IRAK1 (NCBI Reference Sequence: NP_001171445.1) that consisted solely of the DD and excluded the kinase domain was ordered as a codon-optimized gBlock (IDT). The gBlock encoded a complementary 5’ end to pHR-dSV. These DNA fragments was fused to the PCR fragment of mScarlet-i (amplified by PCR using the forward 5’-gggggtagcggaggttctggtggtagtgtgagcaaggg-3’ and reverse 5’-cctgcaggtcgactctagagtcgcggccgctttacttgtacagctcgtccatgcc-3’ primers) and a pHR-dSV lentiviral plasmid digested with Mlu1/Not1 using Gibson Assembly. IRAK1-ΔKD was fused in frame to mScarlet-i via a 3xGGS linker

#### pHR-dSV-IRAK1-ΔDD-mScarlet-i

A fragment of Mouse IRAK1 (NCBI Reference Sequence: NP_001171445.1, amino acids 101-710) that consisted solely of the kinase domain and excluded the DD was ordered as a codon-optimized gBlock (IDT). The gBlock encoded a complementary 5’ end to pHR-dSV. This DNA fragments was fused to the PCR fragment of mScarlet-i (amplified by PCR using the forward 5’-gggggtagcggaggttctggtggtagtgtgagcaaggg-3’ and reverse 5’-cctgcaggtcgactctagagtcgcggccgctttacttgtacagctcgtccatgcc-3’ primers) and a pHR-dSV lentiviral plasmid digested with Mlu1/Not1 using Gibson Assembly. IRAK1-ΔDD was fused in frame to mScarlet-i via a 3xGGS linker

#### pHR-dSV-IRAK2-mScarlet-i, pHR-dSV-IRAK3-mScarlet-i

Full-length Mouse IRAK2 (NCBI Reference Sequence: NP_751893.3) and full-length Mouse IRAK3 (NCBI Reference Sequence: NP_082955.2) were ordered as codon-optimized gBlocks (IDT). The gBlock encoded a complementary 5’ end to pHR-dSV. This DNA fragment was fused to the pHR-dSV-IRAK1-mScarlet-i lentiviral plasmid, digested with Mlu1/BamH1 (to excise the IRAK1 fragment) using Gibson Assembly. IRAK2/3 was fused in frame to mScarlet-i via a 3xGGS linker.

#### pHR-dSV-IRAK2-ΔKD-mScarlet-i, pHR-dSV-IRAK3-ΔKD-mScarlet-i

A codon optimized fragment of Mouse IRAK2 (NCBI Reference Sequence: NP_751893.3, amino acids 1-110) and Mouse IRAK3 (NCBI Reference Sequence: NP_082955.2, amino acids 1-110) that consisted solely of the DDs and excluded the kinase domains were ordered from Twist Biosciences cloned into a pHR-dSV-mScarlet-i lentiviral plasmid. This fragment was fused in frame to mScarlet-i via a 3xGGS linker.

#### pHR-dSV-IRAK2-ΔDD-mScarlet-i

A codon optimized fragment of Mouse IRAK2 (NCBI Reference Sequence: NP_751893.3, amino acids 97-622) that consisted solely of the kinase domains and excluded the DDs were ordered from Twist Biosciences cloned into a pHR-dSV-mScarlet-i lentiviral plasmid. This fragment was fused in frame to mScarlet-i via a 3xGGS linker.

#### pHR-dSV40-MyD88-3xFLAG-IRES-Puro, pHR-dSV40-MyD88-3xFLAG-IRES-TagBFP

Mouse or Human MyD88 fused to 3xFLAG-IRES-Puro or 3xFLAG-IRES-TagBFP was ordered as a codon optimized gBlock (IDT). The gBlock encoded a complementary 5’ end to pHR-dSV. This DNA fragment was fused to the pHR-dSV-IRAK1-mScarlet-i lentiviral plasmid digested with Mlu1/Not1 (to excise the IRAK1 mScarlet-i fragment) using Gibson Assembly.

#### pHR-dSV40-hIRAK4-D329A-E-loop-mEGFP, pHR-dSV40-hIRAK4-A-loop-mEGFP

Based on structural data and biochemical characterization of IRAK4 autophosphorylation^14^, we engineered kinase-inactive mutants of IRAK4 that were still capable of interacting with the IRAK1 kinase domain. IRAK4 undergoes autophosphorylation at the serine and threonine-rich region referred to as the activation loop. Analysis of the AF3 model of the fully assembled Myddosome (Fig. 1a) indicates that the IRAK4 active loop region is located at the interface between IRAK4 and IRAK1. To replicate the charged environment induced by phosphorylation in this loop, we substituted S342, T344, and T345 with glutamic acid, creating the E-loop mutant, and combined these mutations with the D329A mutation, which inactivates the kinase domain^12^. Furthermore, we developed an additional mutant by altering the serine/threonine residues within this loop region to alanine, termed the A-loop mutant. These mutants were synthesized as gene fragments by IDT and cloned into the pHR-dSV40 vector backbone in frame with mEGFP using Gibson Assembly. Biochemical analysis of the recombinantly produced IRAK4 kinase domain with these mutations confirmed the restoration of heterodimerization with the IRAK1 kinase domain, even in the absence of IRAK4 kinase activity (see below).

### Lentiviral production and generation of stable expressing EL4 cell lines

Lentivirus particles were produced in HEK-293T cells by co-transfection of the pHR transfer plasmids with second-generation packaging plasmids pMD2.G and psPAX2 (a gift from Didier Trono, Addgene plasmid # 12259 and # 12260). Virus particles were harvested from the supernatant after 48–72 h, filtered, and applied to EL4 cells. After three days, the transduced EL4 cells were resuspended in fresh RPMI medium. After lentiviral transduction, we select positive EL4 cells expressing fluorescent protein fusion of IRAK4/1 using a BD FACS Aria II at the Deutsches Rheuma-Forschungszentrum Berlin Flow Cytometry Core Facility. Specific details of the EL4 cell line generation given below.

To engineer EL4 cells expressing IRAK4 alleles, we transduced EL4-MyD88-GFP/IRAK4 KO cells with lentiviruses encoding WT or mutant IRAK4. 1-2 weeks after lentivirus transduction, we used FACS to select cell populations with homogenous expression levels. EL4 WT cells were used as a negative control, and the EL4-MyD88-GFP/IRAK4-mScarlet knock-in cell line was used to create a sorting gate that selected transduced cells with expression levels that were equivalent to endogenously expressed IRAK4.

To engineer EL4 cells expressing IRAK2 or IRAK3 tagged with mScarlet, we transduced EL4-MyD88-GFP/IRAK1 KO cells with lentiviruses encoding WT IRAK2 or IRAK3 fused to mScarlet-i. 1-2 weeks after lentivirus transduction, we used FACS to select cell populations with homogenous expression levels. EL4 WT cells were used as a negative control, and the EL4-MyD88-GFP/IRAK1-mScarlet knock-in cell line was used to create a sorting gate that selected transduced cells with expression levels that were equivalent to endogenously expressed IRAK1.

To engineer the EL4-3xKOs expressing MyD88, IRAK4-GFP and IRAK1-mScarlet, we transduced EL4-3xKOs with lentivirus encoding MyD88-FLAG-IRES-Puromycin. After 1 week of selection with media containing 1 µg/ml of puromycin for 1 week, single cells were sorted in 96 well plates. Single cell clones that grew were assayed by western blot for MyD88 expression level that matched the MyD88-GFP/IRAK4-mScarlet EL4 cell line. Based on this analysis, we selected a single clone and transduced it with lentiviruses encoding IRAK1/2/3/4-WT/mutants tagged with mEGFP/mScarlet-i. Cells were then FACS sorted to select GFP/mScarlet positive cells after 1-2 weeks post-lentiviral transduction.

To engineer further EL4-3xKOs expressing MyD88, IRAK4-GFP and IRAK1-mScarlet, we transduced EL4-3xKOs with lentivirus encoding MyD88-FLAG-IRES-TagBFP, FACS sorted for TagBFP, transduced the BFP-positive cell population with lentiviral constructs and FACS sorted to select GFP/mScarlet positive cells after 1-2 weeks post-lentiviral transduction

### Baculovirus expression of the kinase domain of human IRAK4, and IRAK1

The kinase domains of human IRAK1 (187–527; D358A) and human IRAK4 (160–460), and all corresponding mutants, were expressed in insect cells. Bacmid DNA was generated by transformation of pFastBac vectors into DH10EMBacY E. coli cells (Geneva Biotech) and was subsequently used for baculovirus generation and amplification in Sf21 (Spodoptera frugiperda) insect cells, as described elsewhere^19,37^. Sf21 insect cells (Invitrogen) were cultured at 27 °C in Sf-900 II serum-free medium (Thermo Fisher), either as adherent cultures for bacmid transfection and initial virus amplification, or as suspension cultures for larger-scale amplification and protein expression.

Expression cultures of baculovirus-infected Sf21 cells (1-2 litres) were grown at 27 °C until the percentage of YFP-expressing cells reached > 70%. Cells were harvested by centrifugation at 2500 × *g* for 30 min at 4 °C. The resulting cell pellets were resuspended in ice-cold buffer (50 mM HEPES pH 7.5, 500 mM NaCl, 20% glycerol, 1 mM AEBSF, 1 mM benzamidine, 20 ppm Pierce Universal Nuclease (Thermo Fisher), 5 mM β mercaptoethanol) and lysed by 30 passes in a Dounce homogenizer on ice. Lysates were clarified by centrifugation at 40,000 × *g* and 4 °C for 40 min. Protein kinase domains were purified using a tandem-affinity approach in which they were first purified by an initial pass over Ni-NTA agarose resin, followed by a second purification step on GSH-4B sepharose, as described previously^38^. Protein was eluted from GSH-resin by cleavage of the 6His-GST-tag with PreScission Protease at 4 °C overnight and concentrated using 10 kDa MWCO centrifugal filter units.

### Native PAGE co-migration assay

Co-migration of IRAK1 and IRAK4 on Tris-Glycine gels under native conditions can be used to visualize their interaction^19,27^. 3 μg of IRAK1 (187–527; D358A) and 3 μg of IRAK4 (160–460) were incubated together at 30 °C for 1 h with gentle shaking in 10 μl of 35 mM HEPES pH 7.5, 5 mM MgCl2, 2.5 mM DTT. The samples were cooled to room temperature and added to 10 μl Novex (2 ×) Tris-Glycine Native Sample Buffer (Thermo Fisher). After mixing well, samples were pre-incubated on ice for 1 h prior to loading on Novex 10% Tris-Glycine gels (Thermo Fisher). Samples were separated by electrophoresis with Novex Tris-Glycine Native Running Buffer (Thermo Fisher) at a constant voltage of 150 V for 1 h 45 min at 4 °C. Gels were stained with InstantBlue Protein Stain (Abcam) and images captured using the ChemiDoc MP Imaging System (Bio-Rad).

### Assay of IL-2 release in WT and gene-edited EL4 cells

To measure IL-2 release, we used the Mouse IL-2 DuoSet ELISA kit (R&D Systems, DY402-05) using the manufacturer’s protocol. The day before IL-1 stimulation, EL4 cells were resuspended into fresh media. In experiments that measured the effect of the IRAK4 kinase inhibitor, this resuspension media contained the inhibitor (or equivalent 0.2% v/v of DMSO-only in untreated samples) at the reported concentration (Supplementary Fig. 4g). The next day, the cells were counted and resuspended in fresh media. We then seeded cells in 48-well plates with 1 × 10^6^ cells in 150 μl of media per well. Cells were allowed to settle for 30 min, before being stimulated with recombinant mouse IL-1β (PeproTech) in 50 μl medium per well at a final concentration of 5 ng/ mL. For IL-2 ELISA measurements performed with IRAK4 kinase inhibitor treated cells, the concentration of the inhibitor in the media was kept constant throughout the entire duration of the assay. For the unstimulated controls, 50 μl medium was added with/ without the inhibitor. After incubating the cells for 24 h with IL-1, the plates were centrifuged (800 x *g* for 5 min), and supernatants were transferred into a new plate. Supernatants were stored at − 80 °C until IL-2-ELISA analysis. Absorbance readings were acquired on a VersaMax Microplate Reader (Molecular Devices) at 450 nm. IL-2 release was assayed on three independent days in triplicate. The obtained results were expressed as the fold change in IL-2 release between the stimulated and unstimulated samples (Supplementary Figs. 1b, 4g).

### Imaging chambers and supported lipid bilayers

SLBs were prepared using a previously published method^39^. Phospholipid mixtures consisting of 97.5% mol 1-palmitoyl-2-oleoyl-sn-glycero-3-phosphocholine (POPC), 2% mol 1,2-dioleoyl-sn-glycero-3-[(N-(5-amino-1-carboxypentyl)iminodiacetic acid)succinyl] (ammonium salt) (DGS-NTA) and 0.5% mol 1,2-dioleoyl-sn-glycero-3- phosphoethanolamine-N-[methoxy(polyethylene glycol)−5000] (PE-PEG5000) were mixed in glass round-bottom flasks and dried down with a rotary evaporator. All lipids used were purchased from Avanti Polar Lipids. Dried lipids were placed under vacuum for 2 h to remove trace chloroform and resuspended in PBS. Small unilamellar vesicles were produced by several freeze-thaw cycles in conjunction with sonication. Once the suspension had cleared, the lipids were spun in a benchtop ultracentrifuge at 35,000 x *g* for 45 min and kept at 4 °C for up to five days.

Supported lipid bilayers (SLB) were formed in 96-well glass-bottom plates (SWISSCI). 96-well plates were cleaned for 30 min with a 5% Hellmanex solution containing 10% isopropanol heated to 50 °C, then incubated with 5% Hellmanex solution for 1 h at 50 °C, followed by extensive washing with pure water. 96-well plates were dried with nitrogen gas and sealed using clear Polyolefin sealing tape (Thermo Scientific) until needed. To prepare SLB on 96-well plates, individual wells were cut out and base etched for 15 min with 5 M KOH and then washed with water and finally PBS.

To form SLBs, SUVs suspension was deposited in each well and allowed to form for 1 hr at 45 °C. After 1 hr, wells were washed extensively with PBS. SLBs were incubated for 20 min with HEPES- buffered saline (HBS: 20 mM HEPES, 135 mM NaCl, 4 mM KCl, 10 mM glucose, 1 mM CaCl2, 0.5 mM MgCl2) containing with 5 mM NiCl2 to charge the DGS-NTA lipid with nickel. The SLBs were then washed in HBS containing 0.1% BSA to block the surface and minimize non-specific protein adsorption and incubated at room temperature for 30 min. For SLBs set up on 96-well plates, the total well volume was 690 μl. Each well was completely filled with HBS containing 0.1% BSA, and 570 μl was removed, leaving 120 μl in each well. After blocking, the SLBs were functionalized by incubation for 1 hr with 120 µl His-tagged proteins to get a final concentration of 5 nM. The labeling solution was then washed out with HBS.

### Protein expression, purification and Labeling for SLB experiments

We functionalized supported membranes with an engineered variant of mouse IL1β (in text referred to as His10-Halo-JF646-IL-1β). This IL-1 variant had been engineered to have a 10x His tag, and could therefore label membranes that contained DGS-NTA lipids. Full details on the design, expression and purification of this construct have been previously reported^11,40^. For microscopy calibration of mScarlet single molecule intensity we used His10-mScarlet-IL-1β. For mEGFP single molecule intensity calibration, mEGFP was expressed and purified as previously described (see references^11^ and^40^).

### TIRF-Microscopy data acquisition

Dual color imaging of EL4 cells was performed on an inverted microscope (Nikon TiE, Tokyo, Japan) equipped with a Nikonfiber launch TIRF illuminator. Illumination was controlled with a laser combiner using the 488, 561 and 640 nm laser lines. Fluorescence emission was collected through filters for GFP (525 ± 25 nm), RFP (595 ± 25 nm) and JF646 (700 ± 75 nm). All images were collected using a Nikon Plan Apo 100×1.4 NA oil-immersion objective that projected onto a Photometrics 95B Prime sCMOS camera with 2 × 2 binning and a 1.5 x magnifying lens (calculated pixel size of 0.147 µm). Image acquisition was performed using NIS-elements software. All experiments were performed at 37 °C. The microscope stage temperature was maintained using an OKO Labs heated microscope enclosure. Imaging frames of live cells was acquired at an interval of 3 s using exposure times of 200–400 ms.

### Imaging EL4 cells expressing tagged Myddosome components on IL-1β functionalized SLBs with TIRF-Microscopy

His10-Halo-JF646-IL1β functionalized SLBs were set up as described above and as previously described^11,40^. To quantify the IL-1β density on the SLB before each experiment, wells were prepared that were functionalized with identical labeling protein concentration and time, but with different molar ratios of labeled to unlabeled His10-Halo-IL1β. Before application of cells, SLBs were analyzed by TIRF microscopy to check formation, mobility and uniformity. Short time series were collected at wells containing a ratio of labeled to unlabeled His10-Halo-IL1β (e.g., < 1 His10-Halo-JF646-IL1β molecule per µm2) to calculate ligand densities on the SLB based upon direct single molecule counting. All experiments were performed at IL-1 SLB densities of 50–80 molecules per µm2.

Before each imaging experiment, we acquired calibration images using recombinant mEGFP and mScarlet-i. To image single GFP/mScarlet-i fluorophores, recombinant purified mEGFP and mScarlet-i^40^ was diluted in HBS and adsorbed to KOH-cleaned glass. Single molecules of GFP or mScarlet-i were imaged using identical microscope acquisition settings to those used for cellular imaging. To image live cells, EL4 cells were pipetted onto supported lipids bilayers functionalized with His10-Halo-JF646-IL1β. EL4 cells expressing complementary GFP- and mScarlet-tagged Myddosome components (e.g., MyD88–GFP together with wild-type or mutant IRAK4–mScarlet or WT or mutant IRAK4-GFP with IRAK1/2/3–mScarlet) were sequentially illuminated with 488 nm (70–100 ms) and 561 nm (100 ms) lasers at 3 s frame intervals.

For pharmacological inhibition experiments, EL4 cells were treated with increasing concentrations of the IRAK4 kinase inhibitor PF06650833 (Sigma) for at least 3 h prior to imaging (Fig. 3j, k, and l). All treatment conditions contained 0.1% DMSO (v/v). Before imaging, cells were pelleted and resuspended in HBS containing the corresponding inhibitor concentration. The resuspended cells were then added to imaging wells containing HBS supplemented with the same inhibitor concentration, thereby maintaining the dose response conditions throughout microscopy acquisition.

### Live cell single molecule imaging of IRAK1, IRAK2 and IRAK3 kinase domain recruitment

His10-Halo-JF646–IL1β–functionalized SLBs were prepared on 25 mm round nano-printed coverslips with a chromium grid, as previously described^40^. Gridded coverslips were used to limit the lateral movement of MyD88-GFP puncta. This stabilization allowed the position of partially assembled Myddosomes to be identified and then interrogated using high-frame-rate imaging of the mScarlet channel to detect transient recruitment of the IRAK1/2/3 kinase domain. EL4-MyD88-GFP/IRAK1-KO cells reconstituted with IRAK1/2/3-ΔDD-mScarlet were allowed to settle on the coverslips for 5–10 min. Fields of view containing cells with MyD88-GFP puncta (i.e., partially assembled Myddosomes) were selected.

A reference image was acquired in the GFP channel and widefield (for cell boundaries), followed by continuous imaging in the mScarlet channel. For IRAK1/2/3-ΔDD-mScarlet, an exposure time of 200 ms was used. In this modality, 1200 frames were acquired per field. Data were collected from cells treated with the IRAK4 kinase inhibitor (as described above) or with an equivalent concentration of DMSO.

To analyze IRAK1/2/3 kinase domain recruitment, the MyD88-GFP reference images were used to manually segment cells in the mScarlet channel. The segmented image stacks corresponding to the cell footprints on the bilayer were processed using TrackMate in FIJI to detect and track IRAK1/2/3 kinase domain recruitment events. From the resulting data files, the mean number of recruitment events per cell (Fig. 4d, g, j) and the lifetime of recruitment events (Fig. 4e, h, k) were calculated.

### Immunofluorescence staining and quantification of phospho-IRAK4

To analyze the colocalization of phospho-IRAK4 with MyD88-GFP and IRAK4-mScarlet or IRAK1-mScarlet (Fig. 4a, b), EL4 cells were stimulated with IL-1β-functionalized SLBs for 30 mins, and then fixed with 4% (wt/vol) PFA containing 0.5% (wt/vol) Triton X-100 for 20 min at room temperature. Staining was then performed with a traditional two-step staining method. After fixation, cells were washed with PBS, then blocked in PBS containing 10% (wt/vol) BSA and 0.1% Triton X-100 at 4 °C overnight. The next day, the cells are stained using a 1 step labeling procedure with antibody solution containing anti-pIRAK4 (1:400, Vikram Rao, Pfizer), Nano-Secondary 647 (1:1000, ChromoTek alpaca anti-human IgG/anti-rabbit IgG, recombinant VHH, Alexa Fluor 647 CTK0101, CTK0102 #srbAF647-1) and FluoTag-X4 anti-GFP conjugated to Atto488 (1:500, Nano Tag Biotechnology, #N0304-At488-L) and FluoTag-X4 anti-RFP conjugated to Atto568 (1:500, Nano Tag Biotechnology, #N0404-AF568-L) in PBS containing 10% (wt/vol) BSA and 0.1% Triton X-100 for 1 hr at room temperature. Subsequently, cells were washed with 2 ml 10% (wt/vol) BSA and 0.1% Triton X-100 and finally with 4 ml PBS before imaging with TIRF microscopy.

To score MyD88 puncta negative or positive for phospho-IRAK4 and IRAK4-mScarlet or IRAK1-mScarlet, TIRF microscopy images were manually segmented into individual cells. The phosphoIRAK4 and IRAK4-mScarlet or IRAK1-mScarlet channels were blinded, and then MyD88 puncta manually identified and scored negative or positive for colocalization with puncta in the blinded phospho-IRAK4 channel and IRAK4-mScarlet or IRAK1-mScarlet channel. Manual identification and scoring was performed in FIJI with the multi-point annotation tool. We blinded the IRAK4/1-mScarlet and pIRAK4 staining channels before manual scoring of MyD88-GFP puncta. We performed this manual analysis for over 400 MyD88-GFP puncta identified in both EL4-MyD88-GFP/IRAK4-mScarlet and EL4-MyD88-GFP/IRAK1-mScarlet cells. Unblinded manual scoring of MyD88-GFP puncta was visualized as a bar graph (Fig. 4a, b).

### Quantification and statistical analysis

All data are expressed as the mean ± the standard deviation (SD) or mean ± the standard error of the mean (SEM), as stated in the figure legends and results. The exact value of n and what n represents (such as the number of experimental replicates, cells or MyD88-puncta) is stated in figure legends and results. Means were compared using Wilcoxon Rank-Sum test, due to the small n size of replicate making it difficult to determine whether the data is normally distributed. Data analysis scripts used in this study available at GitHub.

### Quantification and analysis of Myddosome intensity, lifetime and dynamics

To quantify the dynamics of Myddosome assemblies labeled with fluorescently tagged MyD88 or IRAKs, we created an image analysis pipeline that runs in FIJI, Python and R. Image analysis scripts were parallelized so that they may run on cluster computers at the Max Planck Computing and Data Facility (Garching, Bavaria, Germany). The aim of this image analysis pipeline was to first process the images to remove intensity count that derived from camera noise or background fluorescence (non-specific cytosolic fluorescent signal), identify and segment single cells, and then identify and track the individual fluorescently labeled Myddosome complexes within the live cell data. To process the image stack, we first subtracted a dark frame image (i.e., an image only containing intensity values from current and noise generated by the camera electronics) from each image file. The dark frame image was created by calculating the mean intensity from 5000 images acquired without light exposure to the camera, and with identical acquisition settings to those used for live cell imaging.

From this point, dark frame-subtracted images were processed to create two sets of image stacks: one set was used for intensity measurements to quantify the dynamics of Myddosome assembly (referred to as the intensity reference image). The second set was processed using different filters and parameters best suited to detect punctate structures, and this was used to optimally identify and track the Myddosomes (referred to as the tracking images). To generate an image stack for tracking Myddosome puncta, the GFP/mScarlet images were processed with a median filter with a 11-pixel diameter. The resulting median filtered image is subtracted from the original to remove background intensity. We next applied two methods to address stochastic fluctuation associated with the fluorophore blinking or camera read noise, both which can contribute to errors in particle detection and linking detected particles between frames. First, we applied a second median blur of 5-pixel diameter to even out the field. Because median filters are edge-preserving (keeps silhouette), a 5-pixel diameter median blur preserved punctate structures while further removing background noise. We then applied a moving average of 3 frames to further suppress intensity fluctuations. Because we were interested in detecting Myddosomes (i.e., puncta that possibly contain both MyD88 and IRAK4 or IRAK4 and IRAK1/2/3), we reasoned that combining the GFP and mScarlet channels into a single image would further increase the signal to noise ratio, facilitating the detection and tracking of Myddosome fluorescent puncta. Therefore, all GFP and mScarlet image frames were summed. The result was a single time series with high signal to noise and optimized for particle detection and tracking.

The combination of two median blur filtering steps and a running average used to create the tracking reference image skewed the intensity values. Therefore, to be able to accurately quantify Myddosome intensity and assembly kinetics, we generated an intensity reference image that preserved intensity values. To accomplish this, we estimate cytosolic background using a median blur with a 25-pixel diameter (3.67 µm), which is roughly half the diameter of the cell: SLB interfaces observed in the TIRF field. This median blurred image is an estimate of background intensity from the cytoplasmic fluorescence (that is GFP or mScarlet tagged MyD88 or IRAKs not assembled into Myddosome complexes). This estimated background fluorescent intensity is subtracted from the dark frame removed images, and the resulting image is the intensity reference image from which all shown and analyzed intensity values are derived.

Next, individual cells within the time series were identified using a marker-controlled watershed segmentation (algorithm from Open Source Computer Vision, OpenCV) implemented on a maximum projection image of the GFP intensity channel. The segmented cell boundaries were used to break both the tracking image and intensity reference series into small image series of individual cells. We used the FIJI TrackMate plugin to track the MyD88-GFP/IRAK4/1-Scarlet or IRAK4-GFP/IRAK1/2/3-mScarlet puncta within the tracking image for each segmented cell. We tracked MyD88-GFP/IRAK4/1-mScarlet puncta with a maximum linkage distance of 5 pixels, a tracking threshold of 1.5 and a tolerance of 2 gap-closing frames between detected particles. After processing in TrackMate, the tracking coordinates generated were imported into R. To fill in missing frames (i.e., gap-closing frames with no detected particles) within tracks, we approximated the location of the missing puncta using the Euclidean distance from the coordinates of the preceding and following detected particles. We extracted the fluorescence intensity of puncta from the intensity reference frames of both the MyD88-GFP image and the IRAK4/1-mScarlet image. The intensity was measured from a 5 × 5 circular pixel region center of the puncta centroid. To achieve sub-pixel accuracy, we segmented a square matrix of pixels from images. This square was centered on the puncta centroid. To reduce computational time, this segmented area was expanded 11 fold with the original pixel intensity subdividing in the expanded area pixels. The square was then multiplied against a binary circle mask (diameter of 5 pixels that was also expanded by 11 fold). The pixel intensities of the product were summed to give a puncta intensity for both the GFP or mScarlet channel.

To estimate the number of MyD88 and IRAKs within tracked Myddosome assemblies, the fluorescent intensity was divided by the median intensity of single GFP and mScarlet fluorophores. To compute the distribution of single fluorophore intensities, images of single GFP or mScarlet fluorophores adsorbed to glass were processed and analyzed identically to MyD88-GFP, IRAK4-GFP and IRAK4/1/2/3-mScarlet images. We restricted the analysis to MyD88-GFP and IRAK4/1-mScarlet puncta tracked for three or more frames, to focus our analysis to bona fide Myddosome assemblies nucleating at the plasma membrane. Puncta were scored positive for IRAK4/1/2/3 recruitment when the normalized fluorescent intensity of at least 1.5x IRAK4/1 s for 30 seconds or more (Figs. 1d, g, j, 2e, h, k, n, 3c, f, i, j through m, Fig. 5e, Supplementary Fig. 3C, E, Supplementary Fig. 4H, and Supplementary Fig. 5e). IRAK4/1/2/3 dwell time distribution was calculated by summing consecutive frames where the IRAK4/1/2/3 signal intensity was above this fluorescent intensity threshold (Figs. 1c, f, i, 2d, g, j, m, 3b, e, h, 5d, Supplementary Fig. 3b and Supplementary Fig. 5d). To calculate the size distribution of IRAK1/2/3 assemblies we plotted the (Supplementary Fig. 2d through g and Supplementary Fig. 5f). We restricted this analysis to IRAK4 puncta which recruit IRAK1/2/3 with a size of 0.75 x or above. We performed data visualization using ggplot2, a data visualization package for R.

### Reporting summary

Further information on research design is available in the Nature Portfolio Reporting Summary linked to this article.

## Supplementary information

Supplementary Information

Transparent Peer Review file

Reporting Summary

Description of Additional Supplementary Files

Supplementary Movie 1

Supplementary Movie 2

Supplementary Movie 3

Supplementary Movie 4

Supplementary Movie 5

Supplementary Movie 6

Supplementary Movie 7

## Source data

Source Data

## Data Availability

Uncropped blots showing the regions of interest used to make the figures are in the Source data file. Raw microscopy imaging data, analyzed imaging files and original full length Western blots images used in this study were deposited to Zenodo: Fig. 1 and Fig. 4: https://doi.org/10.5281/zenodo.21475178; Fig. 2https://doi.org/10.5281/zenodo.21531017 (part 1) and https://doi.org/10.5281/zenodo.21532193 (part 2); Fig. 3https://doi.org/10.5281/zenodo.21621840 (part 1), https://doi.org/10.5281/zenodo.21623511 (part 2), https://doi.org/10.5281/zenodo.21626950 (part 3), https://doi.org/10.5281/zenodo.21639682 (part 4), https://doi.org/10.5281/zenodo.21643038 (part 5) and https://doi.org/10.5281/zenodo.21644619 (part 6); Fig. 5https://doi.org/10.5281/zenodo.21620828; Supplementary Figs. 1, 2, 5, 6 (part 1) https://doi.org/10.5281/zenodo.21470271; Supplementary Fig. 4https://doi.org/10.5281/zenodo.21510430. The exception is primary single-molecule microscopy data for Fig. 4a through k, which, due to the large file sizes, was not uploaded to an online repository and is available upon request. The source data for making the figures is freely available as a Source Data file. Source data are provided in this paper.

## Code availability

Computer code is available from GitHub [https://github.com/MJ-Taylor-Lab/IRAK4_Autophosphorylaytion_Paper].
