# Supplementary material for: IRAK4 autophosphorylation functions as a molecular switch to regulate death domain assembly and Myddosome maturation
Source: Nat Commun. 2026 Oct 21;17:11001. doi: 10.1038/s41467-026-78773-6 (PMC13687090; doi:10.1038/s41467-026-78773-6)
Supplement: Supplementary file 1 [file 41467_2026_78773_MOESM1_ESM.pdf]

Supplementary Information for

IRAK4 autophosphorylation functions as a molecular switch to regulate death domain assembly and Myddosome maturation

Niranjan Srikanth[1,2], Rafael Deliz-Aguirre[1,3], Deepika Kumari Gola[1], Margaux Bilay[1], Elke Ziska[1], Ian Kelsall[4], Yogesh Kulathu[4], and Marcus J. Taylor[1]*

[1]Max Planck Institute for Infection Biology, Chariteplatz 1, Berlin D-10117, Germany
[2]Department of Biology, Chemistry and Pharmacy, Freie Universität Berlin, Berlin 14195, Germany
[3]Humboldt University of Berlin, Unter den Linden 6, Berlin 10117, Germany
[4]MRC Protein Phosphorylation and Ubiquitylation Unit, School of Life Sciences, University of Dundee, Dundee DD1 5EH, United Kingdom

*e-mail: taylor@mpiib-berlin.mpg.de

The PDF File includes:

Supplementary Fig. 1 to 6
Supplementary Table 1 to 2

Supplementary Figure 1

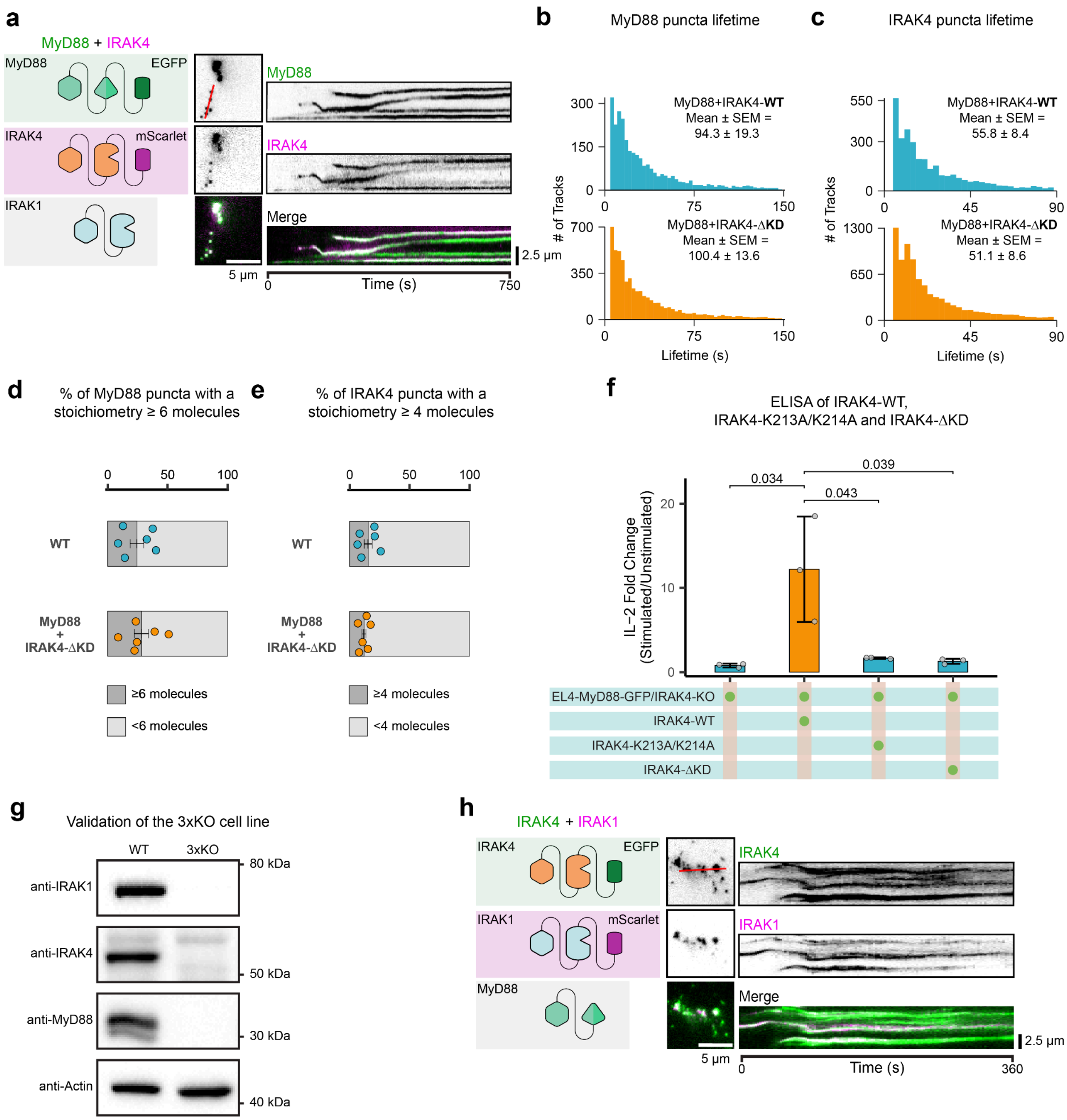

Supplementary Fig. 1. Analysis of IRAK4 mutants and validation of 3xKO cell line.

a) TIRF images of EL4-MyD88-GFP/IRAK4-mScarlet stimulated on IL-1 functionalized SLBs. . Kymographs derived from red line overlaid TIRF images (left panel). Scale bars 5 µm (left panel) and 2.5 µm (right panel).

b-c) Histograms showing the lifetime distribution of MyD88 (e) and IRAK4 (f) puncta in co-assemblies.

d-e) Percentage of MyD88 (g) and IRAK4 (h) co-assemblies with a stoichiometry greater than or equal to 6 and 4 molecules, respectively, for MyD88 and IRAK4 — a stoichiometry consistent

with fully assembled Myddosomes. Data represent n = 6 independent replicates for each allelic pairing shown in panel (D) and (E).

f) IL-2 release in EL4-MyD88-GFP/IRAK4-KO cells reconstituted with IRAK4-WT-mScarlet, IRAK4-K213/14A-mScarlet and IRAK4-ΔKD-mScarlet. Values shown are the fold change in IL-2 release (n = 3 independent replicates measured per cell line). The bars represent Mean Fold change ± SD are 0.78 ± 0.22, 12.21 ± 6.25, 1.66 ± 0.08 and 1.28 ± 0.29 for IRAK4-KO, IRAK4-WT-mScarlet, IRAK4-K213A/K214A-mScarlet and IRAK4-ΔKD-mScarlet respectively. One-way Anova was used to compare the replicate means.

g) Western blot validation of a EL4 cell clone that is triple KO for MyD88, IRAK4 and IRAK1.

h) TIRF images of EL4-3xKO cells reconstituted with wild type MyD88, IRAK4-GFP and IRAK1-mScarlet stimulated on IL-1 functionalized SLBs. Kymographs derived from red line overlaid TIRF images (left panel). Scale bars 5 μm (left panel) and 2.5 μm (right panel).

Supplementary Figure 2

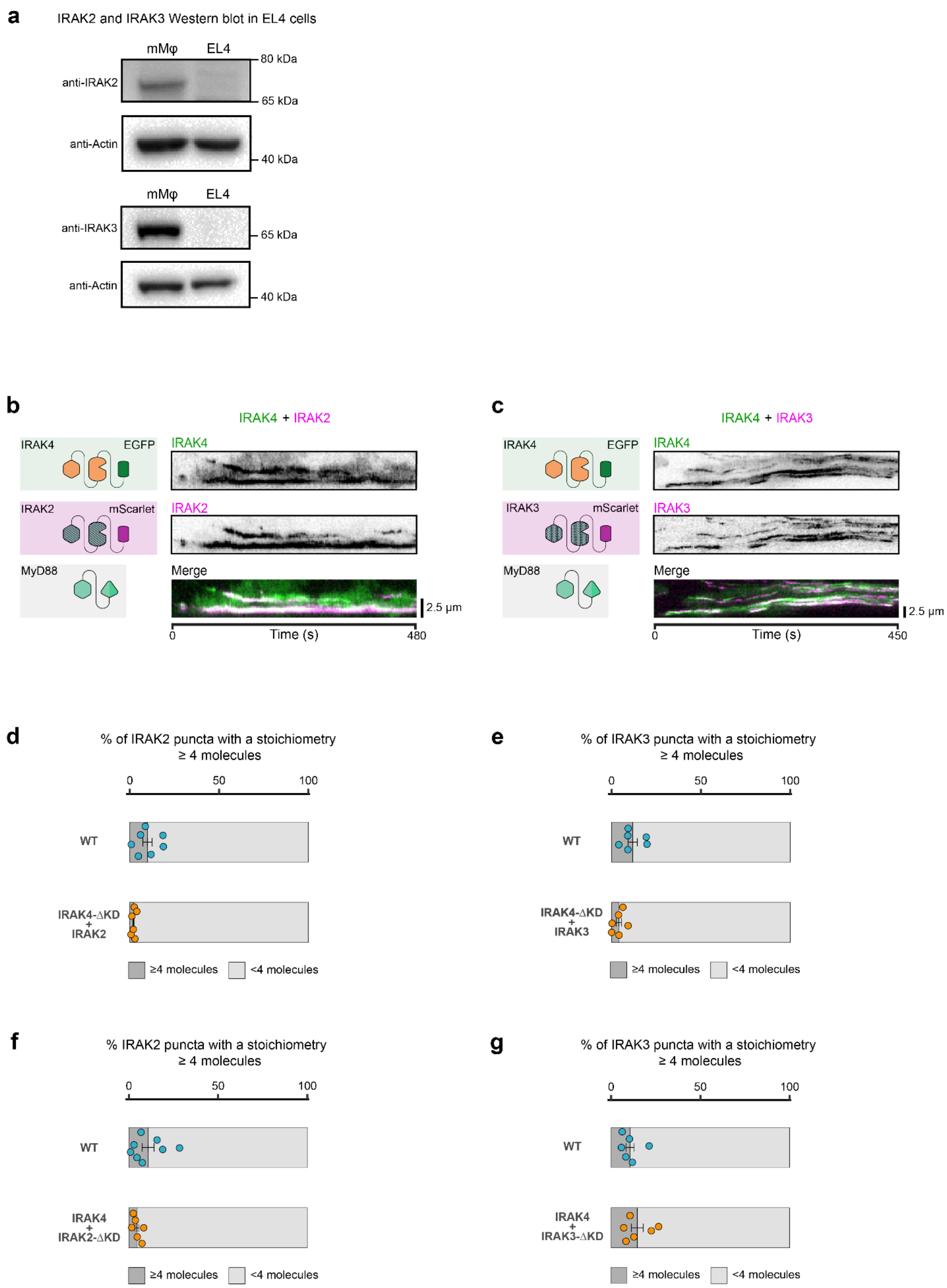

Supplementary Fig. 2: The kinase domain of IRAK4 regulates the recruitment and oligomerization of IRAK2 and IRAK3 at assembling Myddosomes.

A) Western blot analysis of IRAK2 and IRAK3 expression in EL4 cells. Lysates from bone derived mouse macrophages included as a positive control.

B-C) TIRF images and kymograph analysis of EL4-3xKO cells reconstituted with wild type MyD88 IRAK4-GFP and IRAK2-mScarlet (B) or IRAK3-mScarlet (C) stimulated on IL-1

functionalized SLBs. Kymographs derived from red line overlaid TIRF images (left panel). Scale bar, 5 μm (left panels) and 2.5 μm (right panels).

D-E) Percentage of IRAK2 (D) and IRAK3 (E) assemblies recruited to IRAK4 with a stoichiometry greater than or equal to 4 molecules, a stoichiometry consistent with fully assembled myddosomes. Data acquired from EL4-3xKO reconstituted with MyD88, IRAK4-GFP or IRAK4-ΔKD-GFP and IRAK2-mScarlet (D) or IRAK3-mScarlet (E). Mean percentages of stable IRAK2/ IRAK3 sizes ± SEM were 9.94 ± 2.60, 2.18 ± 0.48, 11.74 ± 2.61, 3.93 ± 1.44 for IRAK4-FL + IRAK2, IRAK4-ΔKD + IRAK2, IRAK4 + IRAK3, IRAK4-ΔKD + IRAK3 respectively. Data points superimposed on the bar plots show the mean of individual experimental replicates (n = 7, 6, 6 and 6 independent experimental replicates for cells expressing IRAK4 + IRAK2, IRAK4-ΔKD + IRAK2, IRAK4 + IRAK3, IRAK4-ΔKD + IRAK3 respectively).

F-G) Percentage of IRAK2/IRAK3 assemblies with a stoichiometry greater than or equal to 4 molecules recruited to IRAK4. Data acquired from EL4-3xKO reconstituted with MyD88, IRAK4-GFP and IRAK2-mScarlet or IRAK2-ΔKD-mScarlet (F) and IRAK3-mScarlet or IRAK3-ΔKD-mScarlet (G). Mean percentages of stable IRAK2/ IRAK3 sizes ± SEM were 10.74 ± 3.35, 4.64 ± 1.08, 10.61 ± 2.34, 14.64 ± 3.28 for IRAK4 + IRAK2, IRAK4 + IRAK2-ΔKD, IRAK4 + IRAK3, IRAK4 + IRAK3-ΔKD respectively. Data points superimposed on the bar plots show the mean of individual experimental replicates (n = 8, 6, 6 and 6 independent experimental replicates for cells expressing IRAK4 + IRAK2, IRAK4 + IRAK2-ΔKD, IRAK4 + IRAK3, IRAK4 + IRAK3-ΔKD respectively).

Supplementary Figure 3

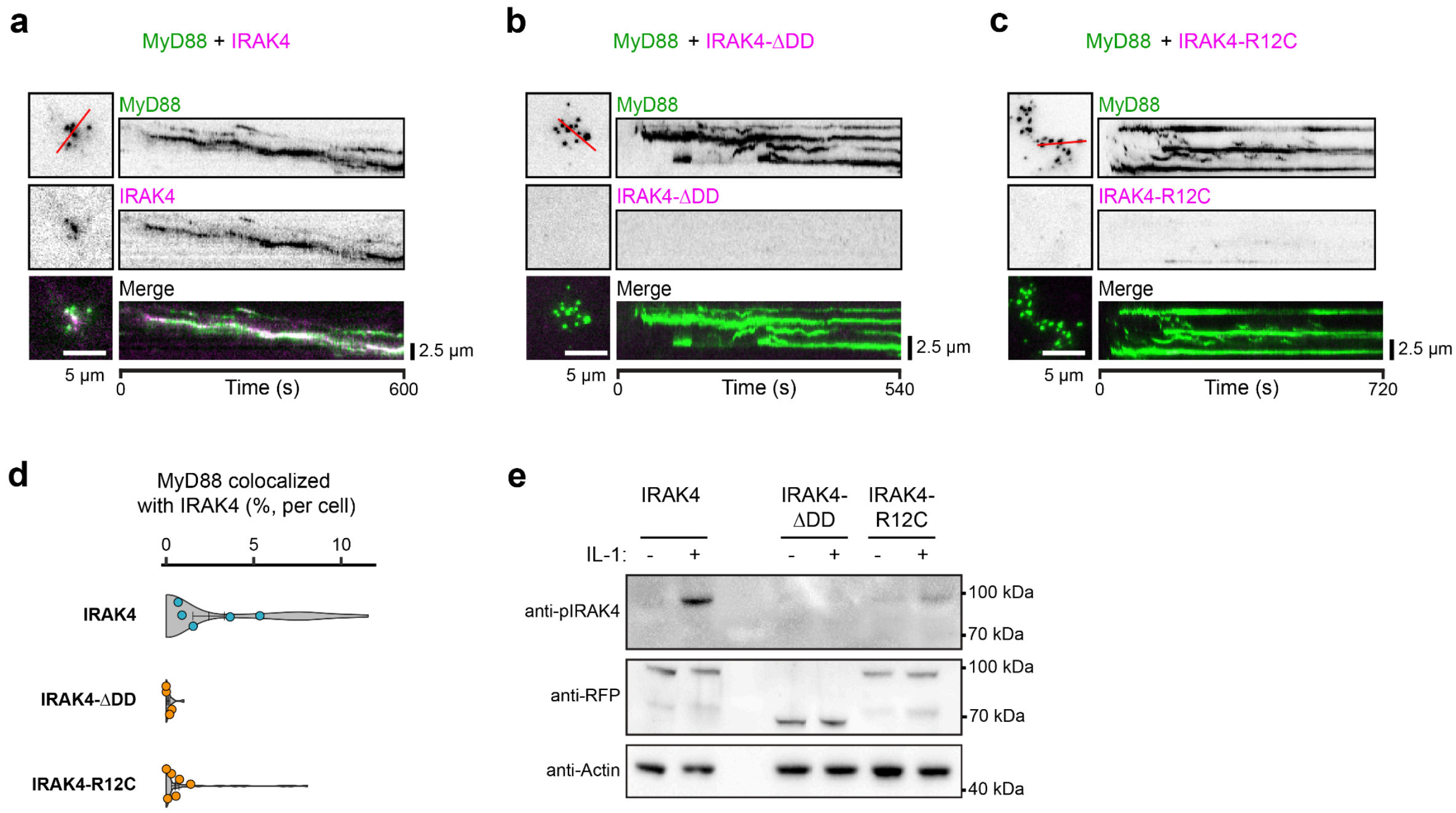

Supplementary Fig. 3. IRAK4 requires a functional DD for co-assembly with MyD88 and autophosphorylation

a-c) IRAK4 requires a functional DD to be recruited to MyD88. TIRF images and kymograph analysis (derived from red line) of EL4-MyD88-GFP/IRAK4-KO reconstituted with IRAK4-WT-mScarlet (A), IRAK4-IRAK4-ΔDD-mScarlet (B) and IRAK4-R12C-mScarlet (C). Scale bar, 5 µm (left panels). Scale bar, 2.5 µm (right panels).

d) The proportion of all MyD88 tracks that stably recruit (≥ 30) IRAK4 in EL4-MyD88-GFP/IRAK4-KO reconstituted with IRAK4-WT-mScarlet, IRAK4-ΔDD-mScarlet and IRAK4-R12C-mScarlet. Mean percentages of stable recruitment ± SEM were 2.43 ± 0.90, 0.13 ± 0.08 and 0.52 ± 0.21 for IRAK4-WT-mScarlet (n = 5 independent replicates), IRAK4-ΔDD-mScarlet. (n = 4 independent replicates) and IRAK4-R12C-mScarlet (n = 6 independent replicates) respectively.

e) IRAK4 autophosphorylation requires a functional DD that can co-assemble with MyD88. Western blot validation of pIRAK4 in EL4-MyD88-GFP/IRAK4-KO reconstituted with IRAK4-WT-mScarlet, IRAK4-ΔDD-mScarlet and IRAK4-R12C-mScarlet.

Supplementary Figure 4

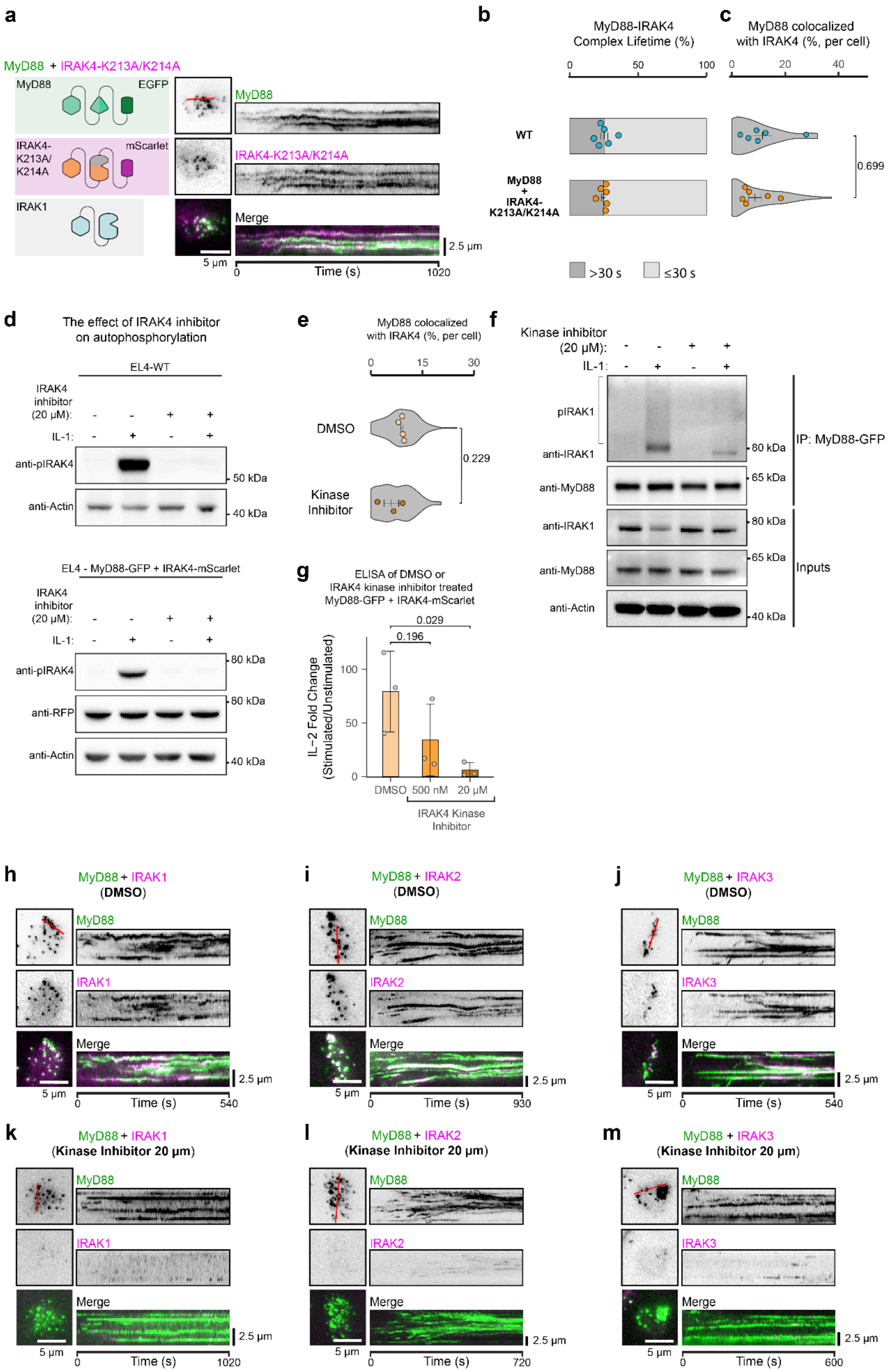

Supplementary Fig. 4: Analysis of IRAK4 kinase-inactive mutants and pharmacological inhibition

a) Time-series TIRF images and fluorescence-intensity time series showing the formation and coassembly of MyD88-GFP and IRAK4-mScarlet puncta from cells treated with DMSO (left panel) . Scale bar, 1 µm.

b) Proportion of MyD88:IRAK4-FL (top) and MyD88:IRAK4-K213A/K214A-mScarlet (bottom) colocalized puncta with lifetimes less than or greater than 30s (25.07 ± 2.56 vs. 24.68 ± 1.31 for IRAK4-FL-mScarlet (n = 6) and IRAK4-K213A/K214A-mScarlet (n = 6 independent replicates, mean ± SEM) respectively). The dots superimposed on the bar plots represent the mean proportion of each replicate.

c) The percentage of total MyD88 puncta stably colocalized with IRAK4-FL versus RAK4-K213A/K214A (11.48 ± 3.52 vs. 8.72 ± 2.37 for IRAK4-FL-mScarlet (n = 6 independent replicates) and IRAK4-K213A/K214A-mScarlet (n = 6 independent replicates, mean ± SEM) respectively). Two-tailed Wilcoxon Ranked sum test was used to compare the replicate means.

d) Western blot analysis of IRAK4 autophosphorylation (pIRAK4) production after IL-1β stimulation showing that IRAK4 kinase inhibitor blocks pIRAK4. Western blot analysis performed in EL4-WT (upper blot) and EL4-MyD88-GFP/IRAK4-FL-mScarlet (lower blot) cell lysates. Cells were treated with 20 µM of IRAK4 kinase inhibitor or DMSO, for 4 hours, before being stimulated with 1 ng/ml of IL-1β for 30 mins.

e) Proportion of MyD88:IRAK4-FL colocalized puncta with lifetimes less than or greater than 30s in cells treated with DMSO (top) or IRAK4 kinase inhibitor (bottom). The dots superimposed on the bar plots represent the mean proportion of each replicate. Mean percentages of stable recruitment ± SEM were 9.00 ± 0.36 and 5.92 ± 2.15 for DMSO (n = 4 independent replicates) and Kinase Inhibitor (n = 3 independent replicates) treated cells respectively. One-way Anova was used to compare the replicate means.

f) Myddosomes were immunoprecipitated from EL4-MyD88-GFP cell lysates treated without or with IRAK4 kinase inhibitor (20 µM) and analyzed by western blot. Treatment with the IRAK4 kinase inhibitor reduced co-assembly and the post-translational modification of IRAK1 within Myddosomes.

g) Dose dependent decrease of cytokine release in response to IRAK4 Kinase Inhibitor (500 nM and 20 µM). IL-2 release was measured by ELISA 24 h after IL-1β stimulation in EL4-MyD88-GFP/IRAK4-FL-mScarlet cells. Values shown are the fold change in IL-2 release (n = 3 independent replicates measured per cell line). Mean Fold change ± SD are 79.24 ± 37.64, 34.08 ± 33.63 and 6.07 ± 6.68 for cells treated with DMSO, and 500 nM or 20 µM of IRAK4 Kinase Inhibitor respectively.

h-m) IRAK4 Kinase Inhibition reduces recruitment of IRAK1, IRAK2 and IRAK3. TIRF images and kymograph analysis (derived from red line) of MyD88-GFP/IRAK1-FL-mScarlet (H and K), EL4-MyD88-GFP/IRAK1-KO reconstituted with IRAK2-mScarlet (I and M) or IRAK3-mScarlet (J and M). Cells are treated with DMSO (H-J) or IRAK4 Kinase Inhibitor (20 µM, K-M). Scale bar, 5 µm (left panels). Scale bar 5 µm (left panels) and 2.5 µm (right panels).

Supplementary Figure 5

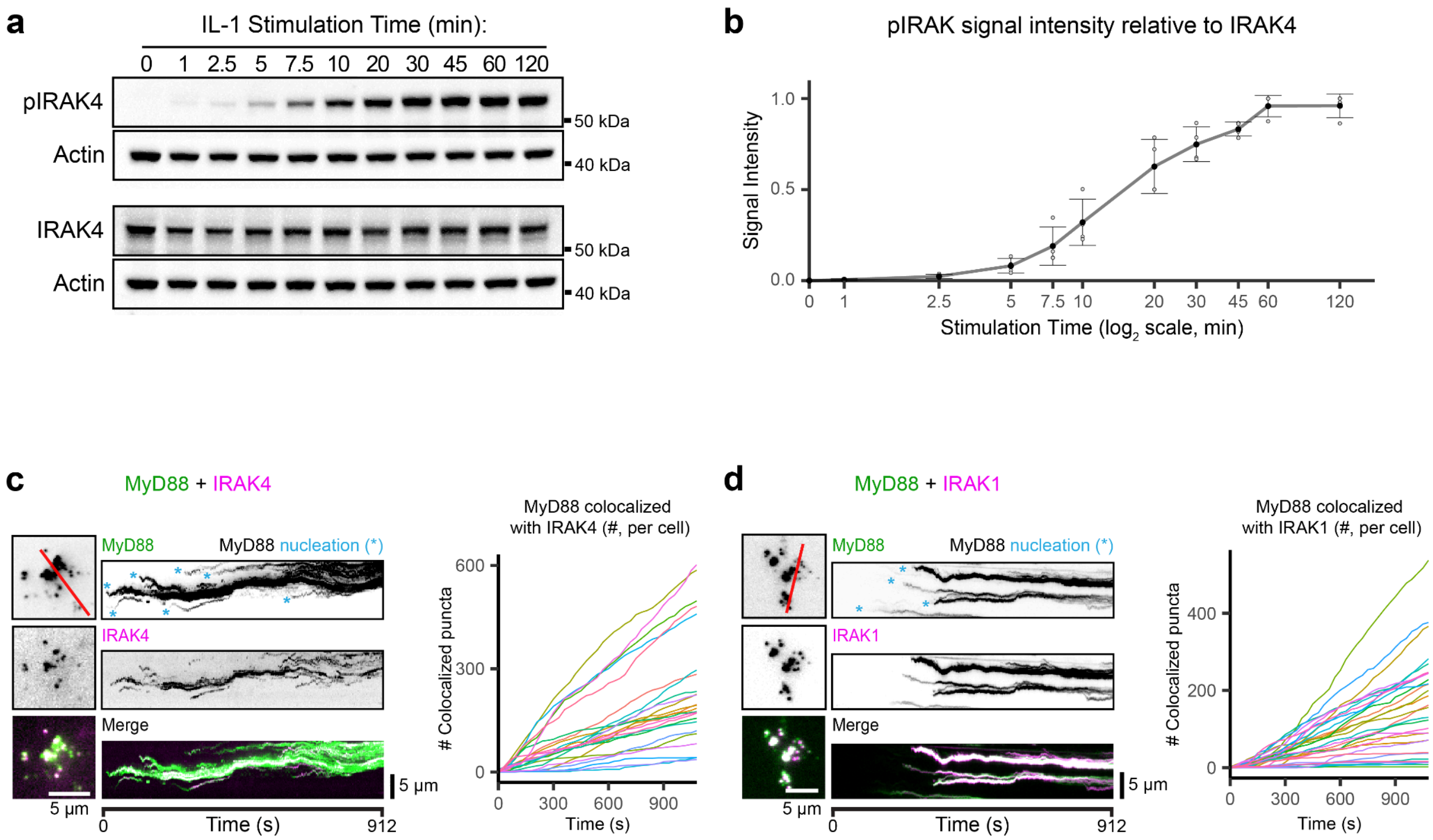

Supplementary Fig. 5. Time course analysis of IRAK4 autophosphorylation and Myddosome assembly

A) Time course Western blot analysis of pIRAK4 levels in EL4 cells after IL-1 stimulation (1 ng/ml). Lysates were separately probed for antibodies specific for pIRAK4 (Thr-345/Ser-346) and total IRAK4. Actin detection was used as a loading control and to normalize the band intensity.

B) Time course quantification of the pIRAK4 signal intensity relative to total IRAK4 signal intensity from the Western blots shown in (C). Each black colored data point is the average relative pIRAK level derived from n=4 experimental replicates (see Appendex_A). Smaller data points are the relative pIRAK signal intensity of individual replicates.

C-D) Nucleation of MyD88 and formation of myddosomes are asynchronous. (Left panels) TIRF images and kymograph analysis (derived from red line) of MyD88-GFP/IRAK4-mScarlet (A) and MyD88-GFP/IRAK1-mScarlet (B). Scale bar, 5 µm. The blue asterisks on the MyD88-GFP kymograph highlight the nucleation of Myddosome complexes. (Right panel) Formation of MyD88-GFP:IRAK4-mScarlet (A) and MyD88-GFP:IRAK1-mScarlet (B) assemblies over time for individual cells. Each color represents one of 24 (A) or 31 cells (B). t = 0 is defined as the time point when the first MyD88 puncta is observed in each cell.

Supplementary Figure 6

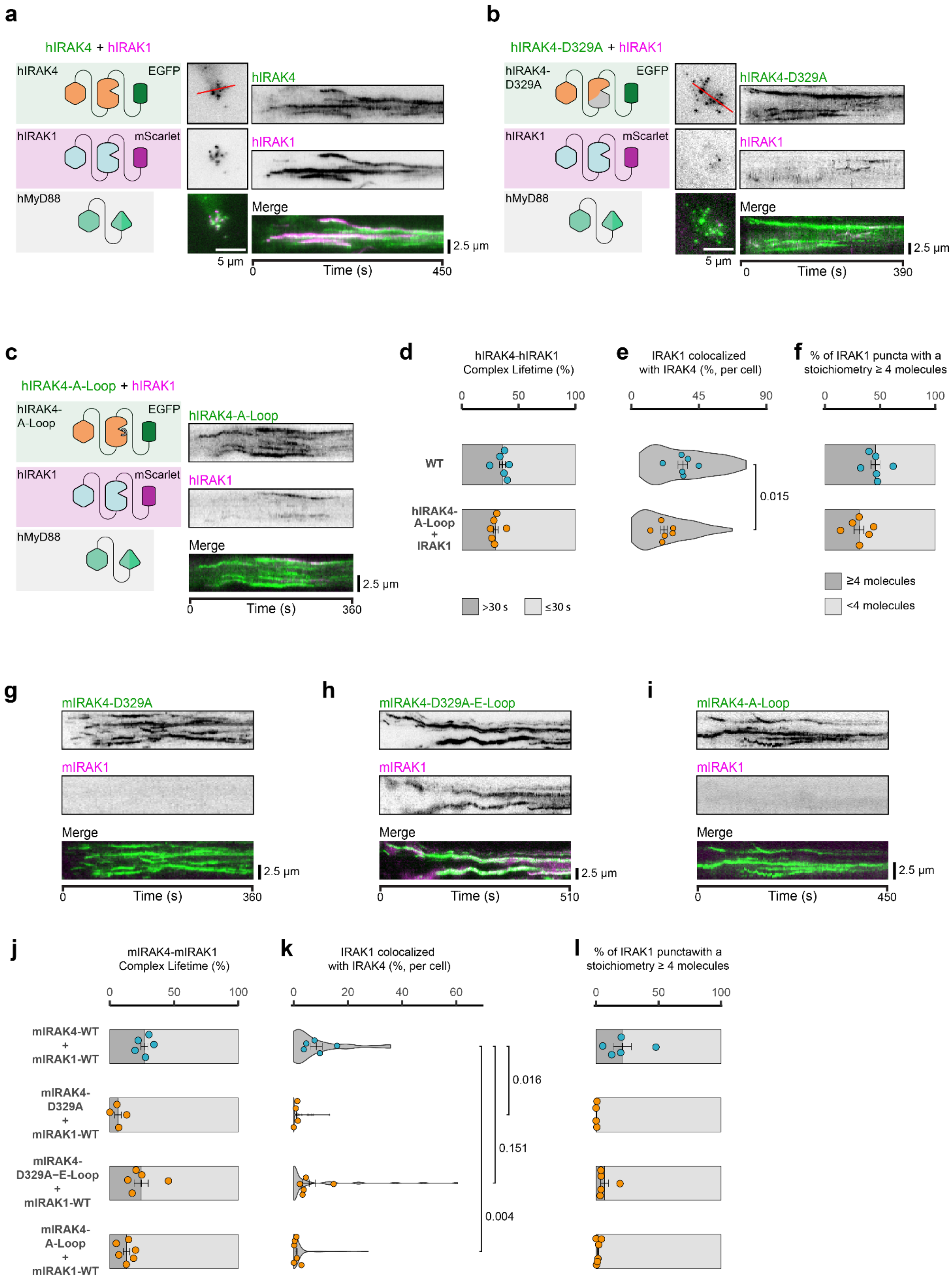

Supplementary Fig. 6: Analysis of human and mouse kinase inactive and activation loop IRAK4 mutants

A-C) TIRF images and kymograph analysis of EL4-3xKO cells reconstituted with hMyD88, hIRAK1-mScarlet, and hIRAK4-GFP variants (WT, kinase dead [D329A], and alanine activation loop mutants [T343A/T345A/S346A]) are shown. The kymograph was taken from the red line overlaying the MyD88-GFP image in each panel. Scale bar: 5 µm (left panels), 2.5 µm (right panels).

D) Proportion of IRAK4-IRAK1 colocalized puncta with lifetimes ≤30 s or >30 s for IRAK4-WT versus IRAK4-A-loop mutant paired with IRAK1-WT. Data points represent replicate means, n = 6 independent replicates for each allelic pairing shown.

E) Percentage of total IRAK4 puncta stably colocalized with IRAK1-WT for IRAK4-WT versus IRAK4-A-loop mutant. Violin plots show single-cell distributions with replicate means superimposed, n = 6 independent replicates for each allelic pairing shown.

F) Percentage of IRAK1-WT assemblies recruited to IRAK4-WT versus IRAK4-A-loop mutant with a stoichiometry greater than or equal to 4 molecules, a stoichiometry consistent with fully assembled myddosomes. n = 6 independent replicates for each allelic pairing shown.

G-L) Analysis of kinase-inactive and E/A-loop mutants of mouse IRAK4 shows that the E-loop phosphomimetic mutation can restore IRAK1 recruitment and incorporation into Myddosomes. G-H) Kymograph analysis of EL4-3xKO cells reconstituted with mMyD88, mIRAK1-mScarlet, and mIRAK4-GFP variants (WT, kinase-dead [D329A], and alanine activation loop mutants [T343A/T345A/S346A]). Scale bar: 2.5 µm (right panels). J) Proportion of mouse IRAK4-IRAK1 colocalized puncta with lifetimes ≤30 s or >30 s for IRAK4-WT versus IRAK4-A-loop mutant paired with IRAK1-WT. Data points represent replicate means. K) Percentage of total mouse IRAK4 puncta stably colocalized with IRAK1-WT for IRAK4-WT versus IRAK4-A-loop mutant. Violin plots show single-cell distributions with replicate means superimposed, L) Percentage of mouse IRAK1-WT assemblies recruited to IRAK4-WT versus IRAK4-A-loop mutant with a stoichiometry greater than or equal to 4 molecules, a stoichiometry consistent with fully assembled Myddosomes. n = 5, 4, 5, and 6 independent replicates for mouse WT, kinase-dead [D329A], E-loop mutant and alanine activation loop mutants [T343A/T345A/S346A] respectively in panel (J), (K) and (L).

Each independent replicate for each allelic pairing consists of 9-28 cells per replicate. Statistical significance was assessed using a two-tailed Wilcoxon rank-sum test in panel E and K.

Supplementary Table 1. Key resources and materials.

REAGENT or RESOURCE	SOURCE	IDENTIFIER
**Antibodies**		
Rabbit polyclonal anti-IRAK4	Cell Signaling Technology	Cat# 4363; RRID:AB_2126429
Rabbit monoclonal anti-IRAK1 (D51G7)	Cell Signaling Technology	Cat# 4504; RRID:AB_1904032
Rabbit monoclonal anti-phospho-IRAK4 (Thr(P)-345/Ser(P)-346)	Pfizer (Vikram Rao)	
Camelid sdAb FluoTag®-X4 anti-GFP-Atto488	NanoTag Biotechnologies GmbH	Cat# N0304-At488;
goat anti-rabbit conjugated to Alexa Fluor 647	Invitrogen	Cat# A21246
goat anti-human conjugated to Alexa Fluor 647	Invitrogen	Cat# A21445
Nano-secondary AF568	Chromotek	Cat# srbAF568-1
Rabbit monoclonal anti-β-actin	Cell Signaling Technology	Cat# 4970; RRID:AB_2223172
Rabbit polyclonal anti-IRAK2	Cell Signaling Technology	Cat# 4367
Rabbit monoclonal anti-IRAKM [EPR26376]	Abcam	Cat#ab307708
Goat Anti-Rabbit HRP	Jackson Lab	Cat# 111-035-144
Goat Anti-Mouse HRP	Jackson Lab	Cat# 115-035-166
Rabbit Anti-Goat HRP	Jackson Lab	Cat# 305-036-045
Goat polyclonal anti-MyD88	R&D systems	Cat# AF3109 RRID:AB_2146703
Mouse monoclonal anti-RFP	Chromotek	Cat# 6G6
**Bacterial and Virus Strains**		
BL21 Rosetta	Novagen	Cat# 70954

NEB Stable	NEB	Cat# C3040
TOP10	Thermo	Cat# C404003
DH10EMBacY	Geneva Biotech	
**Chemicals, Peptides, and Recombinant Proteins**		
IRAK4 kinase inhibitor, PF-06650833	Sigma-Aldrich	Cat# PZ0327-5MG
His10-Halo-Tencon-SpycatcherV2-IL-1β	Deliz-Aguirre et al.[11]	
Puromycin dihydrochloride	Thermo	Cat# A1113803
Blasticidin	InvivoGen	Cat# ant-bl-05
Mouse IL-1β	Peprotech	Cat# 211-11B
JF646-HaloTag ligand	Luke Lavis, HHMI/Promega	Promega (GA1120)
InstantBlue Coomassie Protein Stain	Abcam	Cat#ab119211
4-(2-aminoethyl) benzene sulphonyl fluoride hydrochloride (AEBSF)	Apollo Scientific	Cat#BIMB2003
5-Bromo-4-chloro-3-indolyl-β-D-galactoside (X-Gal)	ForMedium	Cat#XGAL001
Isopropyl β-D-thiogalactoside (IPTG)	ForMedium	Cat#IPTG025
GSH 4B sepharose	GE Healthcare	Cat#17-0756-01
Dithiothreitol	Melford	Cat#D11000
Gentamycin sulphate	Melford	Cat#G0124
PreScission Protease (GST tagged)	MRC PPU Reagents	Cat#GNNYH4
Ni-NTA agarose	Qiagen	Cat#30210
4-(2-Hydroxyethyl) piperazine-1-ethanesulphonic acid (HEPES)	Sigma-Aldrich	Cat#H3375
Ampicillin sodium salt	Sigma-Aldrich	Cat#A9518
Benzamidine	Sigma-Aldrich	Cat#B6506
Imidazole	Sigma-Aldrich	Cat#56749
Kanamycin sulfate	Sigma-Aldrich	Cat#K1377
Magnesium chloride	Sigma-Aldrich	Cat#M1028
Tetracycline hydrochloride	Sigma-Aldrich	Cat#T7660

β-Mercaptoethanol	Sigma-Aldrich	Cat#M3148
Antibiotic / Antimycotic solution (100 x)	Thermo Fisher	Cat#15240062
Novex 10% Tris-Glycine Mini Gels	Thermo Fisher	Cat#XP00105Box
Novex Tris-Glycine Native Running Buffer (10×)	Thermo Fisher	Cat#LC2672
Novex Tris-Glycine Native Sample Buffer (2×)	Thermo Fisher	Cat#LC2673
Pierce Universal Nuclease	Thermo Fisher	Cat#88701
Sf-900 II SFM insect cell medium	Thermo Fisher	Cat#10902096
Glycerol	VWR	Cat#24388.320
**Critical Commercial Assays**		
MycoAlert® Mycoplasma Detection Kit	Lonza	Cat# LT07-218
QuickExtract DNA Extraction Solution	Lucigen	Cat# 101098
Mouse IL-2 DuoSet ELISA	R&D Systems	DY402
**Experimental Models: Cell Lines**		
Human: Gesicle producer 293T cell line	Clontech	Cat# 632617
Insect: *Spodoptera frugiperda* Sf21	Invitrogen	Cat#11497013
Mouse: EL4.NOB-1	ECACC	87020408
Mouse: EL4 MyD88-GFP	Deliz-Aguirre et al.[11]	
Mouse: EL4-MyD88-GFP/IRAK1-mScarlet	Deliz-Aguirre et al.[11]	
Mouse: EL4-MyD88-GFP/IRAK4-mScarlet	Deliz-Aguirre et al.[11]	
Mouse: EL4-MyD88-GFP/IRAK1-KO	Deliz-Aguirre et al.[11]	
Mouse: EL4-MyD88-GFP/IRAK4-KO	Deliz-Aguirre et al.[11]	
Mouse: EL4 MyD88/IRAK4/IRAK1-KO (3xKO)	This paper	
Mouse: EL4-MyD88-GFP/IRAK4-KO MmIRAK4-mScarlet	This paper	
Mouse: EL4-MyD88-GFP/IRAK4-KO + MmIRAK4-(K213/214A)-mScarlet	This paper	

Mouse: EL4-MyD88-GFP/IRAK4-KO MmIRAK4-(R12C)-mScarlet	This paper	
Mouse: EL4-MyD88-GFP/IRAK4-KO MmIRAK4-ΔDD-mScarlet	This paper	
Mouse: EL4-MyD88-GFP/IRAK4-KO MmIRAK4-ΔKD-mScarlet	This paper	
Mouse:EL4-MyD88-GFP/IRAK1-KO+ MmIRAK2-mScarlet-i	This paper	
Mouse:EL4-MyD88-GFP/IRAK1-KO+ MmIRAK3-mScarlet-i	This paper	
Mouse: EL4 3xKO + MmMyD88/MmIRAK4-GFP/MmIRAK1-mScarlet	This paper	
Mouse: EL4 3xKO + MmMyD88/MmIRAK4-(K213/214A)-GFP/MmIRAK1-mScarlet	This paper	
Mouse: EL4 3xKO + MmMyD88/MmIRAK4-(K213/214A)-GFP/MmIRAK2-mScarlet	This paper	
Mouse: EL4 3xKO + MmMyD88/MmIRAK4-(K213/214A)-GFP/MmIRAK3-mScarlet	This paper	
Mouse: EL4 3xKO + MmMyD88/MmIRAK4-ΔKD-GFP/MmIRAK1-mScarlet	This paper	
Mouse: EL4 3xKO + MmMyD88/MmIRAK4-ΔKD-GFP/MmIRAK2-mScarlet	This paper	
Mouse: EL4 3xKO + MmMyD88/MmIRAK4-ΔKD-GFP/MmIRAK3-mScarlet	This paper	
Mouse: EL4 3xKO + MmMyD88/MmIRAK4-GFP/MmIRAK1-ΔKD-mScarlet	This paper	
Mouse: EL4 3xKO + MmMyD88/MmIRAK4-GFP/MmIRAK2-ΔKD-mScarlet	This paper	

Mouse: EL4 3xKO + MmMyD88/MmIRAK4-GFP/MmIRAK3-ΔKD-mScarlet	This paper	
Mouse: EL4 3xKO + hMyD88/hIRAK4-GFP/hIRAK1-mScarlet	This paper	
Mouse: EL4 3xKO + MmMyD88/mMIRAK4-GFP/MmIRAK2-mScarlet	This paper	
Mouse: EL4 3xKO + MmMyD88/MmIRAK4-GFP/MmIRAK3-mScarlet	This paper	
Mouse: EL4 3xKO + hMyD88/hIRAK4 (D329A)-GFP/hIRAK1-mScarlet	This paper	
Mouse: EL4 3xKO + hMyD88/hIRAK4 (D329A/E loop mutant)-GFP/hIRAK1-mScarlet	This paper	
Mouse: EL4 3xKO + hMyD88/hIRAK4 (A loop mutant)-GFP/hIRAK1-mScarlet	This paper	
Mouse:EL4-MyD88-GFP/IRAK1-KO+ MmIRAK1-ΔDD-mScarlet-i	This paper	
Mouse:EL4-MyD88-GFP/IRAK1-KO+ MmIRAK2-ΔDD-mScarlet-i	This paper	
Mouse:EL4-MyD88-GFP/IRAK1-KO+ MmIRAK3-ΔDD-mScarlet-i	This paper	
Mouse: EL4 3xKO + MmMyD88/MmIRAK4-GFP/MmIRAK1-mScarlet	This paper	
Mouse: EL4 3xKO + MmMyD88/MmIRAK4 (A loop mutant)-GFP/MmIRAK1-mScarlet	This paper	
Mouse: EL4 3xKO + MmMyD88/MmIRAK4 (D329A)-GFP/MmIRAK1-mScarlet	This paper	
Mouse: EL4 3xKO + MmMyD88/MmIRAK4 (D329A/E loop mutant)-GFP/MmIRAK1-mScarlet	This paper	
Mouse: Hoxb8 B6 WT primary hematopoietic stem cells	Lichtenstein et al.[36]	
**Experimental Models: Organisms/strains**		
NA	NA	

**sgRNA sequences**		
MyD88, ENSMUST00000035092	5’-AAGCAGUGUCCCACAAACAA-3’	
IRAK4, ENSMUST00000074936	5’-UCUGGACCAGUAGAUCCACA-3’	
IRAK1, ENSMUST00000114354	5’-AAACAGGGAGUGGAACCUGG-3’	
**Recombinant DNA**		
pHR-dSV-MmIRAK4-mScarlet-i	Deliz-Aguirre et al. [11]	
pHR-dSV-MmIRAK4-(K213/214A)-mScarlet-i	This paper	
pHR-dSV-MmIRAK4-(R12C)-mScarlet-i	This paper	
pHR-dSV-MmIRAK4-ΔKD-mScarlet-i	This paper	
pHR-dSV-MmIRAK1-mScarlet-i	This paper	
pHR-dSV-MmIRAK4-mEGFP	This paper	
pHR-dSV-MmIRAK4-K213/214A-mEGFP	This paper	
pHR-dSV-MmIRAK4-ΔKD-mEGFP	This paper	
pHR-dSV-MmIRAK1-ΔKD-mScarlet-i	This paper	
pHR-dSV-MmIRAK2-ΔKD-mScarlet-i	This paper	
pHR-dSV-MmIRAK3-ΔKD-mScarlet-i	This paper	
pHR-dSV40-MmMyD88-3xFLAG-IRESpuro	This paper	
pHR-dSV40-MmMyD88-3xFLAG-IREStagBFP	This paper	
pHR-dSV40-MmIRAK2-mScarlet-i	This paper	
pHR-dSV40-MmIRAK3-mScarlet-i	This paper	
pHR-dSV40-MmIRAK4-ΔDD-mScarlet-i	This paper	
pHR-dSV40-MmIRAK1-ΔDD-mScarlet-i	This paper	
pHR-dSV40-MmIRAK2-ΔDD-mScarlet-i	This paper	
pHR-dSV40-hMyD88-3xFLAG-IREStagBFP	This paper	

pHR-dSV40-hIRAK4-mEGFP	This paper	
pHR-dSV40-hIRAK1-mScarlet-i	This paper	
pHR-dSV40-hIRAK4-D329A-mEGFP	This paper	
pHR-dSV40-hIRAK4-D329A-E-loop-mEGFP	This paper	
pHR-dSV40-hIRAK4-A-loop-mEGFP	This paper	
pHR-dSV40-MmIRAK4-D329A-mEGFP	This paper	
pHR-dSV40-MmIRAK4-D329A-E-loop-mEGFP	This paper	
pHR-dSV40-MmIRAK4-A-loop-mEGFP	This paper	
pFastBac-6His-TEV-GST-3C-AVI-IRAK1(187-527; D358A)	MRC PPU Reagents Lange et al.[19]	DU56583
pFastBac-6His-TEV-GST-3C-IRAK4(160-460)	MRC PPU Reagents	DU65498
pFBDM-GST-3C-IRAK4(160-460; T342E/T345E/S346E)	MRC PPU Reagents	DU66477
pFBDM-GST-3C-IRAK4(160-460; T342A/T345A/S346A)	MRC PPU Reagents	DU66476
pFastBac-6His-TEV-GST-3C-IRAK4(160-460; D329A)	MRC PPU Reagents Lange et al.[19]	DU56578
pFastBac-6His-TEV-GST-3C-AVI-IRAK4(160-460; D329A/T342E/T345E/S346E)	MRC PPU Reagents	DU80380
pFastBac-6His-TEV-GST-3C-AVI-IRAK4(160-460; D329A/T342A/T345A/S346A)	MRC PPU Reagents	DU76427
**Software and Algorithms**		
Fiji	NIH	https://fiji.sc/
R	CRAN	https://cran.r-project.org/
AF3	Abramson et al. 2024[15]	https://alphafoldserver.com/
GraphPad Prism v10.5	GraphPad	https://www.graphpad.com/
ChimeraX	UCSF Resource for Biocomputing, Visualization, and Informatics	https://cgl.ucsf.edu/chimerax

Image Lab	Bio-Rad	www.bio-rad.com/product/image-lab-software
Fiji/Python/R scripts from image analysis	This study	https://github.com/MJ-Taylor-Lab/IRAK4_Autophosphorylaytion_Paper.git

Supplementary Table 2. Protein sequences of IRAK1/2/3/4 mScarlet/mEGFP fusion constructs

Death Domain (DD), Kinase Domain (KD), GGS linker, mScarlet-i ORF, mEGFP point mutations underlined

**Mouse IRAK4 Wild Type mScarlet**
MNKPLTPSTYIRNLNVGILRKLSDFIDPQEGWKKLAVAIKKPSGDDRYNQFHIRRFEALLQTGK<br>SPTCELLFDWGTTNCTVGDLVDLLVQIELFAPATLLLPDAVPQTVKSLPPREAATVAQTHGPC<br>QEKDRTSVMPMPKLEHSCEPPDSSSPDNRSVESSDTRFHSFSFHELKSITNNFDEQPASAG<br>GNRMGEGGFGVVYKGCVNNTIVAVKKLGAMVEISTEELKQQFDQEIKVMATCQHENLVELLG<br>FSSDSDNLCLVYAYMPNGSLLDRLSCLDGTPPLSWHTRCKVAQGTANGIRFLHENHHIHRDIK<br>SANILLDRDFTAKISDFGLARASARLAQTVMTSRIVGTTAYMAPEALRGEITPKSDIYSFGVVLL<br>ELITGLAAVDENREPQLLLDIKEEIEDEEKTIEDYTDEKMSDADPASVEAMYSAASQCLHEKKN<br>RRPDIAKVQQLLQEMSAGGSGGSGGSVSKGEAVIKEFMRFKVHMEGSMNGHEFEIEGEGE<br>GRPYEGTQTAKLKVTKGGPLPFSWDILSPQFMYGSRAFIKHPADIPDYYKQSFPEGFKWERV<br>MNFEDGGAVTVTQDTSLEDGTLIYKVKLRGTNFPPDGPVMQKKTMGWEASTERLYPEDGVL<br>KGDIKMALRLKDGGRYLADFKTTYKAKKPVQMPGAYNVDRKLDITSHNEDYTVVEQYERSEG<br>RHSTGGMDELYK
**Mouse IRAK4 Wild Type mEGFP**
MNKPLTPSTYIRNLNVGILRKLSDFIDPQEGWKKLAVAIKKPSGDDRYNQFHIRRFEALLQTGK<br>SPTCELLFDWGTTNCTVGDLVDLLVQIELFAPATLLLPDAVPQTVKSLPPREAATVAQTHGPC<br>QEKDRTSVMPMPKLEHSCEPPDSSSPDNRSVESSDTRFHSFSFHELKSITNNFDEQPASAG<br>GNRMGEGGFGVVYKGCVNNTIVAVKKLGAMVEISTEELKQQFDQEIKVMATCQHENLVELLG<br>FSSDSDNLCLVYAYMPNGSLLDRLSCLDGTPPLSWHTRCKVAQGTANGIRFLHENHHIHRDIK<br>SANILLDRDFTAKISDFGLARASARLAQTVMTSRIVGTTAYMAPEALRGEITPKSDIYSFGVVLL<br>ELITGLAAVDENREPQLLLDIKEEIEDEEKTIEDYTDEKMSDADPASVEAMYSAASQCLHEKKN<br>RRPDIAKVQQLLQEMSAGGSGGSGGSDPPVATMVSKGEELFTGVVPILVELDGDVNGHKFS<br>VSGEGEGDATYGKLTLKFICTTGKLPVPWPTLVTTLTYGVQCFSRYPDHMKQHDFFKSAMPE<br>GYVQERTIFFKDDGNYKTRAEVKFEGDTLVNRIELKGIDFKEDGNILGHKLEYNYNSHNVYIM<br>ADKQKNGIKVNFKIRHNIEDGSVQLADHYQQNTPIGDGPVLLPDNHYLSTQSKLSKDPNEKR<br>DHMVLLEFVTAAGITLGMDELYK
**Mouse IRAK4 Kinase Dead (K213/214A) mScarlet**
MNKPLTPSTYIRNLNVGILRKLSDFIDPQEGWKKLAVAIKKPSGDDRYNQFHIRRFEALLQTGK<br>SPTCELLFDWGTTNCTVGDLVDLLVQIELFAPATLLLPDAVPQTVKSLPPREAATVAQTHGPC<br>QEKDRTSVMPMPKLEHSCEPPDSSSPDNRSVESSDTRFHSFSFHELKSITNNFDEQPASAG<br>GNRMGEGGFGVVYKGCVNNTIVAVAALGAMVEISTEELKQQFDQEIKVMATCQHENLVELLG<br>FSSDSDNLCLVYAYMPNGSLLDRLSCLDGTPPLSWHTRCKVAQGTANGIRFLHENHHIHRDIK<br>SANILLDRDFTAKISDFGLARASARLAQTVMTSRIVGTTAYMAPEALRGEITPKSDIYSFGVVLL<br>ELITGLAAVDENREPQLLLDIKEEIEDEEKTIEDYTDEKMSDADPASVEAMYSAASQCLHEKKN<br>RRPDIAKVQQLLQEMSAGGSGGSGGSVSKGEAVIKEFMRFKVHMEGSMNGHEFEIEGEGE<br>GRPYEGTQTAKLKVTKGGPLPFSWDILSPQFMYGSRAFIKHPADIPDYYKQSFPEGFKWERV<br>MNFEDGGAVTVTQDTSLEDGTLIYKVKLRGTNFPPDGPVMQKKTMGWEASTERLYPEDGVL<br>KGDIKMALRLKDGGRYLADFKTTYKAKKPVQMPGAYNVDRKLDITSHNEDYTVVEQYERSEG<br>RHSTGGMDELYK

**Mouse IRAK4 Kinase Dead (K213/214A) mEGFP**
MNKPLTPSTYIRNLNVGILRKLSDFIDPQEGWKKLAVAIKKPSGDDRYNQFHIRRFEALLQTGK<br>SPTCELLFDWGTTNCTVGDLVDLLVQIELFAPATLLLPDAVPQTVKSLPPREAATVAQTHGPC<br>QEKDRTSVMPMPKLEHSCEPPDSSSPDNRSVESSDTRFHSFSFHELKSITNNFDEQPASAG<br>GNRMGEGGFGVVYKGCVNNTIVAVAALGAMVEISTEELKQQFDQEIKVMATCQHENLVELLG<br>FSSDSDNLCLVYAYMPNGSLLDRLSCLDGTPPLSWHTRCKVAQGTANGIRFLHENHHIHRDIK<br>SANILLDRDFTAKISDFGLARASARLAQTVMTSRIVGTTAYMAPEALRGEITPKSDIYSFGVVLL<br>ELITGLAAVDENREPQLLLDIKEEIEDEEKTIEDYTDEKMSDADPASVEAMYSAASQCLHEKKN<br>RRPDIAKVQQLLQEMSAGGSGGSGGSDPPVATMVSKGEELFTGVVPILVELDGDVNGHKFS<br>VSGEGEGDATYGKLTLKFICTTGKLPVPWPTLVTTLTYGVQCFSRYPDHMKQHDFFKSAMPE<br>GYVQERTIFFKDDGNYKTRAEVKFEGDTLVNRIELKGIDFKEDGNILGHKLEYNYNSHNVYIM<br>ADKQKNGIKVNFKIRHNIEDGSVQLADHYQQNTPIGDGPVLLPDNHYLSTQSKLSKDPNEKR<br>DHMVLLEFVTAAGITLGMDELYK
**Mouse IRAK4-ΔKD-mScarlet**
MNKPLTPSTYIRNLNVGILRKLSDFIDPQEGWKKLAVAIKKPSGDDRYNQFHIRRFEALLQTGK<br>SPTCELLFDWGTTNCTVGDLVDLLVQIELFAPATLLLPDAGGSGGSNRRPDIAKVQQLLQEMS<br>AGGSGGSGGSVSKGEAVIKEFMRFKVHMEGSMNGHEFEIEGEGEGRPYEGTQTAKLKVTK<br>GGPLPFSWDILSPQFMYGSRAFIKHPADIPDYYKQSFPEGFKWERVMNFEDGGAVTVTQDT<br>SLEDGTLIYKVKLRGTNFPPDGPVMQKKTMGWEASTERLYPEDGVLKGDIKMALRLKDGGR<br>YLADFKTTYKAKKPVQMPGAYNVDRKLDITSHNEDYTVVEQYERSEGRHSTGGMDELYK
**Mouse IRAK4-ΔKD-mEGFP**
MNKPLTPSTYIRNLNVGILRKLSDFIDPQEGWKKLAVAIKKPSGDDRYNQFHIRRFEALLQTGK<br>SPTCELLFDWGTTNCTVGDLVDLLVQIELFAPATLLLPDAGGSGGSNRRPDIAKVQQLLQEMS<br>AGGSGGSGGSDPPVATMVSKGEELFTGVVPILVELDGDVNGHKFSVSGEGEGDATYGKLTL<br>KFICTTGKLPVPWPTLVTTLTYGVQCFSRYPDHMKQHDFFKSAMPEGYVQERTIFFKDDGNY<br>KTRAEVKFEGDTLVNRIELKGIDFKEDGNILGHKLEYNYNSHNVYIMADKQKNGIKVNFKIRHN<br>IEDGSVQLADHYQQNTPIGDGPVLLPDNHYLSTQSKLSKDPNEKRDHMVLLEFVTAAGITLG<br>MDELYK
**Mouse IRAK4 (R12C) mScarlet**
MNKPLTPSTYICNLNVGILRKLSDFIDPQEGWKKLAVAIKKPSGDDRYNQFHIRRFEALLQTGK<br>SPTCELLFDWGTTNCTVGDLVDLLVQIELFAPATLLLPDAVPQTVKSLPPREAATVAQTHGPC<br>QEKDRTSVMPMPKLEHSCEPPDSSSPDNRSVESSDTRFHSFSFHELKSITNNFDEQPASAG<br>GNRMGEGGFGVVYKGCVNNTIVAVKKLGAMVEISTEELKQQFDQEIKVMATCQHENLVELLG<br>FSSDSDNLCLVYAYMPNGSLLDRLSCLDGTPPLSWHTRCKVAQGTANGIRFLHENHHIHRDIK<br>SANILLDRDFTAKISDFGLARASARLAQTVMTSRIVGTTAYMAPEALRGEITPKSDIYSFGVVLL<br>ELITGLAAVDENREPQLLLDIKEEIEDEEKTIEDYTDEKMSDADPASVEAMYSAASQCLHEKKN<br>RRPDIAKVQQLLQEMSAGGSGGSGGSVSKGEAVIKEFMRFKVHMEGSMNGHEFEIEGEGE<br>GRPYEGTQTAKLKVTKGGPLPFSWDILSPQFMYGSRAFIKHPADIPDYYKQSFPEGFKWERV<br>MNFEDGGAVTVTQDTSLEDGTLIYKVKLRGTNFPPDGPVMQKKTMGWEASTERLYPEDGVL<br>KGDIKMALRLKDGGRYLADFKTTYKAKKPVQMPGAYNVDRKLDITSHNEDYTVVEQYERSEG<br>RHSTGGMDELYK
**Mouse IRAK4-ΔDD-mScarlet**

MFHELKSITNNFDEQPASAGGNRMGEGGFGVVYKGCVNNTIVAVKKLGAMVEISTEELKQQF
DQEIKVMATCQHENLVELLGFSSDSDNLCLVYAYMPNGSLLDRLSCLDGTPPLSWHTRCKVA
QGTANGIRFLHENHHIHRDIKSANILLDRDFTAKISDFGLARASARLAQTVMTSRIVGTTAYMAP
EALRGEITPKSDIYSFGVVLLELITGLAAVDENREPQLLLDIKEEIEDEEKTIEDYTDEKMSDAD
PASVEAMYSAASQCLHEKKNRRPDIAKVQQLLQEMSAGGSGGSGGSVSKGEAVIKEFMRFK
VHMEGSMNGHEFEIEGEGEGRPYEGTQTAKLKVTKGGPLPFSWDILSPQFMYGSRAFIKHP
ADIPDYYKQSFPEGFKWERVMNFEDGGAVTVTQDTSLEDGTLIYKVKLRGTNFPPDGPVMQ
KKTMGWEASTERLYPEDGVLKGDIKMALRLKDGGRYLADFKTTYKAKKPVQMPGAYNVDRK
LDITSHNEDYTVVEQYERSEGRHSTGGMDELYK

Mouse IRAK4-D329A-mEGFP

MNKPLTPSTYIRNLNVGILRKLSDFIDPQEGWKKLAVAIKKPSGDDRYNQFHIRRFEALLQTGK
SPTCELLFDWGTTNCTVGDLVDLLVQIELFAPATLLLPDAVPQTVKSLPPREAATVAQTHGPC
QEKDRTSVMPMPKLEHSCEPPDSSSPDNRSVESSDTRFHSFSFHELKSITNNFDEQPASAG
GNRMGEGGFGVVYKGCVNNTIVAVKKLGAMVEISTEELKQQFDQEIKVMATCQHENLVELLG
FSSDSDNLCLVYAYMPNGSLLDRLSCLDGTPPLSWHTRCKVAQGTANGIRFLHENHHIHRDIK
SANILLDRDFTAKISAFGLARASARLAQTVMTSRIVGTTAYMAPEALRGEITPKSDIYSFGVVLL
ELITGLAAVDENREPQLLLDIKEEIEDEEKTIEDYTDEKMSDADPASVEAMYSAASQCLHEKKN
RRPDIAKVQQLLQEMSAGGSGGSGGSDPPVATMVSKGEELFTGVVPILVELDGDVNGHKFS
VSGEGEGDATYGKLTLKFICTTGKLPVPWPTLVTTLTYGVQCFSRYPDHMKQHDFFKSAMPE
GYVQERTIFFKDDGNYKTRAEVKFEGDTLVNRIELKGIDFKEDGNILGHKLEYNYNSHNVYIM
ADKQKNGIKVNFKIRHNIEDGSVQLADHYQQNTPIGDGPVLLPDNHYLSTQSKLSKDPNEKR
DHMVLLEFVTAAGITLGMDELYK

Mouse IRAK4-D329A-E-loop-mEGFP

MNKPLTPSTYIRNLNVGILRKLSDFIDPQEGWKKLAVAIKKPSGDDRYNQFHIRRFEALLQTGK
SPTCELLFDWGTTNCTVGDLVDLLVQIELFAPATLLLPDAVPQTVKSLPPREAATVAQTHGPC
QEKDRTSVMPMPKLEHSCEPPDSSSPDNRSVESSDTRFHSFSFHELKSITNNFDEQPASAG
GNRMGEGGFGVVYKGCVNNTIVAVKKLGAMVEISTEELKQQFDQEIKVMATCQHENLVELLG
FSSDSDNLCLVYAYMPNGSLLDRLSCLDGTPPLSWHTRCKVAQGTANGIRFLHENHHIHRDIK
SANILLDRDFTAKISAFGLARASARLAQEVMEERIVGTTAYMAPEALRGEITPKSDIYSFGVVLL
ELITGLAAVDENREPQLLLDIKEEIEDEEKTIEDYTDEKMSDADPASVEAMYSAASQCLHEKKN
RRPDIAKVQQLLQEMSASSGSGGSGGSGGSDPPVATMVSKGEELFTGVVPILVELDGDVNG
HKFSVSGEGEGDATYGKLTLKFICTTGKLPVPWPTLVTTLTYGVQCFSRYPDHMKQHDFFKS
AMPEGYVQERTIFFKDDGNYKTRAEVKFEGDTLVNRIELKGIDFKEDGNILGHKLEYNYNSHN
VYIMADKQKNGIKVNFKIRHNIEDGSVQLADHYQQNTPIGDGPVLLPDNHYLSTQSKLSKDPN
EKRDHMVLLEFVTAAGITLGMDELYK

Mouse IRAK4-A-loop-mEGFP

MNKPLTPSTYIRNLNVGILRKLSDFIDPQEGWKKLAVAIKKPSGDDRYNQFHIRRFEALLQTGK
SPTCELLFDWGTTNCTVGDLVDLLVQIELFAPATLLLPDAVPQTVKSLPPREAATVAQTHGPC
QEKDRTSVMPMPKLEHSCEPPDSSSPDNRSVESSDTRFHSFSFHELKSITNNFDEQPASAG
GNRMGEGGFGVVYKGCVNNTIVAVKKLGAMVEISTEELKQQFDQEIKVMATCQHENLVELLG
FSSDSDNLCLVYAYMPNGSLLDRLSCLDGTPPLSWHTRCKVAQGTANGIRFLHENHHIHRDIK
SANILLDRDFTAKISDFGLARASARLAQAVMAARIVGTTAYMAPEALRGEITPKSDIYSFGVVLL
ELITGLAAVDENREPQLLLDIKEEIEDEEKTIEDYTDEKMSDADPASVEAMYSAASQCLHEKKN
RRPDIAKVQQLLQEMSAGGSGGSGGSDPPVATMVSKGEELFTGVVPILVELDGDVNGHKFS

VSGEGEGDATYGKLTLKFICTTGKLPVPWPTLVTTLTYGVQCFSRYPDHMKQHDFFKSAMPE<br>GYVQERTIFFKDDGNYKTRAEVKFEGDTLVNRIELKGIDFKEDGNILGHKLEYNYNSHNVYIM<br>ADKQKNGIKVNFKIRHNIEDGSVQLADHYQQNTPIGDGPVLLPDNHYLSTQSKLSKDPNEKR<br>DHMVLLEFVTAAGITLGMDELYK
**Mouse IRAK1-mScarlet**
MAGGPGPGEPVVPGAQHFLYEVPPWVMCRFYKVMDALEPADWCQFAALIVRDQTELRLCE<br>RSEQRTASVLWPWINRNARVADLVHILTHLQLLRARDIITAWHPPAPVVPPSTAAPRPSSISAG<br>SEAGDWSPRKLQSSASTFLSPAFPGSQTHSESELLQVPLPVSLGPPLPSSAPSSTKSSPESP<br>VSGLQRAHPSPFCWPFCEISQGTCNFSEELRIGEGGFGCVYRAVMRNTTYAVKRLKEEADLE<br>WTMVKQSFLTEVEQLSRFRHPNIVDFAGYCAESGLYCLVYGFLPNGSLEDQLHLQTQACSPL<br>SWPQRLDILLGTARAIQFLHQDSPSLIHGDIKSSNVLLDERLMPKLGDFGLARFSRFAGAKAS<br>QSSTVARTSTVRGTLAYLPEEYIKTGRLAVDTDTFSFGVVILETLAGQRAVRTQGAKTKYLKDL<br>IEDEAEEAGVTLKSTQPTLWVGVATDAWAAPIAAQIYKKHLDSRPGPCPPQLGLALAQLACCC<br>MHRRAKKRPPMTQVYKRLEGLQAGPPWELEVAGHGSPSPQENSYMSTTGSAQSGDEPWQ<br>PLVVTTRAPAQAAQQLQRSPNQPVESDESVPGLSATLHSWHLTPGSHPSPASFREASCTQG<br>GTTRESSVRSSPGFQPTTMEGSPTGSSSLLSSEPPQIIINPARQKMVQKLALYEEGVLDSLQL<br>LSSGFFPGLDLEPEKSQGPEESDEFQSGSGGSGGSGGSVSKGEAVIKEFMRFKVHMEGSM<br>NGHEFEIEGEGEGRPYEGTQTAKLKVTKGGPLPFSWDILSPQFMYGSRAFIKHPADIPDYYK<br>QSFPEGFKWERVMNFEDGGAVTVTQDTSLEDGTLIYKVKLRGTNFPPDGPVMQKKTMGWE<br>ASTERLYPEDGVLKGDIKMALRLKDGGRYLADFKTTYKAKKPVQMPGAYNVDRKLDITSHNE<br>DYTVVEQYERSEGRHSTGGMDELYK
**Mouse IRAK1-ΔKD-mScarlet**
MAGGPGPGEPVVPGAQHFLYEVPPWVMCRFYKVMDALEPADWCQFAALIVRDQTELRLCE<br>RSEQRTASVLWPWINRNARVADLVHILTHLQLLRARDIITAWHPPAPVVSGSGGSGGSGGSVS<br>KGEAVIKEFMRFKVHMEGSMNGHEFEIEGEGEGRPYEGTQTAKLKVTKGGPLPFSWDILSP<br>QFMYGSRAFIKHPADIPDYYKQSFPEGFKWERVMNFEDGGAVTVTQDTSLEDGTLIYKVKLR<br>GTNFPPDGPVMQKKTMGWEASTERLYPEDGVLKGDIKMALRLKDGGRYLADFKTTYKAKKP<br>VQMPGAYNVDRKLDITSHNEDYTVVEQYERSEGRHSTGGMDELYK
**Mouse IRAK1-ΔDD-mScarlet**
MAWHPPAPVVPPSTAAPRPSSISAGSEAGDWSPRKLQSSASTFLSPAFPGSQTHSESELLQ<br>VPLPVSLGPPLPSSAPSSTKSSPESPVSGLQRAHPSPFCWPFCEISQGTCNFSEELRIGEGG<br>FGCVYRAVMRNTTYAVKRLKEEADLEWTMVKQSFLTEVEQLSRFRHPNIVDFAGYCAESGLY<br>CLVYGFLPNGSLEDQLHLQTQACSPLSWPQRLDILLGTARAIQFLHQDSPSLIHGDIKSSNVLL<br>DERLMPKLGDFGLARFSRFAGAKASQSSTVARTSTVRGTLAYLPEEYIKTGRLAVDTDTFSFG<br>VVILETLAGQRAVRTQGAKTKYLKDLIEDEAEEAGVTLKSTQPTLWVGVATDAWAAPIAAQIYK<br>KHLDSRPGPCPPQLGLALAQLACCCMHRRAKKRPPMTQVYKRLEGLQAGPPWELEVAGHG<br>SPSPQENSYMSTTGSAQSGDEPWQPLVVTTRAPAQAAQQLQRSPNQPVESDESVPGLSAT<br>LHSWHLTPGSHPSPASFREASCTQGGTTRESSVRSSPGFQPTTMEGSPTGSSSLLSSEPPQ<br>IIINPARQKMVQKLALYEEGVLDSLQLLSSGFFPGLDLEPEKSQGPEESDEFQSGSGGSGGS<br>GGSVSKGEAVIKEFMRFKVHMEGSMNGHEFEIEGEGEGRPYEGTQTAKLKVTKGGPLPFSW<br>DILSPQFMYGSRAFIKHPADIPDYYKQSFPEGFKWERVMNFEDGGAVTVTQDTSLEDGTLIYK<br>VKLRGTNFPPDGPVMQKKTMGWEASTERLYPEDGVLKGDIKMALRLKDGGRYLADFKTTYK<br>AKKPVQMPGAYNVDRKLDITSHNEDYTVVEQYERSEGRHSTGGMDELYK
**Mouse IRAK2-mScarlet**

MACYIYQLPSWVLDDLCRNIDTLSEWDWMQFASYVITDLTQLRKIKSMERVQGVSITRELLW<br>WWSMRQATVQQLVDLLCHLELYRAAQIVLSWKPVPESTSPLPAFPEAVKPGAVATSRRNLKD<br>EQEKVRPVKPRSLLDTGPIMAGAQRQRPCEMDAPCSLKTDAPDSPQSKYCSTSTSAPKQER<br>LLGLPGDRLFWSEADVVQATEDFDQSHRISEGTFADIYQGQRNGVAFAFKKLREVAGSSPGS<br>MDRFLQAEMQLCLRCCHANVLPLLGFCTGRQFHSLIYPYMANGSLHDRLWAQGNSDMLPW<br>PQRASICSGLLLAVEHLHSLDIIHSNVKSANVLLDQHLNPKLAHPVAHPHPDNKKTKYTVMRT<br>HLFQASAAYLPEHFIRVGQLTKQVDIFSCGIVLAEVLTGIPAMDKDRSPVYLKDLLLSEIPNSTS<br>SVCSRKTSMGKAVVKEICQRHVEKRAGLLPEACEEAWATAVSVCLRRRNASVEEARVSLAG<br>VEEQLRGQLSLPWSRVSEATGSSSNTPEETDDVDNSSLSVPSLVMMASCPGAASSPLFTGH<br>GAAQPSTSGRQEADSSSEACTGPQTPQNATETSWKIEINEAKRRLMENIVLYKEERLDSSEL<br>FGPSGSGGSGGSGGSVSKGEAVIKEFMRFKVHMEGSMNGHEFEIEGEGEGRPYEGTQTAK<br>LKVTKGGPLPFSWDILSPQFMYGSRAFIKHPADIPDYYKQSFPEGFKWERVMNFEDGGAVTV<br>TQDTSLEDGTLIYKVKLRGTNFPPDGPVMQKKTMGWEASTERLYPEDGVLKGDIKMALRLKD<br>GGRYLADFKTTYKAKKPVQMPGAYNVDRKLDITSHNEDYTVVEQYERSEGRHSTGGMDELY<br>K
**Mouse IRAK2-ΔKD-mScarlet**
MACYIYQLPSWVLDDLCRNIDTLSEWDWMQFASYVITDLTQLRKIKSMERVQGVSITRELLW<br>WWSMRQATVQQLVDLLCHLELYRAAQIVLSWKPVPESTSPLPAFPEAVGSGGSGGSGGSVS<br>KGEAVIKEFMRFKVHMEGSMNGHEFEIEGEGEGRPYEGTQTAKLKVTKGGPLPFSWDILSP<br>QFMYGSRAFIKHPADIPDYYKQSFPEGFKWERVMNFEDGGAVTVTQDTSLEDGTLIYKVKLR<br>GTNFPPDGPVMQKKTMGWEASTERLYPEDGVLKGDIKMALRLKDGGRYLADFKTTYKAKKP<br>VQMPGAYNVDRKLDITSHNEDYTVVEQYERSEGRHSTGGMDELYK
**Mouse IRAK2-ΔDD-mScarlet**
MPVPESTSPLPAFPEAVKPGAVATSRRNLKDEQEKVRPVKPRSLLDTGPIMAGAQRQRPCE<br>MDAPCSLKTDAPDSPQSKYCSTSTSAPKQERLLGLPGDRLFWSEADVVQATEDFDQSHRIS<br>EGTFADIYQGQRNGVAFAFKKLREVAGSSPGSMDRFLQAEMQLCLRCCHANVLPLLGFCTG<br>RQFHSLIYPYMANGSLHDRLWAQGNSDMLPWPQRASICSGLLLAVEHLHSLDIIHSNVKSAN<br>VLLDQHLNPKLAHPVAHPHPDNKKTKYTVMRTHLFQASAAYLPEHFIRVGQLTKQVDIFSCGI<br>VLAEVLTGIPAMDKDRSPVYLKDLLLSEIPNSTSSVCSRKTSMGKAVVKEICQRHVEKRAGLL<br>PEACEEAWATAVSVCLRRRNASVEEARVSLAGVEEQLRGQLSLPWSRVSEATGSSSNTPEE<br>TDDVDNSSLSVPSLVMMASCPGAASSPLFTGHGAAQPSTSGRQEADSSSEACTGPQTPQN<br>ATETSWKIEINEAKRRLMENIVLYKEERLDSSELFGPSGSGGSGGSGGSVSKGEAVIKEFMRF<br>KVHMEGSMNGHEFEIEGEGEGRPYEGTQTAKLKVTKGGPLPFSWDILSPQFMYGSRAFIKH<br>PADIPDYYKQSFPEGFKWERVMNFEDGGAVTVTQDTSLEDGTLIYKVKLRGTNFPPDGPVM<br>QKKTMGWEASTERLYPEDGVLKGDIKMALRLKDGGRYLADFKTTYKAKKPVQMPGAYNVDR<br>KLDITSHNEDYTVVEQYERSEGRHSTGGMDELYK
**Mouse IRAK3-mScarlet**
MAGRCGARGALSPQLLLFDLPPALLGELCGILDSCDGPLGWRGLAERLSNSWLDVRHIEKYV<br>NQGKSGTRELLWSWAQKNKTIGDLLEVLQDMGHQRAIHLIINYGVSWTPSVQTHHELPFPSF<br>PPEVKHACRENDPGPLEPANVTVDNVLVPEHNEKGTLQKTPISFQSILEGTKHFHKDFLIGEG<br>EIFEVYRVDIRNQAYAVKLFKQEKKMQLKKHWKRFLSELEVLLLFRHPHILELAAYFTETEKLCL<br>VYPYMSNGTLFDRLQCTNGTTPLSWHVRISVLIGIAKAIQYLHNTQPCAVICGNVSSANILLDD<br>QLQPKLTDFAAAHFRPNLEQQSSTINMTGGGRKHLWYMPEEYIRQGRLSVKTDVYSFGIVIM<br>EVLTGCKVVLDDPKHVQLRDLLMELMEKRGLDSCLSFLDRKIPPCPRNFSAKLFSLAGRCVAT

KAKLRPTMDEVLSSLESTQPSLYFAEDPPTSLKSFRCPSPLFLDNVPSIPVEDDENQNNHSVP<br>PKEVLGTDRVTQKTPFECSQSEVTFLGLDRNRGNRGSEADCNVPSSSHEECWSPELVAPSQ<br>DLSPTVISLGSSWEVPGHSYGSKPMEKRCSSGLFCSEHEQSKKQGSGGSGGSGGSVSKGE<br>AVIKEFMRFKVHMEGSMNGHEFEIEGEGEGRPYEGTQTAKLKVTKGGPLPFSWDILSPQFM<br>YGSRAFIKHPADIPDYYKQSFPEGFKWERVMNFEDGGAVTVTQDTSLEDGTLIYKVKLRGTN<br>FPPDGPVMQKKTMGWEASTERLYPEDGVLKGDIKMALRLKDGGRYLADFKTTYKAKKPVQM<br>PGAYNVDRKLDITSHNEDYTVVEQYERSEGRHSTGGMDELYK
**Mouse IRAK3-ΔKD-mScarlet**
MAGRCGARGALSPQLLLFDLPPALLGELCGILDSCDGPLGWRGLAERLSNSWLDVRHIEKYV<br>NQGKSGTRELLWSWAQKNKTIGDLLEVLQDMGHQRAIHLIINYGVSWTGSGGSGGSGGSVS<br>KGEAVIKEFMRFKVHMEGSMNGHEFEIEGEGEGRPYEGTQTAKLKVTKGGPLPFSWDILSP<br>QFMYGSRAFIKHPADIPDYYKQSFPEGFKWERVMNFEDGGAVTVTQDTSLEDGTLIYKVKLR<br>GTNFPPDGPVMQKKTMGWEASTERLYPEDGVLKGDIKMALRLKDGGRYLADFKTTYKAKKP<br>VQMPGAYNVDRKLDITSHNEDYTVVEQYERSEGRHSTGGMDELYK
**Mouse IRAK3-ΔDD-mScarlet**
MNYGVSWTPSVQTHHELPFPSFPPEVKHACRENDPGPLEPANVTVDNVLVPEHNEKGTLQK<br>TPISFQSILEGTKHFHKDFLIGEGEIFEVYRVDIRNQAYAVKLFKQEKKMQLKKHWKRFLSELE<br>VLLLFRHPHILELAAYFTETEKLCLVYPYMSNGTLFDRLQCTNGTTPLSWHVRISVLIGIAKAIQ<br>YLHNTQPCAVICGNVSSANILLDDQLQPKLTDFAAAHFRPNLEQQSSTINMTGGGRKHLWYM<br>PEEYIRQGRLSVKTDVYSFGIVIMEVLTGCKVVLDDPKHVQLRDLLMELMEKRGLDSCLSFLD<br>RKIPPCPRNFSAKLFSLAGRCVATKAKLRPTMDEVLSSLESTQPSLYFAEDPPTSLKSFRCPS<br>PLFLDNVPSIPVEDDENQNNHSVPPKEVLGTDRVTQKTPFECSQSEVTFLGLDRNRGNRGS<br>EADCNVPSSSHEECWSPELVAPSQDLSPTVISLGSSWEVPGHSYGSKPMEKRCSSGLFCSE<br>HEQSKKQSGSGGSGGSGGSVSKGEAVIKEFMRFKVHMEGSMNGHEFEIEGEGEGRPYEGT<br>QTAKLKVTKGGPLPFSWDILSPQFMYGSRAFIKHPADIPDYYKQSFPEGFKWERVMNFEDGG<br>AVTVTQDTSLEDGTLIYKVKLRGTNFPPDGPVMQKKTMGWEASTERLYPEDGVLKGDIKMAL<br>RLKDGGRYLADFKTTYKAKKPVQMPGAYNVDRKLDITSHNEDYTVVEQYERSEGRHSTGGM<br>DELYK
**Human IRAK4 Wild Type-mEGFP**
MNKPITPSTYVRCLNVGLIRKLSDFIDPQEGWKKLAVAIKKPSGDDRYNQFHIRRFEALLQTGK<br>SPTSELLFDWGTTNCTVGDLVDLLIQNEFFAPASLLLPDAVPKTANTLPSKEAITVQQKQMPFC<br>DKDRTLMTPVQNLEQSYMPPDSSSPENKSLEVSDTRFHSFSFYELKNVTNNFDERPISVGG<br>NKMGEGGFGVVYKGYVNNTTVAVKKLAAMVDITTEELKQQFDQEIKVMAKCQHENLVELLGF<br>SSDGDDLCLVYVYMPNGSLLDRLSCLDGTPPLSWHMRCKIAQGAANGINFLHENHHIHRDIK<br>SANILLDEAFTAKISDFGLARASEKFAQTVMTSRIVGTTAYMAPEALRGEITPKSDIYSFGVVLL<br>EIITGLPAVDEHREPQLLLDIKEEIEDEEKTIEDYIDKKMNDADSTSVEAMYSVASQCLHEKKNK<br>RPDIKKVQQLLQEMTASSGSGGSGGSGGSDPPVATMVSKGEELFTGVVPILVELDGDVNGH<br>KFSVSGEGEGDATYGKLTLKFICTTGKLPVPWPTLVTTLTYGVQCFSRYPDHMKQHDFFKSA<br>MPEGYVQERTIFFKDDGNYKTRAEVKFEGDTLVNRIELKGIDFKEDGNILGHKLEYNYNSHNV<br>YIMADKQKNGIKVNFKIRHNIEDGSVQLADHYQQNTPIGDGPVLLPDNHYLSTQSKLSKDPNE<br>KRDHMVLLEFVTAAGITLGMDELYK
**Human IRAK4-D329A-mEGFP**
MNKPITPSTYVRCLNVGLIRKLSDFIDPQEGWKKLAVAIKKPSGDDRYNQFHIRRFEALLQTGK

SPTSELLFDWGTTNCTVGDLVDLLIQNEFFAPASLLLPDAVPKTANTLPSKEAITVQQKQMPFC
DKDRTLMTPVQNLEQSYMPPDSSSPENKSLEVSDTRFHSFSFYELKNVTNNFDERPISVGG
NKMGEGGFGVVYKGYVNNTTVAVKKLAAMVDITTEELKQQFDQEIKVMAKCQHENLVELLGF
SSDGDDLCLVYVYMPNGSLLDRLSCLDGTPPLSWHMRCKIAQGAANGINFLHENHHIHRDIK
SANILLDEAFTAKISAFGLARASEKFAQTVMTSRIVGTTAYMAPEALRGEITPKSDIYSFGVVLL
EIITGLPAVDEHREPQLLLDIKEEIEDEEKTIEDYIDKKMNDADSTSVEAMYSVASQCLHEKKNK
RPDIKKVQQLLQEMTASSGSGGSGGSGGSDPPVATMVSKGEELFTGVVPILVELDGDVNGH
KFSVSGEGEGDATYGKLTLKFICTTGKLPVPWPTLVTTLTYGVQCFSRYPDHMKQHDFFKSA
MPEGYVQERTIFFKDDGNYKTRAEVKFEGDTLVNRIELKGIDFKEDGNILGHKLEYNYNSHNV
YIMADKQKNGIKVNFKIRHNIEDGSVQLADHYQQNTPIGDGPVLLPDNHYLSTQSKLSKDPNE
KRDHMVLLEFVTAAGITLGMDELYK

Human IRAK4-D329A-E-loop-mEGFP

MNKPITPSTYVRCLNVGLIRKLSDFIDPQEGWKKLAVAIKKPSGDDRYNQFHIRRFEALLQTGK
SPTSELLFDWGTTNCTVGDLVDLLIQNEFFAPASLLLPDAVPKTANTLPSKEAITVQQKQMPFC
DKDRTLMTPVQNLEQSYMPPDSSSPENKSLEVSDTRFHSFSFYELKNVTNNFDERPISVGG
NKMGEGGFGVVYKGYVNNTTVAVKKLAAMVDITTEELKQQFDQEIKVMAKCQHENLVELLGF
SSDGDDLCLVYVYMPNGSLLDRLSCLDGTPPLSWHMRCKIAQGAANGINFLHENHHIHRDIK
SANILLDEAFTAKISAFGLARASEKFAQEVMEERIVGTTAYMAPEALRGEITPKSDIYSFGVVLL
EIITGLPAVDEHREPQLLLDIKEEIEDEEKTIEDYIDKKMNDADSTSVEAMYSVASQCLHEKKNK
RPDIKKVQQLLQEMTASSGSGGSGGSGGSDPPVATMVSKGEELFTGVVPILVELDGDVNGH
KFSVSGEGEGDATYGKLTLKFICTTGKLPVPWPTLVTTLTYGVQCFSRYPDHMKQHDFFKSA
MPEGYVQERTIFFKDDGNYKTRAEVKFEGDTLVNRIELKGIDFKEDGNILGHKLEYNYNSHNV
YIMADKQKNGIKVNFKIRHNIEDGSVQLADHYQQNTPIGDGPVLLPDNHYLSTQSKLSKDPNE
KRDHMVLLEFVTAAGITLGMDELYK

Human IRAK4-A-loop-mEGFP

MNKPITPSTYVRCLNVGLIRKLSDFIDPQEGWKKLAVAIKKPSGDDRYNQFHIRRFEALLQTGK
SPTSELLFDWGTTNCTVGDLVDLLIQNEFFAPASLLLPDAVPKTANTLPSKEAITVQQKQMPFC
DKDRTLMTPVQNLEQSYMPPDSSSPENKSLEVSDTRFHSFSFYELKNVTNNFDERPISVGG
NKMGEGGFGVVYKGYVNNTTVAVKKLAAMVDITTEELKQQFDQEIKVMAKCQHENLVELLGF
SSDGDDLCLVYVYMPNGSLLDRLSCLDGTPPLSWHMRCKIAQGAANGINFLHENHHIHRDIK
SANILLDEAFTAKISDFGLARASEKFAQAVMAARIVGTTAYMAPEALRGEITPKSDIYSFGVVLL
EIITGLPAVDEHREPQLLLDIKEEIEDEEKTIEDYIDKKMNDADSTSVEAMYSVASQCLHEKKNK
RPDIKKVQQLLQEMTASSGSGGSGGSGGSDPPVATMVSKGEELFTGVVPILVELDGDVNGH
KFSVSGEGEGDATYGKLTLKFICTTGKLPVPWPTLVTTLTYGVQCFSRYPDHMKQHDFFKSA
MPEGYVQERTIFFKDDGNYKTRAEVKFEGDTLVNRIELKGIDFKEDGNILGHKLEYNYNSHNV
YIMADKQKNGIKVNFKIRHNIEDGSVQLADHYQQNTPIGDGPVLLPDNHYLSTQSKLSKDPNE
KRDHMVLLEFVTAAGITLGMDELYK

Human IRAK1 Wild Type-mScarlet

MAGGPGPGEPAAPGAQHFLYEVPPWVMCRFYKVMDALEPADWCQFAALIVRDQTELRLCE
RSGQRTASVLWPWINRNARVADLVHILTHLQLLRARDIITAWHPPAPLPSPGTTAPRPSSIPAPA
EAEAWSPRKLPSSASTFLSPAFPGSQTHSGPELGLVPSPASLWPPPPSPAPSSTKPGPESSV
SLLQGARPFPFCWPLCEISRGTHNFSEELKIGEGGFGCVYRAVMRNTVYAVKRLKENADLEW
TAVKQSFLTEVEQLSRFRHPNIVDFAGYCAQNGFYCLVYGFLPNGSLEDRLHCQTQACPPLS
WPQRLDILLGTARAIQFLHQDSPSLIHGDIKSSNVLLDERLTPKLGDFGLARFSRFAGSSPSQS

SMVARTQTVRGTLAYLPEEYIKTGRLAVDTDTFSFGVVVLETLAGQRAVKTHGARTKYLKDLV<br>EEEAEEAGVALRSTQSTLQAGLAADAWAAPIAMQIYKKHLDPRPGPCPPELGLGLGQLACCC<br>LHRRAKRRPPMTQVYERLEKLQAVVAGVPGHSEAASCIPPSPQENSYVSSTGRAHSGAAPW<br>QPLAAPSGASAQAAEQLQRGPNQPVESDESLGGLSAALRSWHLTPSCPLDPAPLREAGCPQ<br>GDTAGESSWGSGPGSRPTAVEGLALGSSASSSSEPPQIIINPARQKMVQKLALYEDGALDSL<br>QLLSSSSLPGLGLEQDRQGPEESDEFQSSGSGGSGGSGGSVSKGEAVIKEFMRFKVHMEG<br>SMNGHEFEIEGEGEGRPYEGTQTAKLKVTKGGPLPFSWDILSPQFMYGSRAFIKHPADIPDY<br>YKQSFPEGFKWERVMNFEDGGAVTVTQDTSLEDGTLIYKVKLRGTNFPPDGPVMQKKTMG<br>WEASTERLYPEDGVLKGDIKMALRLKDGGRYLADFKTTYKAKKPVQMPGAYNVDRKLDITSH<br>NEDYTVVEQYERSEGRHSTGGMDELYK
**Human IRAK2 Wild Type-mScarlet**
MACYIYQLPSWVLDDLCRNMDALSEWDWMEFASYVITDLTQLRKIKSMERVQGVSITRELLW<br>WWGMRQATVQQLVDLLCRLELYRAAQIILNWKPAPEIRCPIPAFPDSVKPEKPLAASVRKAED<br>EQEEGQPVRMATFPGPGSSPARAHQPAFLQPPEEDAPHSLRSDLPTSSDSKDFSTSIPKQE<br>KLLSLAGDSLFWSEADVVQATDDFNQNRKISQGTFADVYRGHRHGKPFVFKKLRETACSSP<br>GSIERFFQAELQICLRCCHPNVLPVLGFCAARQFHSFIYPYMANGSLQDRLQGQGGSDPLP<br>WPQRVSICSGLLCAVEYLHGLEIIHSNVKSSNVLLDQNLTPKLAHPMAHLCPVNKRSKYTMMK<br>THLLRTSAAYLPEDFIRVGQLTKRVDIFSCGIVLAEVLTGIPAMDNNRSPVYLKDLLLSDIPSSTA<br>SLCSRKTGVENVMAKEICQKYLEKGAGRLPEDCAEALATAACLCLRRRNTSLQEVCGSVAAV<br>EERLRGRETLLPWSGLSEGTGSSSNTPEETDDVDNSSLDASSSMSVAPWAGAATPLLPTEN<br>GEGRLRVIVGREADSSSEACVGLEPPQDVTETSWQIEINEAKRKLMENILLYKEEKVDSIELFG<br>PSGSGGSGGSGGSVSKGEAVIKEFMRFKVHMEGSMNGHEFEIEGEGEGRPYEGTQTAKLK<br>VTKGGPLPFSWDILSPQFMYGSRAFIKHPADIPDYYKQSFPEGFKWERVMNFEDGGAVTVTQ<br>DTSLEDGTLIYKVKLRGTNFPPDGPVMQKKTMGWEASTERLYPEDGVLKGDIKMALRLKDG<br>GRYLADFKTTYKAKKPVQMPGAYNVDRKLDITSHNEDYTVVEQYERSEGRHSTGGMDELYK
**Human IRAK3 Wild Type-mScarlet**
MAGNCGARGALSAHTLLFDLPPALLGELCAVLDSCDGALGWRGLAERLSSSWLDVRHIEKY<br>VDQGKSGTRELLWSWAQKNKTIGDLLQVLQEMGHRRAIHLITNYGAVLSPSEKSYQEGGFPN<br>ILFKETANVTVDNVLIPEHNEKGILLKSSISFQNIIEGTRNFHKDFLIGEGEIFEVYRVEIQNLTYA<br>VKLFKQEKKMQCKKHWKRFLSELEVLLLFHHPNILELAAYFTETEKFCLIYPYMRNGTLFDRL<br>QCVGDTAPLPWHIRIGILIGISKAIHYLHNVQPCSVICGSISSANILLDDQFQPKLTDFAMAHFRS<br>HLEHQSCTINMTSSSSKHLWYMPEEYIRQGKLSIKTDVYSFGIVIMEVLTGCRVVLDDPKHIQL<br>RDLLRELMEKRGLDSCLSFLDKKVPPCPRNFSAKLFCLAGRCAATRAKLRPSMDEVLNTLES<br>TQASLYFAEDPPTSLKSFRCPSPLFLENVPSIPVEDDESQNNNLLPSDEGLRIDRMTQKTPFE<br>CSQSEVMFLSLDKKPESKRNEEACNMPSSSCEESWFPKYIVPSQDLRPYKVNIDPSSEAPG<br>HSCRSRPVESSCSSKFSWDEYEQYKKESGSGGSGGSGGSVSKGEAVIKEFMRFKVHMEGS<br>MNGHEFEIEGEGEGRPYEGTQTAKLKVTKGGPLPFSWDILSPQFMYGSRAFIKHPADIPDYY<br>KQSFPEGFKWERVMNFEDGGAVTVTQDTSLEDGTLIYKVKLRGTNFPPDGPVMQKKTMGW<br>EASTERLYPEDGVLKGDIKMALRLKDGGRYLADFKTTYKAKKPVQMPGAYNVDRKLDITSHN<br>EDYTVVEQYERSEGRHSTGGMDELYK
