# Supplementary material for: IRAK4 autophosphorylation functions as a molecular switch to regulate death domain assembly and Myddosome maturation
Source: Nat Commun. 2026 Oct 21;17:11001. doi: 10.1038/s41467-026-78773-6 (PMC13687090; doi:10.1038/s41467-026-78773-6)
Supplement: Supplementary file 2 [file 41467_2026_78773_MOESM2_ESM.pdf]

nature portfolio

# Peer Review File

# IRAK4 autophosphorylation functions as a molecular switch to regulate death domain assembly and Myddosome maturation

Corresponding Author: Dr Marcus Taylor

**This file contains all reviewer reports in order by version, followed by all author rebuttals in order by version.**

Version 0:

Reviewer comments:

Reviewer #1

(Remarks to the Author)
This is an exciting study investigating the sequenced of events in myddosome formation in relation to IL-1 receptor signaling. They find that receptor clustering triggers Myd88 recruitment and then IRAK4 through DD interactions that are driven by mass action. IRAK4 autophosphorylation then enables recruitment of IRAK1, 2 and 3 through transient kinase domain interactions that enable hetero DD interactions to recruit effector kinases (IRAK1/2) or psuedokinase (IRAK3). This is an elegant mechanism to explain an additional switch to protect against constitutive signalling in the system with a trans-autophosphorylation following clustering being required to disinhibit the DD domain of the effector kinases or inhibitory pseudokinase. They build the case for this model in a logical sequence of experiments.

The paper is well focused. The only potential improvement I can think of is that it would be interesting to determine if cells expressing the kinase dead, phosphomimetic activation loop IRAK4 are fully IL-1 dependent for downstream signaling. This could be investigated as a function of IRAK4 expression level n relation to single cell analysis of IL-2 production through intracellular cytokine staining or using a local cytokine capture/dection kit (Miltenyi). If the kinase independent contruct is subject to basal signaling when expressed at a higher level this would support a role for the switch in safeguarding such powerful pro-inflammatory and prosurvival signals.

Minor correction- line 330- confirmation to conformation.

(Remarks on code availability)

Reviewer #2

(Remarks to the Author)
In this manuscript, the authors investigated how the IRAKs incorporated into the Mydosome to activate the innate immune response. They found the IRAK4 kinase enzymatic activity and its autophosphorylation play crucial roles for the incorporation of IRAK1/2/3 into Myddosome. Overall, these results are impressive. However, some key issues should be addressed before consideration for publication in Nature Communications.
Major issues:
1. In result 1, the authors found that the loss of the IRAK4 kinase domain didn't impact the co-assembly of MyD88:IRAK4. But, in the Movie S1, why the puncta of the wild-type IRAK4 was larger than the IRAK4-Delta Kinase Domain? This movie showed that the loss of the IRAK4 kinase domain might inhibit the aggregation level of IRAK4. The authors should explain this phenomenon and design experiments to eliminate potential impacts.
2. The process of Myddosome assembly and maturation was similar with liquid-liquid phase separation (LLPS), especially the puncta formation of IRAK4, IRAK1/2/3. So, whether the LLPS process was involved in Myddosome assembly and maturation?
3. In addition to IL-1 signaling pathway mediating MyD88 assembly, Toll-like receptor signaling also could trigger MyD88 assembly, such as LPS stimulating TLR4 pathway. So, whether the IRAK4 kinase domain and enzymatic activity played the

same function during TLR signaling? The authors could over-express TLR4 in EL4-3xKO cells which had reconstituted with different wild type or mutant IRAK4, IRAK1/2/3, and then stimulated with LPS and tested the Myddosome assembly and maturation.
Minor issues:
1. Figure 1C and 1D, the title of figure was wrong. “IRAK4-IRAK1” should change to “MyD88-IRAK4”.

2.The graph of Figure 1B showed IRAK1 was involved in this assay, but IRAK1 did not affect the interaction between MyD88 and IRAK4. So, the image of IRAK1 in Figure 1B should be deleted.

(Remarks on code availability)

Version 1:

Reviewer comments:

Reviewer #1

(Remarks to the Author)
The authors have addressed my question. They have found that the kinase dead, phosphomimetic mutant is fully IL-1 dependent for signaling. They also show that even cells expressing high levels of the GFP tagged IRAK4 constructs didn't signal basally. They have also addressed the concerns of reviewer 2. I have no further questions.

(Remarks on code availability)
I'm not in a position to review or test this, but I would expect the author to be very reliable in providing useful tools for the field.

Reviewer #2

(Remarks to the Author)
The authors addressed all my concerns, I have no further coments and concerns.

(Remarks on code availability)

**Open Access** This Peer Review File is licensed under a Creative Commons Attribution 4.0 International License, which permits use, sharing, adaptation, distribution and reproduction in any medium or format, as long as you give appropriate credit to the original author(s) and the source, provide a link to the Creative Commons license, and indicate if changes were made.
In cases where reviewers are anonymous, credit should be given to 'Anonymous Referee' and the source.
The images or other third party material in this Peer Review File are included in the article’s Creative Commons license, unless indicated otherwise in a credit line to the material. If material is not included in the article’s Creative Commons

license and your intended use is not permitted by statutory regulation or exceeds the permitted use, you will need to obtain permission directly from the copyright holder.
To view a copy of this license, visit https://creativecommons.org/licenses/by/4.0/

**Response to the Referees**

We thank the referees for their generous comments and helpful suggestions on the manuscript. We are delighted that the referees find the manuscript and findings important and exciting.

In the revised manuscript, we have added additional data. In Figure 4, we added equivalent single-molecule microscopy data for the IRAK3 kinase domain. We therefore find that, like the kinase domains of IRAK1 and IRAK2, the IRAK3 kinase domain is recruited to nascent Myddosome assemblies in an IRAK4 kinase-dependent manner (Fig. 4i through k). This data strengthens the core finding that IRAK4 autophosphorylation is a molecular switch that activates Myddosome maturation and downstream signaling.

In addition, to address the point raised by Reviewer #1 about whether the kinase domain influences the oligomerization of IRAK4, we analyzed our data to compare the stability and oligomeric state of IRAK4, comparing WT and IRAK4-ΔKD. We found no difference in the distribution of oligomer size or stability (Supplemental Fig. 1e through h). This again supports the conclusion that the IRAK4 kinase domain operates downstream of MyD88–IRAK4 co-assembly (a process controlled by DD co-assembly).

**Our reply is in blue below**

**REVIEWER COMMENTS**

Reviewer #1 (Remarks to the Author):

This is an exciting study investigating the sequence of events in myddosome formation in relation to IL-1 receptor signaling. They find that receptor clustering triggers Myd88 recruitment and then IRAK4 through DD interactions that are driven by mass action. IRAK4 autophosphorylation then enables recruitment of IRAK1, 2 and 3 through transient kinase domain interactions that enable hetero DD interactions to recruit effector kinases (IRAK1/2) or psuedokinase (IRAK3). This is an elegant mechanism to explain an additional switch to protect against constitutive signalling in the system with a trans-autophosphorylation following clustering being required to disinhibit the DD domain of the effector kinases or inhibitory pseudokinase. They build the case for this model in a logical sequence of experiments.

The paper is well focused. The only potential improvement I can think of is that it would be interesting to determine if cells expressing the kinase dead, phosphomimetic activation loop IRAK4 are fully IL-1 dependent for downstream signaling. This could be investigated as a function of IRAK4 expression level in relation to single cell analysis of IL-2 production through intracellular cytokine staining or using a local cytokine capture/detection kit (Miltenyi). If the kinase independent construct is subject to basal signaling when expressed at a higher level this would support a role for the switch in safeguarding such powerful pro-inflammatory and prosurvival signals.

We thank the reviewer for recognizing the clarity and focus of the manuscript. In short: we tested this directly, and across the expression range we could stably establish, the kinase-

dead-phosphomimetic activation-loop mutant showed no constitutive activity and downstream signaling remained fully IL-1 dependent, as the reviewer asked. Whether basal signaling emerges at expression levels above endogenous IRAK4 remains open, because we were unable to stably establish lines expressing the mutant in that range. We provide these data here for the referee's and editor's assessment, but propose not to include them in the manuscript, for the reasons set out below.

The reviewer's suggestion speaks to a broader question: how the cytoplasmic concentration of signalosome components is set high enough for rapid triggering yet low enough to suppress basal activity. This question extends beyond the Myddosome to other death-domain signalosomes, and is one we are continuing to pursue.

To begin to address it here, we used FACS to sort cells expressing GFP-tagged IRAK4 alleles into matched high- and low-expression populations, then measured basal and IL-1–inducible signaling in each by IL-2 ELISA. Because GFP is fused directly in-frame to IRAK4 rather than expressed as a separate product, GFP fluorescence intensity reports the abundance of the IRAK4-GFP fusion protein itself, allowing FACS gating on GFP to select directly for IRAK4 expression level. At the expression levels we were able to establish stably, the mutant did not show elevated basal signaling compared with WT, and downstream signaling remained IL-1 dependent.

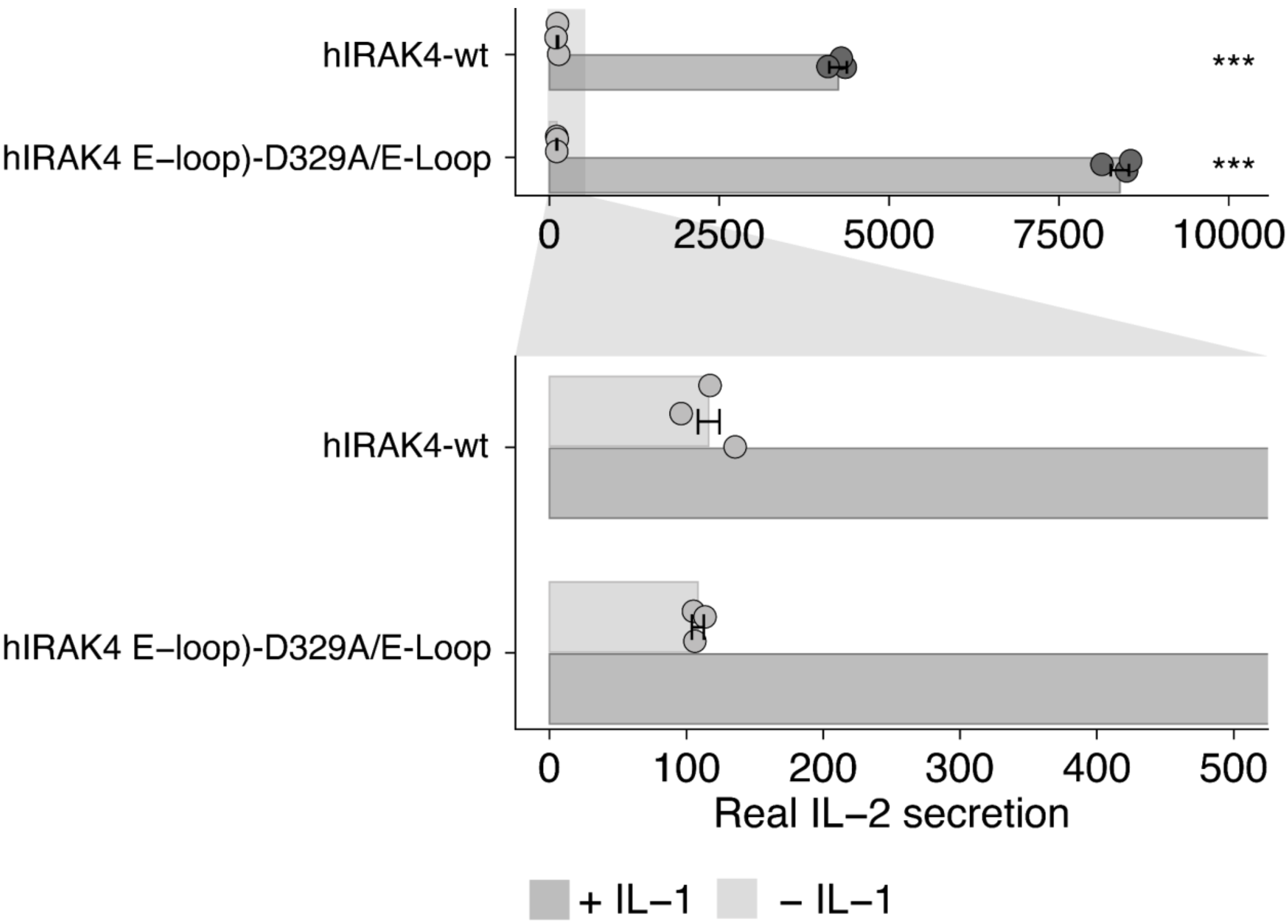


***IL-2 release in EL4-MyD88-GFP/IRAK4-KO cells reconstituted with hIRAK4-WT or hIRAK4-D329A/E-Loop mutant. Values shown are real IL-2 release (n = 3 independent replicates measured per cell line). Inset provides a view of IL-2 release in unstimulated conditions.***

We note the reviewer's specific suggestion of single-cell readouts, by intracellular cytokine staining or local cytokine capture. We agree these would give greater resolution than bulk ELISA and would adopt them in a system where the relevant comparison could be made. In the present setting, however, the limiting factor is not the resolution of the readout but the expression range accessible to us: single-cell analysis of populations that cannot be held above endogenous expression would not address the reviewer's underlying question.

We would caution against reading our result as evidence that the mutant cannot signal basally, because it is bounded by the expression range we could sample. That is, expression levels up to, but not exceeding, endogenous IRAK4. The mechanism we propose predicts as much. IRAK4 kinase homodimerization and autophosphorylation occur only upon co-assembly with MyD88, as shown here and previously (Ferrao et al. 2014), implying that the relevant homo- and hetero-dimeric interactions are weak and non-permissive in the bulk cytosol, becoming activating only upon death-domain oligomerization at the assembling Myddosome. On this model, quiescence at the levels we assayed is expected, but basal signaling could emerge at higher expression, where increased concentration begins to favor assembly.

That high-expression regime is precisely the one we could not sample. We were consistently unable to establish lines stably expressing IRAK4 mutants above the endogenous IRAK4 level (as measured against gene-edited cells expressing GFP-tagged IRAK4 alleles), a ceiling that did not constrain WT IRAK4-GFP, which we could stably express above endogenous. Mutant-expressing cells initially FACS-sorted for the highest GFP expression consistently reverted to endogenous-or-lower expression within days in culture. This consistent, unidirectional shift suggests that sustained high-level expression of the mutant is not merely neutral but may be actively counter-selected. We have not characterized the mechanism, and we raise this cautiously, but we note that counter-selection against high-level expression of a kinase-dead-phosphomimetic allele is the behavior the reviewer's own hypothesis would predict: low-level constitutive signaling through a powerful pro-inflammatory and pro-survival pathway would be expected to impose a fitness cost. We regard this as suggestive rather than evidential.

These data therefore reflect the expression levels of cells that survived selection, and cannot on their own exclude expression-dependent basal signaling at higher, currently inaccessible levels. What they do address is the reviewer's question of inducibility: across the range we could stably assay, the mutant behaved like WT IRAK4 and remained fully IL-1 dependent. Because they cannot resolve the expression-dependent question, we have not added them to the main text. Resolving it will require inducible, titratable expression systems in which levels can be matched between WT and mutant alleles, decoupling expression from clonal selection and long-term viability, combined with the single-cell readouts the reviewer suggests. We believe future work is needed to understand how control mechanisms such as IRAK4 autophosphorylation are coordinated with the cytoplasmic abundance of the signaling effectors.

Minor correction- line 330- confirmation to conformation.

We thank for the reviewer for careful proofreading of the text and have made this correction

Reviewer #2 (Remarks to the Author):

In this manuscript, the authors investigated how the IRAKs incorporated into the Mydosome to activate the innate immune response. They found the IRAK4 kinase enzymatic activity and its autophosphorylation play crucial roles for the incorporation of IRAK1/2/3 into Myddosome. Overall, these results are impressive. However, some key issues should be addressed before consideration for publication in Nature Communications.
Major issues:

1. In result 1, the authors found that the loss of the IRAK4 kinase domain didn't impact the co-assembly of MyD88:IRAK4. But, in the Movie S1, why the puncta of the wild-type IRAK4 was larger than the IRAK4-Delta Kinase Domain? This movie showed that the loss of the IRAK4 kinase domain might inhibit the aggregation level of IRAK4. The authors should explain this phenomenon and design experiments to eliminate potential impacts.

To address whether the IRAK4 kinase domain contributes to its aggregation, we analyzed the lifetime and size distribution of MyD88 and IRAK4 puncta. We found that the presence or absence of the IRAK4 kinase domain had no effect on the lifetime distribution of either MyD88 or IRAK4 puncta (average lifetime for IRAK4-WT vs. IRAK4-ΔKD was 55 vs. 51 s, respectively). The proportion of MyD88 and IRAK4 puncta equal to or exceeding a stoichiometry consistent with the solved Myddosome structure was also equivalent between IRAK4-WT and IRAK4-ΔKD. That is, 15% vs 12% of IRAK4-WT and IRAK4-ΔKD puncta respectively reached a fluorescence intensity greater than or equal to that of 4 molecules (the copy number of IRAK4 in the solved Myddosome structure). This new analysis is included in Supplemental Fig. 1e–h and confirms that IRAK4-WT and IRAK4-ΔKD puncta are equivalent in size.

We agree that the IRAK4-WT puncta in the original Supplementary Movie 1 appeared larger than those of the IRAK4-ΔKD example, and we thank the reviewer for raising this point. As the quantification above shows, that appearance reflects the particular example cells shown rather than a property of the dataset: the example movies suggest a difference in IRAK4-WT and IRAK4-ΔKD puncta size and stability that is not borne out by the ensemble data. We have therefore replaced the IRAK4-ΔKD example in Supplementary Movie 1 with a cell that better represents the ΔKD population. The revised Supplementary Movie 1 shows a representative example of each IRAK4 allele, consistent with the analysis showing no discernible difference in puncta size or stability between IRAK4-WT and IRAK4-ΔKD.

2. The process of Myddosome assembly and maturation was similar with liquid-liquid phase separation (LLPS), especially the puncta formation of IRAK4, IRAK1/2/3. So, whether the LLPS process was involved in Myddosome assembly and maturation?

We agree with the reviewer that the macroscopic phenomenon of Myddosome assembly appears to bear the hallmark of liquid-liquid phase separation. However, The Myddosome resolved in the crystal structure is a discrete helical assembly with defined subunit stoichiometry, in which MyD88, IRAK4 and IRAK2 are present at fixed copy number (Lin et al. 2010; Moncrieffe et al. 2020). This is the central reason we do not favor a phase-separation mechanism: liquid–liquid demixing produces condensates whose composition scales with concentration and whose internal arrangement is disordered and continuously

rearranging, whereas the Myddosome is a stoichiometrically defined, ordered polymer. We have previously tested physical properties of Myddosome in cells and found that they have high stability, with no exchange of components with the cytosol. This was previously assayed by FRAP and published in (Deliz-Aguirre et al. 2021). We recently showed that an essential functional motif of death domain oligomers is their high-stability, and that this allows them to recruit and activate TRAF6 (an essential downstream signaling adaptor) (Lichtenstein et al. 2025). We therefore favor a model in which Myddosome assembly is more akin to crystalline deposition than to liquid-liquid demixing: a stepwise, ordered nucleation and growth process that yields a stable, non-dynamic structure, rather than a liquid condensate with continuous internal rearrangement and exchange. Given these published findings from our lab, we consider LLPS an unlikely mechanism for the assembled and mature Myddosome. We note that this evidence speaks to the stability and non-dynamic character of the assembled structure rather than formally excluding a transient demixing step at nucleation, which would be challenging to capture directly; we nonetheless favor an ordered nucleation-and-growth model over liquid–liquid demixing for the process as a whole.

3. In addition to IL-1 signaling pathway mediating MyD88 assembly, Toll-like receptor signaling also could trigger MyD88 assembly, such as LPS stimulating TLR4 pathway. So, whether the IRAK4 kinase domain and enzymatic activity played the same function during TLR signaling? The authors could over-express TLR4 in EL4-3xKO cells which had reconstituted with different wild type or mutant IRAK4, IRAK1/2/3, and then stimulated with LPS and tested the Myddosome assembly and maturation.

We thank the reviewer for this suggestion and agree that examining Myddosome assembly in the context of TLR stimulation is important for understanding whether different upstream biochemical contexts shape downstream mechanistic features. These questions are under active investigation in our lab.

We would first like to direct the reviewer to a recent preprint from a collaboration with the Majer group (Dingfelder et al. 2025), in which we examine Myddosome formation downstream of the intracellular receptor TLR7. In primary macrophages, we found that an IRAK4 kinase inhibitor blocked downstream cytokine release following TLR7, TLR9, and TLR4 stimulation, consistent with reports from immortalized macrophage lines (De Nardo et al. 2018). This argues that IRAK4 performs an equivalent function across distinct TLR contexts, directly addressing the reviewer's underlying question. Notably, Dingfelder et al. work also shows that the threshold for Myddosome nucleation can be tuned, and a key future direction is to determine whether IRAK4 and the other IRAK paralogs influence the kinetics of Myddosome assembly across receptor systems and diverse cellular contexts.

We also wish to explain why the specific experiment proposed (reconstituting TLR4 signaling in our EL4-3xKO line) is unlikely to yield an interpretable result without substantial additional engineering. Unlike the IL-1R and intracellular TLR pathways, TLR4 signaling uses the adaptor TIRAP (Mal) for Myddosome nucleation, and TIRAP is expressed at undetectable levels in EL4 cells (unpublished data from our lab). Productive LPS recognition upstream of TLR4 further depends on a dedicated accessory machinery, including the co-receptor MD-2, which binds LPS and is required for TLR4 activation, as well as CD14 and LBP. Reconstituting this entire LPS recognition system alongside TIRAP would introduce so many engineered variables that it would be difficult to attribute any observed phenotype specifically

to the IRAK4 kinase domain or its enzymatic activity, which is the question at hand. We therefore believe that these TLR7 results (Dingfelder et al. 2025), together with the findings of the present study and other published work (De Nardo et al. 2018), support the conclusion of an equivalent functional role for IRAK4 across TLR and IL-1R signaling, which we are continuing to test directly in ongoing work. Critically, the present study here also supplies the mechanistic basis for this shared requirement, explaining how IRAK4 and its kinase activity govern signal transduction through the Myddosome by inducing the recruitment of IRAK1/2/3.

In the manuscript we are careful to frame our result in the context of IL-1R activation and Myddosome formation. In future studies we will explore how these mechanisms operate in TLRs. To address these questions related to TLRs rigorously, we are currently generating additional primary and immortalized cell lines that allow us to dissect the contributions of IRAK4 and the other IRAKs to Myddosome assembly, as well as the general mechanisms governing this critical signaling event.

Minor issues:
1. Figure 1C and 1D, the title of the figure was wrong. “IRAK4-IRAK1” should change to “MyD88-IRAK4”.

We thank the reviewer for careful proofreading of the figures and have amended the figure to be correctly labelled.

2.The graph of Figure 1B showed IRAK1 was involved in this assay, but IRAK1 did not affect the interaction between MyD88 and IRAK4. So, the image of IRAK1 in Figure 1B should be deleted.

We may not have made the purpose of these schematics sufficiently clear. The schematics in Fig. 1b are not intended to indicate which components functionally contribute to the MyD88–IRAK4 interaction; rather, they illustrate which Myddosome components (and their mutant or WT alleles) are expressed in the cell line being assayed (in this example EL4-MyD88-GFP/IRAK4-KO reconstituted with IRAK4-ΔKD, and still expressing IRAK1). This convention is used throughout the figures. In the example highlighted, IRAK1 is included for this reason: it is expressed in the assayed cell line, irrespective of whether it affects MyD88–IRAK4 binding. In nearly all cases, we performed two-colour imaging, with two out of three Myddosome components tagged, so one component was necessarily untagged and is not shown; this is a labelling constraint, not a statement about functional relevance. We believe this convention guides the reader and increases the accessibility of the figures, but we recognize that opinions may differ on figure presentation, and we defer to the editor's judgment on whether IRAK1 should remain in the panel.

Cited Reference:

Deliz-Aguirre, Rafael, Fakun Cao, Fenja H. U. Gerpott, et al. 2021. “MyD88 Oligomer Size Functions as a Physical Threshold to Trigger IL1R Myddosome Signaling.” *The Journal of Cell Biology* 220 (7). https://doi.org/10.1083/jcb.202012071.

De Nardo, Dominic, Katherine R. Balka, Yamel Cardona Gloria, Vikram R. Rao, Eicke Latz,

and Seth L. Masters. 2018. “Interleukin-1 Receptor-Associated Kinase 4 (IRAK4) Plays a Dual Role in Myddosome Formation and Toll-like Receptor Signaling.” *The Journal of Biological Chemistry* 293 (39): 15195–15207.

Dingfelder, Anna, Fenja Blank, Noemie Quinson, et al. 2025. “Microbial Priming Enhances TLR7-Driven Myddosome Assembly Dynamics.” In *bioRxiv*. BioRxiv, December 18. https://doi.org/10.64898/2025.12.16.693659.

Ferrao, Ryan, Hao Zhou, Yibing Shan, et al. 2014. “IRAK4 Dimerization and Trans-Autophosphorylation Are Induced by Myddosome Assembly.” *Molecular Cell* 55 (6): 891–903.

Lichtenstein, Mauriz A., Fakun Cao, Finn Lobnow, et al. 2025. “Bottom-up Reconstruction of Functional Death Fold Signalosomes Reveals a Requirement for Polymer Stability and Avidity.” *Science (New York, N.Y.)* 388 (6745): 415–422.
