# Supplementary material for: IRAK4 autophosphorylation functions as a molecular switch to regulate death domain assembly and Myddosome maturation
Source: Nat Commun. 2026 Oct 21;17:11001. doi: 10.1038/s41467-026-78773-6 (PMC13687090; doi:10.1038/s41467-026-78773-6)
Supplement: Supplementary file 4 [file 41467_2026_78773_MOESM4_ESM.pdf]

Description of Additional Supplementary Files

Supplementary Movie 1

Description: IRAK4:IRAK1 co-assembly requires the IRAK4 kinase domain.

Supplementary Movie 2

Description: IRAK1 kinase domain is dispensable for IRAK4:IRAK1 co assembly.

Supplementary Movie 3

Description: IRAK4:IRAK2 and IRAK4:IRAK3 co-assembly requires the IRAK4 kinase domain.

Supplementary Movie 4

Description: The IRAK2 and IRAK3 kinase domain is dispensable for co-assembly with IRAK4.

Supplementary Movie 5

Description: IRAK4 kinase activity is required for stable co-assembly with IRAK1.

Supplementary Movie 6

Description: The kinase domain of IRAK1, independent of the DD, can be recruited to the partially assembled Myddosome.

Supplementary Movie 7

Description: A phosphomimetic mutation can restore stable IRAK1 co-assembly to a kinase inactive IRAK4 mutant.
